# Listening in the Moment: How Bilingualism Interacts With Task Demands to Shape Active Listening

**DOI:** 10.3389/fnins.2021.717572

**Published:** 2021-12-10

**Authors:** Jennifer Krizman, Adam Tierney, Trent Nicol, Nina Kraus

**Affiliations:** ^1^Auditory Neuroscience Laboratory, Department of Communication Sciences and Disorders, Northwestern University, Evanston, IL, United States; ^2^The ALPHALAB, Department of Psychological Sciences, Birkbeck, University of London, London, United Kingdom; ^3^Departments of Neurobiology and Otolaryngology, Northwestern University, Evanston, IL, United States

**Keywords:** attention, listening, language, bilingualism, auditory

## Abstract

While there is evidence for bilingual enhancements of inhibitory control and auditory processing, two processes that are fundamental to daily communication, it is not known how bilinguals utilize these cognitive and sensory enhancements during real-world listening. To test our hypothesis that bilinguals engage their enhanced cognitive and sensory processing in real-world listening situations, bilinguals and monolinguals performed a selective attention task involving competing talkers, a common demand of everyday listening, and then later passively listened to the same competing sentences. During the active and passive listening periods, evoked responses to the competing talkers were collected to understand how online auditory processing facilitates active listening and if this processing differs between bilinguals and monolinguals. Additionally, participants were tested on a separate measure of inhibitory control to see if inhibitory control abilities related with performance on the selective attention task. We found that although monolinguals and bilinguals performed similarly on the selective attention task, the groups differed in the neural and cognitive processes engaged to perform this task, compared to when they were passively listening to the talkers. Specifically, during active listening monolinguals had enhanced cortical phase consistency while bilinguals demonstrated enhanced subcortical phase consistency in the response to the pitch contours of the sentences, particularly during passive listening. Moreover, bilinguals’ performance on the inhibitory control test related with performance on the selective attention test, a relationship that was not seen for monolinguals. These results are consistent with the hypothesis that bilinguals utilize inhibitory control and enhanced subcortical auditory processing in everyday listening situations to engage with sound in ways that are different than monolinguals.

## Introduction

Language experience leaves a pervasive imprint on the brain. Auditory-based language exposure not only supports language acquisition, but also facilitates the development of executive functions, namely inhibitory control (Wolfe and Bell, 2004; Figueras et al., 2008), working memory (Figueras et al., 2008; Conway et al., 2009; Gathercole and Baddeley, 2014), and sustained attention (Mitchell and Quittner, 1996; Khan et al., 2005). Through their interconnected development, the executive and auditory systems become strongly tethered (Baddeley, 2003; Kral et al., 2016). This cognitive-sensory link is universal across spoken languages (Weissman et al., 2006; Wu et al., 2007), is supported by functional and structural connections between auditory and executive systems (Jürgens, 1983; Casseday et al., 2002; Raizada and Poldrack, 2007), and aids in focusing the attentional searchlight on a target sound (Fritz et al., 2007; Pichora-Fuller et al., 2016).

While language exposure facilitates development of auditory and executive systems in everyone (Conway et al., 2009; Kronenberger et al., 2020), the experience of learning two languages results in additional strengthening of the executive system in bilinguals (Mechelli et al., 2004; Abutalebi and Green, 2007; Abutalebi et al., 2011; Gold et al., 2013; Costa and Sebastián-Gallés, 2014). This strengthening is believed to result from the constant co-activation of both of their languages during communication (Spivey and Marian, 1999; Marian and Spivey, 2003; Thierry and Wu, 2007) and the resultant need to suppress the irrelevant language (Kroll et al., 2008; Van Heuven et al., 2008). Through the daily practice of selectively inhibiting one language, bilinguals fine-tune their inhibitory control ability (Bialystok and Viswanathan, 2009; Foy and Mann, 2014), an executive function that focuses attention on a relevant stimulus amid distractors. There is evidence that this daily tuning leads to bilingual advantages, relative to monolinguals, on tasks assessing inhibitory control [reviewed in Bialystok (2011), though some have failed to replicate this advantage, Paap et al., 2015; Dick et al., 2019].

In addition to aiding bilinguals in juggling their two languages, inhibitory control is important for all listeners during everyday communication. Everyday communication often takes place in noisy environments, requiring a listener to focus on a target talker amid distractors. When perceiving speech in noise, inhibitory control operates in concert with auditory processing to suppress irrelevant and enhance the representation of relevant stimulus features important for discriminating a target object from other sounds (Neill et al., 1995; Alain and Woods, 1999; Hopfinger et al., 2000; Tun et al., 2002; Wu et al., 2007). Because it contributes to pitch perception and aids in separating a target talker from distractors, the fundamental frequency (F0) is an important cue for perceiving speech in noise (Bregman et al., 1990; Darwin, 1997; F0; Bird and Darwin, 1998; Darwin et al., 2003). Indeed, more robust subcortical encoding of the F0, as measured by the frequency-following response (FFR), a neurophysiological response to sound generated predominately in the inferior colliculus (Chandrasekaran and Kraus, 2010; Coffey et al., 2016, 2019; Bidelman, 2018; White-Schwoch et al., 2019), relates with better speech-in-noise abilities (Anderson et al., 2010; Song et al., 2010). F0 encoding is malleable with language experience (Song et al., 2008; Krishnan et al., 2009). For example, bilinguals show enhanced subcortical encoding of the F0 that relates with their heightened inhibitory control ability (Krizman et al., 2012).

Given that bilinguals show enhancements in processes important for listening in the crowded acoustic environments commonly encountered in daily life, bilinguals would be expected to also show heightened speech-in-noise recognition. Previous literature, however, has found bilinguals struggle in this realm relative to monolinguals (Mayo et al., 1997; Cooke et al., 2008; Lecumberri et al., 2011; Lucks Mendel and Widner, 2016; Krizman et al., 2017; Morini, 2020). Despite bilinguals’ cognitive and sensory enhancements, they perform more poorly than monolinguals on clinical assessments of listening to speech in noise (Shi, 2010, 2012; Stuart et al., 2010; Krizman et al., 2017; Skoe and Karayanidi, 2019). Interestingly, this perception-in-noise disadvantage only manifests when the target is linguistic; bilinguals instead show an advantage when the target is non-linguistic (i.e., a tone; Krizman et al., 2017). Given that bilinguals display impaired recognition in noise only for linguistic stimuli, and that bilinguals have enhanced inhibitory control (Bialystok, 2011) and F0 encoding (Krizman et al., 2012; Skoe et al., 2017), the bilingual speech-in-noise disadvantage may stem from difficulties with linguistic processing, which bilinguals may try to compensate for, at least partly, by strengthening the cognitive and sensory processes involved in these tasks (Crittenden and Duncan, 2014; Krizman et al., 2017; Skoe, 2019).

Given the evidence for enhanced inhibitory control and F0 encoding in bilinguals, these advantages may evince possible strategies for listening in noise that are uniquely successful for bilinguals. We hypothesize that the enhancements in inhibitory control and F0 encoding are the byproduct of continued reliance on these processes during everyday listening and reflect differences between monolinguals and bilinguals in how they understand speech, particularly degraded speech, such as speech in noise. To test whether bilinguals and monolinguals differ in the processes engaged to understand speech in noise, high-proficiency bilingual speakers of Spanish and English and monolingual speakers of English performed a selective attention task in which they were instructed to focus on one of two competing talkers, similar to the demands of everyday listening environments. We measured behavioral indices of task performance, and the neural processes engaged during the selective attention task were compared to neural processes engaged when the participants passively listened to these same competing sentences. Behaviorally, participants used a button box to select the correct button as instructed by the target talker amid competing instructions from the distracting talker. We predicted that bilinguals would perform more poorly than monolinguals on this task, consistent with bilinguals’ poorer performance on speech-in-noise tests (Mayo et al., 1997; Shi, 2010, 2012).

Neurally, we used EEG to measure cortical and subcortical brain responses during the selective listening test and during passive exposure to the test sentences. We measured cortical neural entrainment across multiple frequency bands over the duration of the competing sentences and subcortical neural entrainment to the pitch contour of each talker. Cortically, active listening during a selective attention task increases neural entrainment relative to passive listening (Mesgarani and Chang, 2012; Golumbic et al., 2013; Ding and Simon, 2014). Evidence suggests that selective attention engages distinct cortical networks in bilinguals and monolinguals (Olguin et al., 2019); however, it is unknown whether levels of cortical neural entrainment engaged during active or passive listening differs between these groups. Given the reported mechanistic differences (Astheimer et al., 2016; Olguin et al., 2019), we predicted that cortical neural entrainment during active listening would differ between bilinguals and monolinguals, while the groups would be matched during passive listening.

In contrast to cortical entrainment, work in animal models has shown that active listening decreases subcortical auditory encoding relative to passive listening (Slee and David, 2015). Despite these findings, the prevailing view in humans is that differences between active and passive listening are minimal or non-existent given early work showing a lack of attention effects on subcortical responses to simple auditory stimuli (e.g., clicks; Salamy and McKean, 1977; Collet and Duclaux, 1986) and the fact that, unlike cortical responses, subcortical responses can be reliably acquired whether the participant is awake or asleep (Osterhammel et al., 1985; Krishnan et al., 2005). Studies in humans have instead focused on whether differences can be seen in the subcortical response to the attended versus the ignored auditory stream during an active listening task, yielding mixed results (Galbraith et al., 1998; Varghese et al., 2015; Forte et al., 2017). As a first step to understanding the influence attention has on subcortical encoding during everyday listening situations and whether language experience impacts this influence, we wanted to focus on the general effect of attention on listening. Therefore, rather than comparing responses to the attended and ignored streams, we compared the groups when they were actively and passively listening to the talkers. Across all participants, we predicted that active listening would lead to a reduction in subcortical neural entrainment relative to passive listening, consistent with findings in animals (Slee and David, 2015). Moreover, given the previously reported bilingual enhancements in subcortical F0 encoding (Krizman et al., 2012; Skoe et al., 2017), we predicted that bilinguals would demonstrate greater subcortical neural entrainment to the pitch of the stimuli in both the active and passive listening conditions relative to monolinguals.

Separate from the selective-attention task, we tested participants on a measure of inhibitory control to determine whether inhibitory control abilities support performance on the selective attention task (Bialystok, 2015). We predicted bilinguals would outperform monolinguals on the inhibitory control measure, consistent with previous studies (Bialystok and Martin, 2004; Carlson and Meltzoff, 2008; Bialystok, 2009; de Abreu et al., 2012; Krizman et al., 2012, 2014). Additionally, because inhibitory control has been found to facilitate listening to a target talker during selective attention tasks (Alain and Woods, 1999; Tun et al., 2002), we predicted that performance on the inhibitory control and selective attention tasks would relate in both monolinguals and bilinguals. However, if bilinguals rely more heavily on inhibitory control during real-world listening (Krizman et al., 2012, 2017; Bialystok, 2015), then we expect this relationship between inhibitory control and active listening to be stronger in bilinguals.

## Materials and Methods

### Participants

Participants were 40 adolescents and young adults [18.09 ± 0.64 years of age, 22 female, 19 low socioeconomic status (as indexed by maternal education, Hollingshead, 1975)], recruited from four Chicago high schools. The Northwestern University Institutional Review Board approved all procedures and consent was provided by participants 18 and older while informed written assent was given by adolescents younger than 18 and consent provided by their parent/guardian. Participants were monetarily compensated for their participation.

Participants were English monolinguals (*n* = 20; 55% female) and high-proficiency Spanish–English bilinguals (*n* = 20; 55% female) as measured by the Language Experience and Proficiency Questionnaire (LEAP-Q, Marian et al., 2007; Kaushanskaya et al., 2019). Maternal education level was used to approximate socioeconomic status (Hart and Risley, 1995; Hoff, 2003; D’Angiulli et al., 2008). Half of the monolinguals and 45% of the bilinguals had mothers with education levels of high-school graduate or below, while the remaining participants’ maternal education levels were some college or beyond. To be included in this study, participants in both groups needed to have high English proficiency (≥7 out of 10 on English speaking and understanding proficiency, LEAP-Q). The monolinguals were required to have low Spanish proficiency (≤4 out of 10 on Spanish speaking and understanding proficiency, LEAP-Q), while the bilinguals were required to have high Spanish proficiency (≥6 out of 10 on Spanish speaking and understanding proficiency, LEAP-Q). Bilinguals were further required to have early acquisition of Spanish and English (≤5 years old). All subjects were required to have air conduction thresholds of <20 dB hearing level (HL) per octave for octaves from 125 to 8000 Hz and no diagnosis of a reading or language disorder. The two groups were matched on age [monolinguals: 18.07 ± 0.59 years, bilinguals: 18.12 ± 0.71 years; *F*(1,38) = 0.049, *p* = 0.825, η_p_^2^ = 0.001], sex (Kruskal–Wallis X^2^ = 0, *p* = 0.999), maternal education level (Kruskal–Wallis X^2^ = 0.098, *p* = 0.755), IQ [monolinguals: 104.65 ± 7.62; bilinguals: 101.65 ± 12.40; *F*(1,38) = 0.850, *p* = 0.362, η_p_^2^ = 0.022, Wechsler Abbreviated Scale of Intelligence, WASI, Wechsler, 1999], and English proficiency [*F*(1,38) = 0.496, *p* = 0.486, η_p_^2^ = 0.013], as determined from the LEAP-Q. As shown in Table 1, the groups differed on amount of daily English/Spanish exposure [*F*(1,38) = 89.24, *p* < 0.0005, η_p_^2^ = 0.701] and Spanish proficiency [*F*(1,38) = 283.72, *p* < 0.0005, η_p_^2^ = 0.882].

**TABLE 1:** Language measures for English and Spanish in monolinguals and bilinguals.

		English	Spanish
English Monolingual	Age of acquisition	1.3 ± 1.34 years	n/a
	Proficiency	9.55 ± 0.67	0.78 ± 1.41
	Exposure	95.25 ± 8.81	4.75 ± 8.81
Spanish–English Bilingual	Age of acquisition	2.40 ± 1.98 years	1.55 ± 1.80 years
	Proficiency	9.40 ± 0.68	8.08 ± 1.33
	Exposure	62.75 ± 12.62	37.25 ± 12.62

*Proficiency is rated on a scale from 0 (none) to 10 (perfect) and reports of exposure within an individual sum to 100% across the two languages.*

### Inhibitory Control Task

Inhibitory control was assessed by the Integrated Visual and Auditory Continuous Performance Test (IVA + Plus^1^, Richmond, VA, United States). This test is 20 min and is administered via a laptop computer. During this test 500 trials of 1’s and 2’s are visually or auditorily presented in a pseudo-random order. The participant clicks the mouse when a 1 (but not a 2) is seen or heard. Thus, the participant must attend to the number while the modality is not a guiding cue to completing this task. Responses were converted to age-normed standard scores. These scores reflect how well the participant adapted to a change in modality when responding to 1’s and ignoring 2’s during the test. That is, a higher standard score reflects a smaller reaction time difference between modality switch and non-switch trials.

### Competing-Talkers Selective Attention Task

#### Overview

Participants completed a selective attention task in which they listened to a target sentence presented simultaneously with a competing sentence. Modeled after the Coordinate Response Measure Corpus (Bolia et al., 2000), all sentences were of the format ‘Ready [call sign] go to [color] [number] now.’ Every trial consisted of two sentences, spoken simultaneously. One of the sentences was spoken by a female and one of the sentences was spoken by a male. One sentence had the call sign ‘baron’ and the other sentence had the call sign ‘tiger.’ The participant was assigned one of these call signs and was instructed to listen to the sentence that contained the target call sign. There was equal probability that the target call sign would be spoken by the male or female on any given trial. For the duration of a trial, four color-number combinations (e.g., red 3), arranged in the shape of an isosceles trapezoid, were projected on a screen in front of the participant. The four color-number options for each trial were (1) the target combination, (2) the competing color and the competing number, (3) the target color with the competing number, and (4) the competing color with the target number. At the end of the trial (i.e., after ‘now,’ during a 500 ms interstimulus interval) the participant selected a button that corresponded to the color-number combination that (s)he perceived using a hand-held response box with four buttons arranged in the same trapezoidal pattern. Evoked brain responses to the mixed sentences were collected to simultaneously measure online cortical and subcortical auditory processing during this selective attention task. Following the task, the participant’s brain responses to the mixed sentences were recorded under a passive listening condition while the subject watched a muted cartoon.

#### Stimuli

**FIGURE 1:**
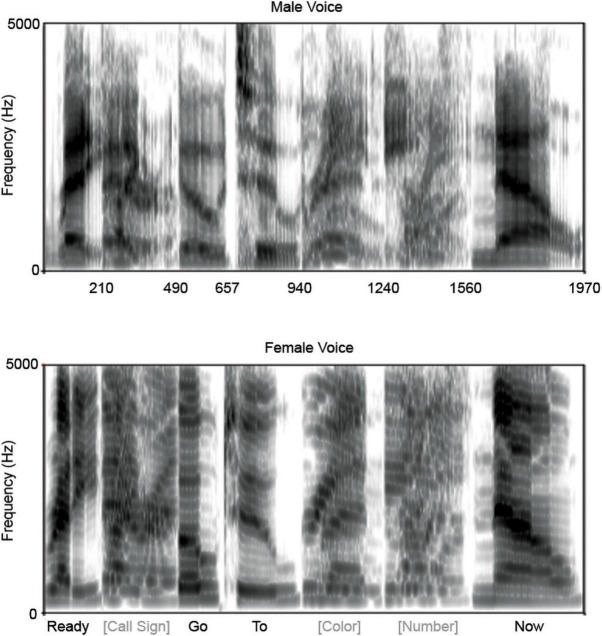
Spectrograms of stimuli. For each voice, male **(top)** and female **(bottom)**, only one token of ‘ready,’ ‘go,’ ‘to,’ and ‘now’ were used, while two call signs, three colors, and multiple numbers were used. The segments of the spectrograms corresponding to the latter three are averages across the multiple utterances. The total utterance duration was 1,970 ms. Time labels on the *x*-axis of the male voice correspond to the word boundaries (e.g., 210 ms is when ‘ready’ ends and the call signs begin).

To maximize differences in the spectral components of the competing sentences, sentences were constructed using natural utterances recorded at 44.1 kHz spoken by a female (average F0 = 220 Hz) and a male (average F0 = 137 Hz). For both the female and male sentences, a single exemplar of ‘ready,’ ‘go,’ ‘to’ and ‘now’ were used and 48 combinations of call sign (baron or tiger), color (red, blue, or green) and monosyllabic number (1, 2, 3, 4, 5, 6, 8, 9) were generated. Overlapping utterances (e.g., ‘ready’) were duration normalized in Audacity (Audacity 1.3.13^2^) between the two speakers and in the case of multiple possibilities (i.e., the call signs and the numbers) all potential utterances were duration normalized. This normalization ensured that all sentences would be of the same duration and that words would occur at the same time on every trial. To shorten collection time, utterances were compressed by 35% in Audacity (without altering the pitch). Words were root-mean-square normalized to 70 dB SPL, so that the signal-to-noise ratio was essentially 0 over the duration of each utterance. These individual utterances were then concatenated to form female and male versions of the 48 possible sentence combinations, each with a duration of 1970 ms (Figure 1). A sentence spoken by the female was then combined with a sentence spoken by the male and the mixed sentences were pseudo-randomly arranged for presentation with the caveats that no mixed sentence combination would be presented twice in succession, and that on any given trial the male and female were saying different colors and numbers. The same presentation order of these mixed sentences was used across all participants.

For analyses, the pitch contours of an average of the male sentences and, separately, an average of the female sentences were extracted in Praat^3^ with the autocorrelation method using a silence threshold of 0.0003, a voicing threshold of 0.15, an octave cost of 0.01, an octave jump cost of 0.35, and a voiced/unvoiced cost of 0.04. These parameters were chosen to maximize the chances of identifying a continuous pitch contour for both voices.

#### Experimental Design

##### Setup

The participant sat in a comfortable chair in a sound-proof booth. Seven Ag/AgCl electrodes were affixed to the participant’s scalp: active at central midline (Cz), frontal midline (Fz), and parietal midline (Pz), reference at the right and left earlobes, low forehead as ground, and a vertical eye channel placed below the left eye. Contact impedance was kept below 5 kΩ with interelectrode impedance differences < 3 kΩ. The participant was given a response box with four buttons that spatially matched the layout of the four possible color-number combinations projected on a screen in front of the participant on each trial. The mixed sentences were presented in alternating polarity at a rate of 0.4/s to both ears through insert earphones to the participant via NeuroSCAN’s STIM2 presentation software (GenTask module, Compumedics, Inc.) at 70 dB SPL. Participants’ behavioral and brain responses on each trial were recorded in SCAN 4.5 (Compumedics, Inc.) in continuous acquisition using an analog-to-digital conversion rate of 10 kHz and online filter settings of DC to 2000 Hz.

##### Training

After the electrodes were applied to the participant and instructions were given, testing began with training to familiarize the participant with the selective attention task. Training consisted of 12 blocks of 10 trials, where each trial is a mixed sentence (i.e., one sentence spoken by a male, giving instructions for one call sign, and one spoken by a female, giving instructions for the other call sign). For each block, the participant was instructed to attend to one call sign. At the end of the sentence, the participant pressed a button on the response box that corresponded to the color-number combination that (s)he was instructed to go to on that trial. Attended call sign alternated between blocks (e.g., ‘Tiger’ for block 1, ‘Baron’ for block 2, etc.). In any one trial, the attend call sign (e.g., ‘Tiger’) could be spoken by either the male or the female. Participants had to score 70% (i.e., 7/10) correct on two consecutive practice blocks or complete all 12 practice blocks to move on to the active task. Monolinguals and bilinguals did not differ on the number of lists required to achieve a passing score [monolinguals: 8.00 ± 3.74; bilinguals: 9.95 ± 3.39; *F*(1,38) = 2.980, *p* = 0.092, η_p_^2^ = 0.073].

##### Active Condition: Selective Attention to Competing Talkers

The active condition was identical to the training except that the active condition consisted of four blocks, with 500 trials comprising each block. During each block, the participant attended to one call sign (‘Tiger’ on blocks 1 and 3, ‘Baron’ on blocks 2 and 4). The male and female talkers each spoke the target sentence (i.e., the sentence containing the target call sign) 50% of the time during a block, resulting in 1000 trials where the male was attended and 1000 in which the female was attended across the entire active listening condition. Including breaks, this condition lasted about 90 min.

##### Passive Condition: Cartoon Watching While Hearing Competing Talkers

After the active task was completed, participant brain responses were collected during a passive listening condition. During this condition, participants were told that they no longer needed to pay attention to the competing talkers. Instead, they were instructed to watch a muted cartoon (‘Road Runner and Friends’ from *Looney Tunes Golden Collection Volume II*). Passive responses were recorded to 600 mixed sentences. These 600 mixed sentences were the same as the initial 600 sentences presented during the active listening condition. This condition lasted about 30 min.

#### Data Reduction and Processing

Offline, the triggers on both the active and passive continuous files were re-coded to reflect participant performance on the active task (i.e., correct male attend, correct female attend, incorrect male attend, and incorrect female attend). Analyses were run on correctly attended trials to ensure that the analyzed trials were ones in which the participant was actively engaging with the stimuli during the selective attention task. To include all participants, only the first 175 correct trials were used (i.e., the lowest number available after factoring in task performance and artifact rejection of the subcortical responses as described below). Intertrial phase consistency of the brain response was analyzed using two different sets of parameters that reflected primarily low-frequency activity from the cortex and separately high-frequency activity from the auditory midbrain.

##### Cortical

To assess cortical neural entrainment, intertrial phase consistency over discrete frequency bands was calculated for active versus passive listening. First, data were downsampled to 500 Hz and spatial filtering was performed using singular value decomposition in Neuroscan Edit v4.3 to remove eyeblinks. Next, phase consistency calculations were performed over consecutive 200 ms sliding response windows with a 100 ms on and off hanning ramp (199 ms overlap) over the duration of the response, which provided us with 1-ms resolution of phase consistency. In each 200 ms window, a fast Fourier transform was used to calculate the spectrum of each trial. This calculation resulted in a vector for each frequency that contained a vector length, a reflection of encoding strength for each frequency, and a phase, which contained information about the timing of the response to that frequency. To examine the phase consistency of the response, each vector was transformed into a unit vector (i.e., amplitude information was removed) and then the first 175 vectors (i.e., trials) at each frequency were averaged so that the length of the resulting vector provided a measure of the intertrial phase consistency. Active versus passive comparisons were done on a composite of the attend male correct and attend female correct responses. Phase consistency values were then computed for theta (3–7 Hz), alpha (8–12 Hz), beta (20–30 Hz), gamma (31–50 Hz), and high gamma (65–150 Hz) frequency bands during the individual words in the sentence. The amplitude envelopes of the sentences have the highest energy at 4 Hz, which is within the theta band.

##### Subcortical

To assess subcortical neural entrainment, intertrial phase consistency to the pitch contour of the male voice and the pitch contour of the female voice were calculated on a composite of the attend male correct and attend female correct responses. These phase-consistency calculations were performed over a consecutive 40 ms sliding response window with 20 ms on and off hanning ramp (39 ms overlap) for the duration of the response, providing us with 1-ms resolution of phase consistency. Only responses that fell below the artifact rejection criterion (±50 μV) were included in the analyses. Phase-consistency calculations for subcortical frequencies were identical to calculation procedures for cortical frequencies. Phase-consistency values were computed for the frequencies (±2 Hz) comprising the pitch contour of the male voice, and separately the female voice. Phase consistency for each voice were calculated, using a 10-ms lag, for words that were consistent on every trial (‘ready,’ ‘go,’ ‘to,’ and ‘now’). Using a 10 ms lag ensures that we are picking up on subcortical encoding of the pitch contour, given that this lag corresponds to the lag between stimulus and subcortical response that has been reported previously (Chandrasekaran and Kraus, 2010; Coffey et al., 2016). Parts of the sentence that contained multiple word options (e.g., number) were not analyzed because there was no consistent pitch contour to track across the multiple words (Figure 1).

### Data Analyses

#### Behavioral Performance

Bilingual and monolingual groups were compared on their performance on the selective attention task and the inhibitory control test using a separate univariate analysis of variance (ANOVA) for each test. Additionally, performance on these behavioral tests was correlated within each language group to determine whether the participants relied upon their inhibitory control abilities to perform the selective attention task. Mean ±1 standard deviation are reported for the two language groups on each measure.

#### Cortical Phase Consistency

Cortical responses were compared using a 2 (language group: Monolingual, Bilingual) × 2 (listening condition: Active, Passive) × 3 (electrode: Fz, Cz, Pz) × 7 (word: ‘ready,’ [call-sign], ‘go,’ ‘to,’ [color], [number], ‘now’) × 5 (frequency band: theta, alpha, beta, gamma, and high gamma) repeated measures analysis of variance (RMANOVA) to determine if cortical phase consistency differed between language groups for the active and passive listening conditions. This RMANOVA was followed up with a 2 (language group: Monolingual, Bilingual) × 2 (listening condition: Active, Passive) × 3 (Electrode: Fz, Cz, Pz) × 7 (Word: ‘ready’, [call-sign], ‘go,’ ‘to,’ [color], [number], ‘now’) RMANOVA for each frequency band. Significant interactions were further analyzed to characterize the effects. *Post hoc* analyses were corrected for multiple comparisons. Mean ± 1 standard deviation for the various measures are reported within the text in parentheses. Remaining tests are reported in the Supplementary Material.

#### Subcortical Phase Consistency

To identify differences in subcortical phase consistency as a function of language experience, subcortical phase consistency to the F0 was compared between active and passive listening conditions using a 2 (language group: Monolingual, Bilingual) × 2 (condition: Active, Passive) × 3 (electrode: Fz, Cz, Pz) × 2 (pitch contour: Male, Female) × 4 (word: ‘ready,’ ‘go,’ ‘to,’ ‘now’) RMANOVA. Significant interactions were analyzed further to characterize the effects. *Post hoc* analyses were corrected for multiple comparisons. Mean ± 1 standard deviation for the various measures are reported within the text in parentheses. Remaining tests are reported in the Supplementary Material.

#### Cortical – Subcortical Comparisons

##### Effects of Language Experience

To further investigate whether bilinguals and monolinguals engage different mechanisms for active and passive listening, we explored whether cortical and subcortical interactions during active and passive listening differed for the two groups. To do this, we averaged all active, and separately passive, cortical phase consistency data, collapsing across electrode, frequency band, and word for monolinguals and bilinguals. Subcortical active and passive phase consistency data was similarly averaged over electrode, pitch contour (i.e., talker), and word. To facilitate comparison between cortical and subcortical phase consistency, only the four words with consistent pitch contours across trials (‘ready,’ ‘go,’ ‘to,’ ‘now’) were included in these calculations. These composite values were then analyzed using a 2 (language group: Monolingual, Bilingual) × 2 (auditory region: Cortical, Subcortical) RMANOVA to determine whether there were differences in the way monolinguals and bilinguals utilized cortical and subcortical auditory processing when listening under different conditions.

##### Disentangling High Gamma and the Male Pitch Contour

High gamma (65 – 150 Hz) and phase consistency to the male pitch contour (average 137 Hz) overlap in frequency but are presumed to originate from cortical and subcortical sources, respectively (Edwards et al., 2005; Chandrasekaran and Kraus, 2010; Mesgarani and Chang, 2012; Bidelman, 2015; White-Schwoch et al., 2019). To determine whether we were capturing two distinct sources of activity, these data were analyzed using a 2 (response: high gamma, male pitch contour) × 2 (listening condition: Active, Passive) × 3 (electrode: Fz, Cz, Pz), by 4 (word: ‘ready,’ ‘go,’ ‘to,’ ‘now’) RMANOVA. To illustrate differences between cortical and subcortical consistency, consistency of the male pitch contour, high gamma, and the female pitch contour were plotted by word and listening condition, to visually depict the similarities and differences of the male pitch contour with respect to high gamma activity, which has known cortical generators (Edwards et al., 2005; Cervenka et al., 2011; Mesgarani and Chang, 2012), and the female pitch contour, whose frequency of 220 Hz is beyond cortical phase-locking abilities and thus reflects predominantly midbrain sources (Liang-Fa et al., 2006; Coffey et al., 2016, 2019; White-Schwoch et al., 2019).

## Results

In summary, monolinguals and bilinguals perform equivalently on the selective attention and inhibitory control tasks, but a relationship between performance on the two tasks exists only in bilinguals. Also, cortical consistency is enhanced in monolinguals, relative to bilinguals, especially during active listening. In contrast, subcortical consistency is enhanced in bilinguals relative to monolinguals, but is reduced during active relative to passive listening.

### Behavioral

**FIGURE 2:**
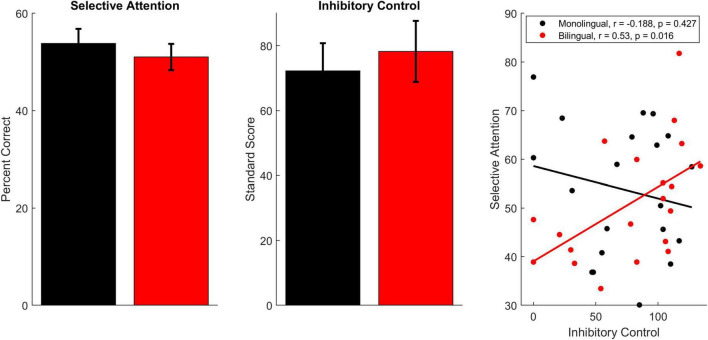
Behavioral performance on the selective attention and inhibitory control tests. Bilinguals (red) and monolinguals (black) performed equivalently on both of these measures. However, performance on these measures was only correlated in bilingual participants. Error bars display standard error of the mean.

Monolingual and bilingual participants performed equivalently on the selective attention task [Figure 2, monolinguals: 53.80 ± 13.47%; bilinguals: 51.01 ± 12.13%; *F*(1,38) = 0.47, *p* = 0.497, η_p_^2^ = 0.012] as well as the inhibitory control test [monolinguals: 72.25 ± 38.13; bilinguals: 78.25 ± 42.00; *F*(1,38) = 0.224, *p* = 0.639, η_p_^2^ = 0.006]. While performance on these tests did not relate in monolinguals [*r*(18) = –0.188, *p* = 0.427], performance was related for bilinguals [*r*(18) = 0.530, *p* = 0.016], with better inhibitory control corresponding to better performance on the selective attention task. The difference between the correlation for bilinguals and the correlation for monolinguals was significant (*z* = –2.275, *p* = 0.011).

### Cortical Phase Consistency

**FIGURE 3:**
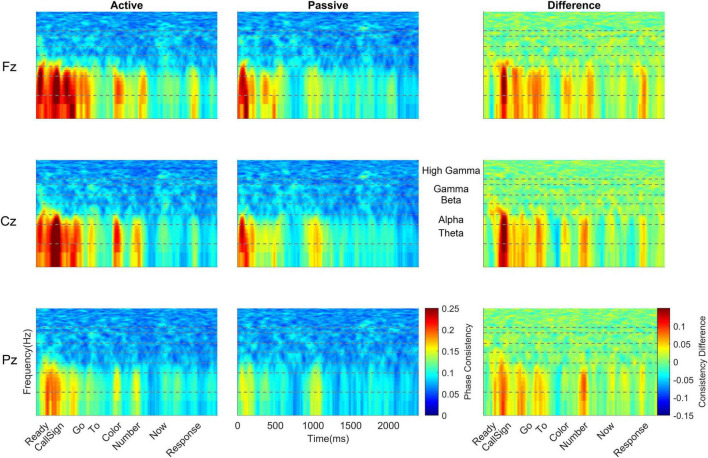
Monolingual cortical phase consistency. Monolingual phase consistency is plotted at Fz **(top)**, Cz **(middle)**, and Pz **(bottom)** for active (left) and passive (center) listening conditions. For these six plots, color represents greater phase consistency, with warmer colors indicating greater consistency and cooler colors representing little to no consistency. The rightmost plots show the difference in phase consistency between active and passive listening conditions, with warmer colors indicating greater consistency during active listening and cooler colors indicating more consistency during passive listening. Monolinguals showed the most cortical consistency during active listening (left column), with the beginning of the sentence showing the highest consistency that declined as the sentence unfolded. This effect was varied across the scalp, being greatest at Fz and lowest at Pz. Active and difference plots align the cortical consistency with the words in the sentence and passive plots provide an indication of time. Dashed lines on all plots identify the borders of the frequency bands, which are labeled between the passive and difference plots for Cz. To visualize the lower frequencies, all *y*-axes are plotted on a log scale.

**FIGURE 4:**
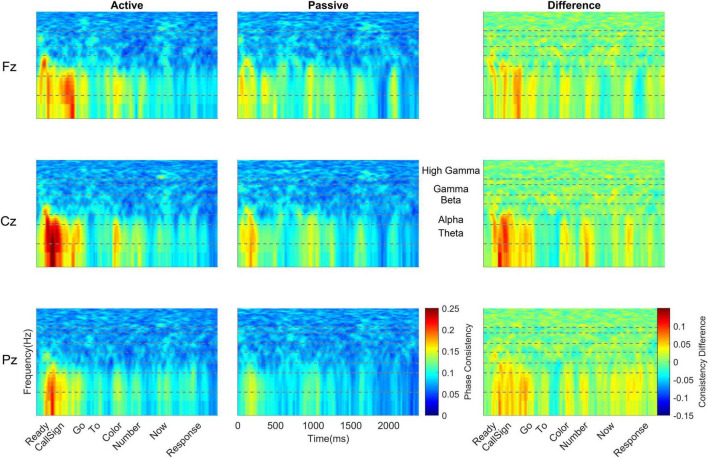
Bilingual cortical phase consistency. Bilingual phase consistency is plotted at Fz **(top)**, Cz **(middle)**, and Pz **(bottom)** for active (left) and passive (center) listening conditions. For these six plots, color represents greater phase consistency, with warmer colors indicating greater consistency and cooler colors representing little to no consistency. The rightmost plots show the difference in phase consistency between active and passive listening conditions, with warmer colors indicating greater consistency during active listening and cooler colors indicating more consistency during passive listening. Compared to monolinguals, bilinguals showed a smaller change in cortical consistency from active to passive listening, driven by a smaller increase in consistency during active listening. However, like monolinguals, bilinguals still had greater cortical consistency during active listening (left column), with consistency highest at the beginning of the sentence and declining as the sentence unfolded. For bilinguals, this effect was greatest at Cz; it did not show the same rostral to caudal distribution seen in monolinguals. Active and difference plots align the cortical consistency with the words in the sentence and passive plots provide an indication of time. Dashed lines on all plots identify the borders of the frequency bands, which are labeled between the passive and difference plots for Cz. To visualize the lower frequencies, all *y*-axes are plotted on a log scale.

Across all cortical frequency bands, there was a large effect of listening condition [*F*(1,38) = 33.26, *p* < 0.0005, η_p_^2^ = 0.467, see Table 2 for all main effects and interactions from this RMANOVA], with active yielding higher cortical phase consistency than passive (active: 0.098 ± 0.011, passive: 0.089 ± 0.012; Figures 3, 4). Notably, the effect of language group was significant [*F*(1,38) = 4.646, *p* = 0.038, η_p_^2^ = 0.109]; monolinguals had higher cortical consistency than bilinguals (monolinguals: 0.097 ± 0.011, bilinguals: 0.090 ± 0.009, Figures 3, 4, 7). There were also main effects of electrode [*F*(2,76) = 55.82, *p* < 0.0005, η_p_^2^ = 0.595], word [*F*(6,228) = 42.63, *p* < 0.0005, η_p_^2^ = 0.529], and frequency band [*F*(4,152) = 206.12, *p* < 0.0005, η_p_^2^ = 0.844].

**TABLE 2:** Cortical main effects and interactions for 2 (language group: monolingual, bilingual) × 2 (listening condition: active, passive) × 3 (electrode: Fz, Cz, Pz) × 7 (word: ‘ready,’ [call-sign], ‘go,’ ‘to,’ [color], [number], ‘now’) × 5[frequency band: theta (3–7 Hz), alpha (8–12 Hz), beta (20–30 Hz), gamma (31–50 Hz), and high gamma (65–150 Hz)] RMANOVA.

	*F*	df	*p*	η_p_^2^
**Listening condition**	**33.26**	**(1, 38)**	**<0.0005**	**0.467**
**Language group**	**4.65**	**(1, 38)**	**0.038**	**0.109**
**Electrode**	**55.82**	**(2, 76)**	**<0.0005**	**0.595**
**Word**	**42.63**	**(6, 228)**	**<0.0005**	**0.529**
**Frequency band**	**206.12**	**(4, 152)**	**<0.0005**	**0.844**
Listening condition × Language group	1.48	(1, 38)	0.232	0.037
*Listening condition × Electrode*	*2.55*	*(2, 76)*	*0.085*	*0.063*
**Listening condition × Word**	**5.69**	**(6, 228)**	**<0.0005**	**0.130**
**Listening condition × Frequency band**	**31.12**	**(4, 152)**	**<0.0005**	**0.450**
Electrode × Language group	1.73	(2, 76)	0.183	0.044
**Electrode × Word**	**5.42**	**(12, 456)**	**<0.0005**	**0.125**
**Electrode × Frequency band**	**29.40**	**(8, 304)**	**<0.0005**	**0.436**
Word × Language group	1.10	(6, 228)	0.366	0.028
**Word × Frequency Band**	**21.78**	**(24, 912)**	**<0.0005**	**0.364**
**Frequency band × Language group**	**5.21**	**(4, 152)**	**0.001**	**0.121**
Listening condition × Electrode × Language group	2.05	(2, 76)	0.135	0.051
Listening condition × Word × Language group	0.70	(6, 228)	0.646	0.018
Listening condition × Frequency band × Language group	1.83	(4, 152)	0.126	0.046
Listening condition × Electrode × Word	1.04	(12, 456)	0.412	0.027
**Listening condition × Electrode × Frequency band**	**2.23**	**(8, 304)**	**0.025**	**0.056**
**Listening condition × Word × Frequency band**	**6.72**	**(24, 912)**	**<0.0005**	**0.150**
Electrode × Word × Language group	1.01	(12, 456)	0.443	0.026
**Electrode × Frequency band × Language group**	**2.74**	**(8, 304)**	**0.006**	**0.067**
**Electrode × Word × Frequency band**	**9.98**	**(48, 1824)**	**<0.0005**	**0.208**
Word × Frequency band × Language group	0.87	(24, 912)	0.651	0.022
**Listening condition × Electrode × Word × Language group**	**1.93**	**(12, 456)**	**0.029**	**0.048**
Listening condition × Electrode × Frequency band × Language group	0.78	(8, 304)	0.621	0.020
**Listening condition × Electrode × Word × Frequency band**	**6.54**	**(48, 1824)**	**<0.0005**	**0.147**
Listening condition × Word × Frequency band × Language group	0.93	(24, 912)	0.562	0.024
Electrode × Word × Frequency band × Language group	0.68	(48, 1824)	0.954	0.018
Listening condition × Electrode × Word × Frequency band × Language group	0.80	(48, 1824)	0.832	0.021

*Significant main effects and interactions are bolded and trending differences are indicated by italics.*

With respect to the electrode main effect, Pz (0.0859 ± 0.008) had lower cortical consistency than either Fz [0.098 ± 0.014, *t*(39) = 10.126, *p* < 0.0005, *d* = 1.599] or Cz [0.097 ± 0.012, *t*(39) = 7.651, *p* < 0.0005, *d* = 1.214], while Cz and Fz did not differ [*t*(39) = 0.945, *p* = 0.350, *d* = 0.159].

For the main effect of word, phase consistency was highest at ‘ready’ (0.113 ± 0.023), followed by the call sign (0.101 ± 0.014), ‘Go’ (0.059 ± 0.017), the color (0.093 ± 0.015), ‘to’ (0.086 ± 0.010), the number (0.082 ± 0.012), and the lowest consistency was over ‘now’ (0.079 ± 0.007). The higher consistency for ‘ready’ was significant compared to each of the remaining six words in the sentence (all *t*’s 4.205 – 10.785, all *p*’s < 0.0005, all *d*’s 0.664 – 1.706), while the call sign also had significantly higher consistency than ‘to,’ ‘color,’ the number, and ‘now’ (all *t*’s 3.389 – 10.583, all *p*’s ≤ 0.0016, all *d*’s 0.537 – 1.670), ‘go’ had higher cortical consistency than ‘to’ [*t*(39) = 4.237, *p* < 0.0005, *d* = 0.669], the number [*t*(39) = 6.416, *p* < 0.0005, *d* = 1.013] and ‘now’ [*t*(39) = 7.065, *p* < 0.0005, *d* = 1.109], ‘to’ had lower consistency than the color [*t*(39) = 3.640, *p* = 0.001, *d* = 0.577] but higher consistency relative to ‘now’ [*t*(39) = 4.165, *p* < 0.0005, *d* = 0.652], and the color had higher consistency than the number [*t*(39) = 5.364, *p* < 0.0005, *d* = 0.850] and ‘now’ [*t*(39) = 6.103, *p* < 0.0005, *d* = 0.962], while the remaining comparisons were not significant (all *t*’s ≤ 2.547, all *p*’s ≥ 0.015).

Considering the main effect of frequency band, as the frequency increased, the phase consistency decreased, such that the greatest consistency was seen over theta (0.141 ± 0.032), followed by alpha (0.118 ± 0.022), beta (0.078 ± 0.009), gamma (0.076 ± 0.007), and high gamma (0.071 ± 0.005). All pairwise frequency-band differences were significant (all *t*’s 5.584 – 14.170, all *p*’s < 0.0005, all d’s 0.888 – 2.241) except the difference in consistency between beta and gamma [*t*(39) = 1.673, *p* = 0.102, *d* = 0.257].

To further explore the interaction between listening condition and frequency band, RMANOVAs were run within each frequency band. Within the theta and alpha bands, there was a significant effect of listening condition [theta: *F*(1,38) = 42.81, *p* < 0.0005, η_p_^2^ = 0.530; alpha: *F*(1,38) = 23.65, *p* < 0.0005, η_p_^2^ = 0.384], while listening condition did not significantly influence phase consistency in the beta, gamma, and high gamma bands (see Table 3 for all statistics from this analysis). Similar to the overall effect described above, theta and alpha consistency increased during active (theta: 0.145 ± 0.030; alpha: 0.119 ± 0.021), relative to passive (theta: 0.119 ± 0.030; alpha: 0.104 ± 0.021), listening. Also within the theta and alpha bands, there was a significant effect of language group [theta: *F*(1,38) = 5.11, *p* = 0.03, η_p_^2^ = 0.119; alpha: *F*(1,38) = 5.58, *p* = 0.023, η_p_^2^ = 0.128]. Consistent with the effect described above, monolinguals (theta: 0.141 ± 0.031; alpha: 0.119 ± 0.020) had greater cortical consistency than bilinguals (theta: 0.123 ± 0.019; alpha: 0.105 ± 0.017) over both of these frequency bands (Figures 3, 4). These effects were mirrored in the frequency band by language group interaction, electrode by frequency band by language group interaction, and listening condition, by electrode, by word, by language group interaction, which all demonstrated greater theta and alpha activity for monolinguals relative to bilinguals that was greatest during active listening over Fz and Cz electrodes over the call sign, the words ‘go’ and ‘to’ and the number (see Supplementary Material results for additional figures and statistics for these analyses and all remaining cortical analyses).

**TABLE 3:** Cortical analyses within individual frequency bands.

		Theta			Alpha			Beta			Gamma			High Gamma		
	df	F	p	η_p_^2^	F	p	η_p_^2^	F	p	η_p_^2^	F	p	η_p_^2^	F	p	η_p_^2^
Listening condition	1, 38	**42.81**	**<0.0005**	**0.530**	**23.65**	**<0.0005**	**0.384**	2.76	0.105	0.068	0.001	0.976	0	0.92	0.345	0.024
Language group	1, 38	**5.11**	**0.030**	**0.119**	**5.58**	**0.023**	**0.128**	0.12	0.732	0.003	*3.17*	*0.083*	*0.077*	1.60	0.214	0.040
Electrode	2, 76	**35.58**	**<0.0005**	**0.484**	**56.79**	**<0.0005**	**0.599**	**11.07**	**<0.0005**	**0.226**	**22.63**	**<0.0005**	**0.373**	**5.34**	**<0.0005**	**0.123**
Word	6, 228	**29.08**	**<0.0005**	**0.434**	**35.70**	**<0.0005**	**0.484**	**10.53**	**<0.0005**	**0.217**	**17.19**	**<0.0005**	**0.311**	**14.09**	**<0.0005**	**0.270**
Listening condition × Language group	1, 38	1.16	0.288	0.030	*3.05*	*0.089*	*0.074*	0.66	0.420	0.017	0.19	0.669	0.005	0.03	0.863	0.001
Listening condition × Electrode	2, 76	1.21	0.305	0.031	**5.16**	**0.008**	**0.120**	1.06	0.351	0.027	0.03	0.966	0.001	2.36	0.102	0.058
Listening condition × Word	6, 228	**5.91**	**<0.0005**	**0.135**	**11.75**	**<0.0005**	**0.219**	**4.28**	**<0.0005**	**0.101**	0.26	0.957	0.007	0.39	0.888	0.010
Electrode × Language Group	2, 76	*2.93*	*0.059*	*0.072*	1.59	0.211	0.040	0.91	0.406	0.023	2.05	0.135	0.051	0.09	0.917	0.002
Electrode × Word	12, 456	**3.11**	**<0.0005**	**0.076**	**6.20**	**<0.0005**	**0.140**	**2.20**	**0.011**	**0.055**	*1.64*	*0.077*	*0.041*	*1.68*	*0.069*	*0.042*
Word × Language group	6, 228	0.99	0.436	0.025	1.06	0.387	0.027	0.20	0.977	0.005	0.46	0.840	0.012	*2.09*	*0.056*	*0.052*
Listening condition × Electrode × Language group	2, 76	*2.39*	*0.099*	*0.059*	1.90	0.157	0.048	0.12	0.885	0.003	0.12	0.889	0.003	0.14	0.866	0.004
Listening condition × Word × Language group	6, 228	0.91	0.492	0.023	0.61	0.721	0.016	0.77	0.592	0.020	0.28	0.971	0.006	0.58	0.743	0.015
Listening condition × Electrode × Word	12, 456	**1.84**	**0.040**	**0.046**	1.39	0.168	0.035	0.82	0.632	0.021	1.38	0.172	0.035	0.68	0.776	0.017
Electrode × Word × Language group	12, 456	1.09	0.363	0.028	0.66	0.793	0.017	0.65	0.795	0.017	*1.63*	*0.081*	*0.041*	1.28	0.227	0.033
Listening condition × Electrode × Word × Language group	12, 456	0.75	0.632	0.019	0.78	0.671	0.020	0.87	0.580	0.022	**1.93**	**0.029**	**0.048**	*1.60*	*0.088*	*0.040*

*Significant main effects and interactions are bolded and trending differences are indicated by italics.*

### Subcortical Phase Consistency

**FIGURE 5:**
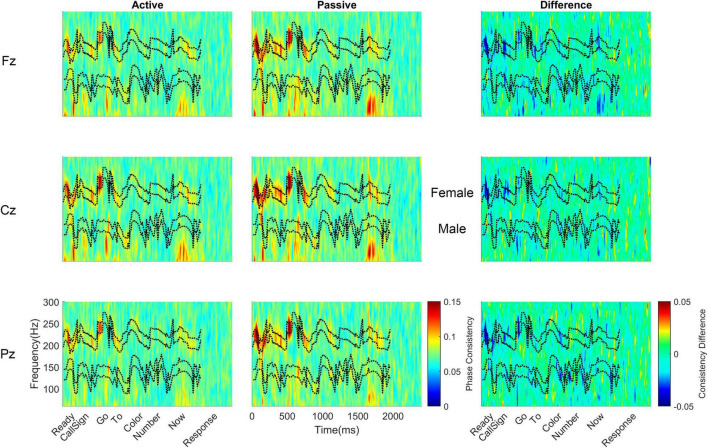
Monolingual subcortical phase consistency. Monolingual phase consistency is plotted at Fz **(top)**, Cz **(middle)**, and Pz **(bottom)** for active (left) and passive (center) listening conditions. For these six plots, color represents greater phase consistency, with warmer colors indicating greater consistency and cooler colors representing little to no consistency. The rightmost plots show the difference in phase consistency between active and passive listening conditions, with warmer colors indicating greater consistency during active listening and cooler colors indicating more consistency during passive listening. The top two black dashed lines in each plot indicate the female pitch contour ± 10 Hz and the bottom two dashed lines indicate the male pitch contour ± 10 Hz (labeled between the passive and difference plots for Cz). Note that the regions that have multiple words (e.g., number) have no phase consistency while phase consistency is evident over the words that are identical across trials (i.e., ‘ready,’ ‘go,’ ‘to,’ and ‘now’). Monolinguals’ subcortical phase consistency decreased during active listening, in contrast to the effects of active listening on cortical phase consistency.

**FIGURE 6:**
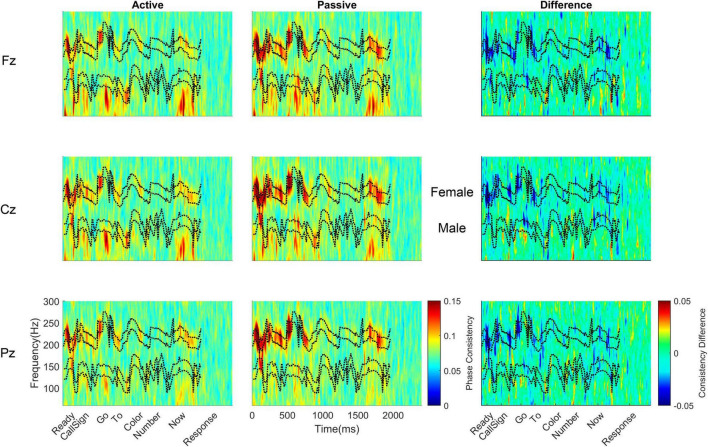
Bilingual subcortical phase consistency. Bilingual phase consistency is plotted at Fz **(top)**, Cz **(middle)**, and Pz **(bottom)** for active (left) and passive (center) listening conditions. For these six plots, color represents greater phase consistency, with warmer colors indicating greater consistency and cooler colors representing little to no consistency. The rightmost plots show the difference in phase consistency between active and passive listening conditions, with warmer colors indicating greater consistency during active listening and cooler colors indicating more consistency during passive listening. The top two black dashed lines in each plot indicate the female pitch contour ± 10 Hz and the bottom two dashed lines indicate the male pitch contour ± 10 Hz (labeled between the passive and difference plots for Cz). Note that the regions that have multiple words (e.g., number) have no phase-locking while phase-locking consistency is evident over the words that are identical across trials (i.e., ‘ready,’ ‘go,’ ‘to,’ and ‘now’). Similar to monolinguals, active listening led to a decline in phase consistency in the subcortical response. However, across both passive and active listening conditions, bilinguals had higher subcortical phase consistency than monolinguals.

**FIGURE 7:**
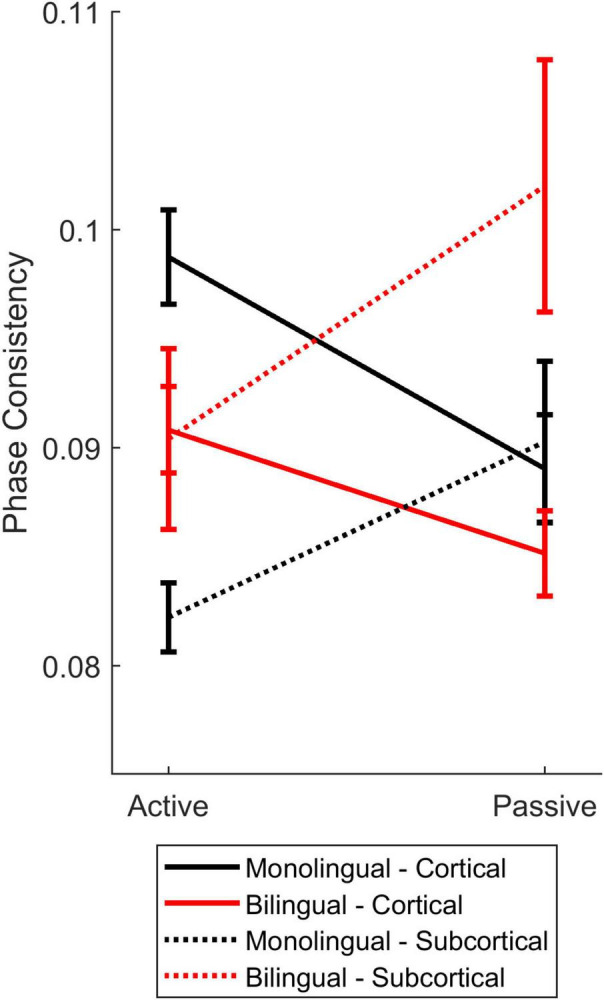
Cortical-subcortical comparisons in bilinguals and monolinguals. Differences in levels of cortical (solid lines) and subcortical (dashed lines) consistency for monolinguals (black) and bilinguals (red) during active (left) and passive (right) listening.

Active versus passive listening also led to differences in subcortical phase consistency [*F*(1,38) = 5.60, *p* = 0.023, η_p_^2^ = 0.128; Table 4]. However, the effects were in the opposite direction of the changes observed cortically. Whereas *cortical* consistency *increased* during active listening, *subcortical* phase consistency *decreased* during active listening (active: 0.086 ± 0.014; passive: 0.096 ± 0.022; Figures 5–7).

**TABLE 4:** Subcortical main effects and interactions for a 2 (language group: monolingual, bilingual) × 2 (listening condition: active, passive) × 3 (electrode: Fz, Cz, Pz) × 2 (pitch contour: male talker, female talker) × 4 (word: ‘ready,’ ‘go,’ ‘to,’ ‘now’) RMANOVA.

	*F*	df	*p*	η_p_^2^
**Listening condition**	**5.60**	**(1, 38)**	**0.023**	**0.128**
**Language group**	**6.15**	**(1, 38)**	**0.018**	**0.139**
**Pitch contour**	**80.48**	**(1, 38)**	**<0.0005**	**0.679**
**Electrode**	**24.70**	**(2, 76)**	**<0.0005**	**0.394**
**Word**	**59.18**	**(3, 114)**	**<0.0005**	**0.609**
Listening condition × Language group	0.18	(1, 38)	0.671	0.005
Pitch contour × Language group	0.29	(1, 38)	0.592	0.008
Electrode × Language group	0.47	(2, 76)	0.629	0.012
**Word × Language group**	**3.28**	**(3, 114)**	**0.024**	**0.079**
**Listening condition × Pitch contour**	**10.30**	**(1, 38)**	**0.003**	**0.213**
Listening condition × Electrode	0.01	(2, 76)	0.987	0
**Listening condition × Word**	**5.01**	**(3, 114)**	**0.003**	**0.117**
**Pitch contour × Electrode**	**17.29**	**(2, 76)**	**<0.0005**	**0.313**
**Pitch contour × Word**	**38.25**	**(3, 114)**	**<0.0005**	**0.502**
**Word × Electrode**	**4.11**	**(6, 228)**	**0.001**	**0.098**
Listening condition × Pitch contour × Language group	1.01	(1, 38)	0.321	0.026
Listening condition × Word × Language group	0.82	(3, 114)	0.486	0.021
**Listening condition × Pitch contour × Word**	**5.13**	**(3, 114)**	**0.002**	**0.119**
Listening condition × Electrode × Language group	2.24	(2, 76)	0.114	0.056
Listening condition × Pitch contour × Electrode	0.44	(2, 76)	0.645	0.011
Listening condition × Word × Electrode	0.17	(6, 228)	0.984	0.005
Pitch contour × Word × Language group	1.98	(3, 114)	0.121	0.050
Pitch contour × Electrode × Language group	1.90	(2, 76)	0.156	0.048
**Pitch contour × Word × Electrode**	**3.09**	**(6, 228)**	**0.006**	**0.075**
Word × Electrode × Language group	0.77	(6, 228)	0.595	0.020
Listening condition × Pitch contour × Word × Language group	0.29	(3, 114)	0.835	0.007
Listening condition × Pitch contour × Electrode × Language group	0.70	(2, 76)	0.498	0.018
Listening condition × Word × Electrode × Language group	0.91	(6, 228)	0.487	0.023
**Listening condition × Pitch contour × Word × Electrode**	**2.83**	**(6, 228)**	**0.011**	**0.069**
Pitch contour × Word × Electrode × Language group	1.09	(6, 228)	0.372	0.028
Listening condition × Pitch contour × Word × Electrode × Language group	0.67	(6, 228)	0.674	0.017

*Significant main effects and interactions are bolded.*

Subcortically, there was also a main effect of language group. Across both active and passive listening conditions, bilinguals (0.096 ± 0.016) had greater subcortical phase consistency than monolinguals [0.086 ± 0.008; *F*(1,38) = 6.147, *p* = 0.018, η_p_^2^ = 0.139; Table 4 and Figures 5–7].

In addition to the main effects of listening condition and language group, there were also main effects of pitch contour [i.e., male or female talker, *F*(1,38) = 80.476, *p* ≥ 0.0005, η_p_^2^ = 0.679], electrode [*F*(2,76) = 24.703, *p* ≥ 0.0005, η_p_^2^ = 0.394], and word [*F*(3,114) = 59.183, *p* ≥ 0.0005, η_p_^2^ = 0.609]. With respect to the pitch contour, there was higher subcortical consistency to the female pitch (0.100 ± 0.018) relative to the male pitch (0.082 ± 0.011). Interestingly, while Cz and Fz together showed the highest cortical consistency (see above), subcortical consistency was greatest only at Cz. Comparing the three electrodes, Cz (0.094 ± 0.014) had greater subcortical consistency than Fz [0.090 ± 0.013; *t*(39) = 6.306, *p* < 0.0005, *d* = 0.985] and Pz [0.090 ± 0.014; *t*(39) = 6.471, *p* < 0.0005, *d* = 1.020], while Fz and Pz did not differ [*t*(39) = 0.734, *p* = 0.467, *d* = 0.103]. For the words, all word pairs except for ‘to’ and ‘now’ [*t*(39) = 2.729, *p* = 0.009, *d* = 0.432] were significantly different. Specifically, ‘ready’ (0.109 ± 0.025) had greater consistency than ‘go’ [0.091 ± 0.013, *t*(39) = 6.062, *p* < 0.0005, *d* = 0.958], ‘to’ [0.085 ± 0.012, *t*(39) = 8.526, *p* < 0.0005, *d* = 1.347], and ‘now’ [0.081 ± 0.011, *t*(39) = 10.575, *p* < 0.0005, *d* = 1.671]; and ‘go’ had greater consistency than ‘to’ [*t*(39) = 2.561, *p* = 0.005, *d* = 0.468] and ‘now’ [*t*(39) = 5.767, *p* < 0.0005, *d* = 0.912].

In addition to these main effects, there were a number of interactions, whose results are in line with those detailed above and are described fully in the Supplementary Material. Briefly, we observed that the greatest differences between active and passive listening were in response to the female pitch contour and that these effects were largest earlier in the sentence, such that the greatest consistency was in response to the female ‘ready’ during passive listening.

### Cortical – Subcortical Comparisons

#### Effects of Language Experience

To understand how cortical and subcortical processing work in tandem during active and passive listening, and whether language experience influences the interaction between cortical and subcortical processing, we compared cortical and subcortical phase consistency across bilinguals and monolinguals. There were no main effects of listening condition [*F*(1,38) = 0.224, *p* = 0.639, η_p_^2^ = 0.006], auditory center {i.e., cortical vs. subcortical, [*F*(1,38) = 0.016, *p* = 0.901, η_p_^2^ = 0]}, or language group [*F*(1,38) = 0.621, *p* = 0.435, η_p_^2^ = 0.016]. Nor were there a listening condition by language group interaction (*F*(1,38) = 0.705, *p* = 0.406, η_p_^2^ = 0.018) or listening condition by auditory level by language group three-way interaction [*F*(1,38) = 0.004, *p* = 0.952, η_p_^2^ = 0). However, both the auditory center by language group [*F*(1,38) = 11.785, *p* = 0.001, η_p_^2^ = 0.237] and listening condition by auditory center [*F*(1,38) = 17.999, *p* < 0.0005, η_p_^2^ = 0.321] interactions were significant. These differences were driven by (1) greater cortical consistency for monolinguals, (2) greater subcortical consistency for bilinguals, (3) greater cortical consistency during active listening, and (4) greater subcortical consistency during passive listening. Interestingly, these effects resulted in matched levels of cortical and subcortical auditory consistency for bilinguals during *active* listening, caused by a reduction in subcortical consistency and an increase in cortical consistency (Figure 7, red lines). In contrast, monolinguals’ cortical and subcortical consistency were matched during *passive* listening, driven by a reduction in cortical consistency and an increase in subcortical consistency (Figure 7, black lines).

#### Comparing High Gamma and the Male Pitch Contour

**FIGURE 8:**
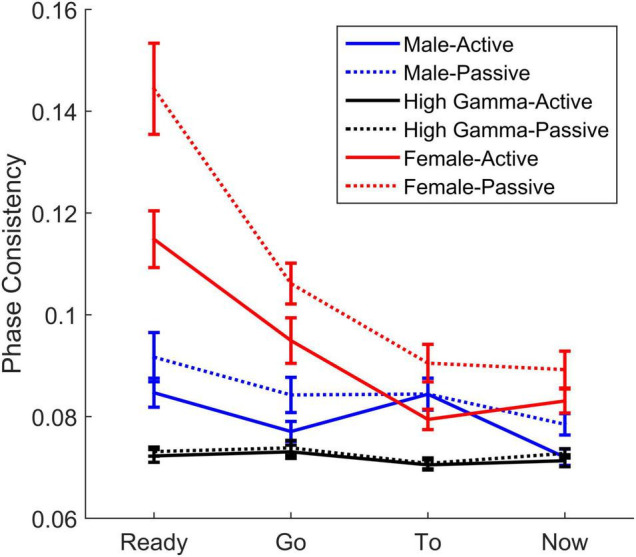
Comparisons of high gamma, male and female pitch contours across words and listening condition. While cortical high gamma phase consistency did not differ between conditions, both the subcortical phase consistency in response to the male and female pitch contours decreased during active listening.

To determine whether neural consistency over high gamma and in response to the male pitch contour reflected different sources, we analyzed these responses to determine if they were statistically different, if they were influenced differently by listening conditions, and if they showed different patterns across electrodes and/or words. We found that there was a difference between the responses and that they patterned differently across electrodes and words over the two listening conditions {main effect of response: *F*(1,39) = 46.48, *p* < 0.0005, η_p_^2^ = 0.544, response × listening condition × electrode × word interaction [*F*(6,234) = 2.22, *p* = 0.042, η_p_^2^ = 0.054, Figure 8 and see Table 5 for additional statistics]}. Specifically, high gamma consistency did not differ between the two listening conditions and, during both active and passive listening, was lower in consistency than the male pitch contour across the four words. In contrast, the male pitch contour showed an attention effect consistent with the effect seen for the female pitch contour: consistency to both contours increased during passive listening.

**TABLE 5:** RMANOVA comparisons of high gamma and male pitch contour consistency over active and passive listening conditions.

	*F*	df	*p*	η_p_^2^
**Response**	**46.48**	**(1, 39)**	**<0.0005**	**0.544**
**Listening condition**	**2.23**	**(1, 39)**	**0.143**	**0.054**
**Electrode**	**10.82**	**(2, 78)**	**<0.0005**	**0.217**
**Word**	**9.24**	**(3, 117)**	**<0.0005**	**0.192**
**Response × Electrode**	**3.39**	**(2, 78)**	**0.039**	**0.080**
**Response × Word**	**9.77**	**(3, 117)**	**<0.0005**	**0.200**
Listening condition × Response	2.35	(1, 39)	0.133	0.057
Listening condition × Electrode	0.50	(2, 78)	0.611	0.013
Listening condition × Word	1.52	(3, 117)	0.214	0.037
Electrode × Word	0.98	(6, 234)	0.437	0.025
Listening condition × Response × Word	1.70	(3, 117)	0.171	0.042
Electrode × Listening condition × Response	1.98	(2, 78)	0.145	0.048
Electrode × Listening condition × Word	0.47	(6, 234)	0.830	0.012
**Electrode × Response × Word**	**2.73**	**(6, 234)**	**0.014**	**0.065**
**Response × Listening condition × Electrode × Word**	**2.22**	**(6, 234)**	**0.042**	**0.054**

*Significant main effects and interactions are bolded.*

## Discussion

Bilinguals previously were shown to have enhanced inhibitory control (Bialystok, 2011, 2015) and subcortical encoding of the F0 of speech (Krizman et al., 2012; Skoe et al., 2017), processes that are fundamental to understanding speech in noise. Despite these advantages, bilinguals perform more poorly on clinical tests of speech-in-noise recognition (Shi, 2010, 2012; Lucks Mendel and Widner, 2016; Krizman et al., 2017; Skoe and Karayanidi, 2019). In this study, by assessing bilinguals and monolinguals on a selective attention task, a type of speech-in-noise task that calls upon auditory processing and executive control, we could determine whether bilingual cognitive and sensory enhancements have benefits for everyday listening situations. We find that although monolinguals and bilinguals performed similarly on the behavioral component of the selective attention task, the groups differed in the neural and cognitive processes engaged to perform this task. Specifically, bilinguals demonstrated enhanced subcortical phase-locking to the pitch contours of the talkers, while monolinguals had enhanced cortical phase consistency during active listening, particularly over the theta and alpha frequency bands. Additionally, a relationship between performance on the selective attention task and the inhibitory control test was seen only in bilinguals. Together, these results suggest that bilinguals utilize inhibitory control and enhanced subcortical auditory processing in real-world listening situations and are consistent with the hypothesis that bilingualism leads to mechanistic differences in how the brain engages with sound (Abutalebi et al., 2011; Ressel et al., 2012; Costa and Sebastián-Gallés, 2014; García-Pentón et al., 2014; Krizman et al., 2017).

Monolinguals’ and bilinguals’ equivalent performance on the selective attention task may seem inconsistent with previous literature showing that bilinguals perform more poorly than monolinguals on tests of speech-in-noise recognition (Mayo et al., 1997; Shi, 2010, 2012). However in the present study, the consistent structure of the sentences across trials, together with the limited number of words that could potentially appear in the sentence, likely limited the linguistic complexity, and thus, the linguistic processing demands of the task. Previous findings of a bilingual disadvantage, especially for early-acquiring, highly proficient bilinguals similar to the ones tested in the current study, used sentences that are semantically and syntactically correct, but unrestricted in their content or word choice (Mayo et al., 1997; Shi, 2010, 2012). This open-endedness increases linguistic processing demands. When the target is restricted, such as when a target word is embedded in a carrier phrase, early, high-proficiency bilinguals have been reported to perform equivalently to their monolingual peers (Krizman et al., 2017). Additionally, differences in speech-in-noise recognition between monolinguals and bilinguals are starkest when the sentences contain semantic context that can be used to ‘fill in the gaps’ (Bradlow and Bent, 2002; Bradlow and Alexander, 2007). While monolinguals are able to benefit from the semantic context contained within a degraded sentence, bilinguals benefit less from this context (Mayo et al., 1997; Bradlow and Alexander, 2007). Given that the sentences used here did not contain any semantic context to aid in disambiguation between the target and irrelevant talker, top–down semantic knowledge could not aid monolinguals’ performance on this task. Thus, the linguistic cues that lead to performance differences between bilinguals and monolinguals on speech-in-noise tasks were unavailable here.

Although the groups performed similarly, the neural analyses suggest that bilinguals and monolinguals utilize different listening strategies. Both groups showed an increase in cortical phase consistency during active listening, coupled with a decrease in subcortical phase consistency. However, relative to one another, monolinguals had greater cortical consistency, especially during active listening, while bilinguals had greater subcortical consistency, especially during passive listening.

When looking at the differences in cortical consistency between the two groups, the effect was concentrated over the theta and alpha bands. Given that the energy of the sentence envelopes used here was concentrated at 4 Hz, which is within the 3–7 Hz theta band, the theta differences are likely to reflect more consistent cortical tracking of the stimulus envelope during active listening and in monolinguals. In addition to more consistent tracking of the stimulus envelope, it is possible that the theta, and potentially alpha, differences are driven by greater cortical evoked potentials to each word in the sentence, similar to what has been demonstrated previously in bilinguals and monolinguals (Astheimer et al., 2016). Because the stimuli were designed to facilitate the subcortical FFR recording, the envelope and the cortical potentials overlap in time and frequency, and so it is difficult to disentangle the contribution of each. Another potential source of the enhanced alpha activity is the greater load that is placed on cognitive processing, particularly working memory, during active listening, consistent with previous findings that increases in alpha synchrony during a task are related to the working memory requirements of that task (Jensen et al., 2002; Jensen and Hanslmyar, 2020). If the cortical differences are tied to executive functions, the reduced cortical consistency in bilinguals may result from decreased recruitment of cortical brain regions involved in this task, similar to the reduction in activation of lateral frontal cortex and anterior cingulate cortex when doing a complex task that requires conflict monitoring, in bilinguals relative to monolinguals (Abutalebi et al., 2011; Gold et al., 2013; Costa and Sebastián-Gallés, 2014). That is, bilinguals may require less cortical processing to perform these tasks at a level that is similar to, or better than, monolinguals.

The enhanced subcortical pitch encoding for bilinguals is consistent with their previously reported F0 enhancement (Krizman et al., 2012; Skoe et al., 2017) and suggests that bilinguals are acutely tuned-in to the acoustic features, namely the F0, of a talker and use that cue to understand speech in challenging listening situations. In a competing-talkers environment, the F0 can be used to track the target talker, and was likely a useful cue here given the difference in F0 between the two talkers. The finding that active listening decreases subcortical consistency is supported by earlier work in animals finding that active listening leads to diminished responses in the inferior colliculus, the predominant contributor of the FFR (Chandrasekaran and Kraus, 2010; Bidelman, 2015, 2018; Coffey et al., 2019; White-Schwoch et al., 2019). However, a very recent study in humans found that active listening leads to increases in the FFR (Price and Bidelman, 2021). The different outcomes of these studies raise an intriguing possibility: that corticofugal tuning of the subcortical response can lead to differences in how attention effects manifest in the scalp-recorded FFR. In addition to the numerous ascending projections from the ear to the brain, there exists an even larger population of descending projections connecting the various auditory centers between the brain and the ear (Malmierca and Ryugo, 2011; Malmierca, 2015). These pathways regulate the incoming signal to meet the demands of the task, which can result in inhibition or amplification of the incoming signal depending on its task relevance (Malmierca et al., 2009, 2019; Parras et al., 2017; Ito and Malmierca, 2018). Future studies should investigate the task dependency on subcortical attention effects.

Bilinguals appear to also call upon their inhibitory control abilities during active listening, as performance on the inhibitory control and selective attention tasks was related only in this language group. We hypothesize that auditory processing and inhibitory control work in tandem to compensate, at least partly, for the greater demands that bilingualism places on language processing. This hypothesis is supported by previous findings of relationships between inhibitory control and auditory processing that are specific to bilinguals (Blumenfeld and Marian, 2011; Krizman et al., 2012, 2014; Marian et al., 2018). In contrast to bilinguals, monolinguals have greater experience with the target language and can rely more heavily on linguistic cues (e.g., linguistic context) to understand speech, particularly in difficult listening conditions (Cooke et al., 2008; Lecumberri et al., 2011; Mattys et al., 2012; Krizman et al., 2017; Strori et al., 2020). Because bilinguals are less able to benefit from these cues, we propose that they rely on non-linguistic processes, specifically sensory encoding and executive control, to overcome this disadvantage.

Although they used inhibitory control differently, bilinguals and monolinguals performed equivalently on the inhibitory control task. The similar performance between language groups contrasts with previous studies showing an inhibitory control advantage for bilinguals (Bialystok and Martin, 2004; Bialystok et al., 2005; Carlson and Meltzoff, 2008; Krizman et al., 2012, 2014); although, the evidence for a bilingual inhibitory control advantage has been equivocal, with other studies reporting that no such advantage exists (Bialystok et al., 2015; de Bruin et al., 2015; Paap et al., 2015; Dick et al., 2019). Interestingly, many (but not all) studies that do find an advantage tend to find it when comparing bilingual and monolingual participants at the ends of the lifespan (i.e., young children and older adults) while many that do not find an advantage have looked for performance differences in young adults. This may suggest that bilinguals and monolinguals eventually reach the same level of inhibitory control abilities but that bilinguals mature to this level at a faster rate (and decline from this level more slowly later in life). Given that inhibitory control is malleable with other enriching life experiences, such as music training (Moreno et al., 2014; Slater et al., 2018) or sports participation (Lind et al., 2019; Hagyard et al., 2021), it may be more difficult to isolate the bilingual enhancement when looking across individuals from different backgrounds, especially with increasing age and enrichment. If bilinguals and monolinguals use this skill differently in everyday settings, it could explain the re-emergence of a bilingual inhibitory-control advantage in older adults (Bialystok et al., 2005).

### Separating High Gamma and Pitch-Contour Tracking

There is general consensus that the response to the female voice is subcortical in origin, given the higher frequency of that voice (∼220 Hz) and that cortical phase-locking limitations preclude reliable firing to this frequency (Aiken and Picton, 2008; Akhoun et al., 2008; Bidelman, 2018). However, there is still some debate about the origins of the response to the male voice in this study, given the overlap between the high gamma frequency range and the pitch of the male talker. Nevertheless, these responses are presumed to originate from distinct sources (Edwards et al., 2005; Chandrasekaran and Kraus, 2010; Mesgarani and Chang, 2012; White-Schwoch et al., 2019). To determine whether these responses indeed reflect distinct sources, we compared them to see if they differed from one another and if they were affected differently by different stimulus and protocol parameters. We did observe differences between the high gamma and pitch contour responses. We found that high gamma consistency did not differ between the two listening conditions and that across the two conditions and the four words (‘ready,’ ‘go,’ ‘to,’ ‘now’), it was lower than the consistency to the male pitch contour. Similar to the response to the female pitch contour, consistency of the response to the male pitch contour increased during passive listening. Given that the female pitch contour is above the cortical phase-locking limits but within subcortical phase-locking limits, it is presumed to arise from subcortical sources (Liang-Fa et al., 2006; Chandrasekaran and Kraus, 2010; Coffey et al., 2016, 2019). The difference between the high gamma and male pitch contour consistency effects, together with the similar effects of listening condition on the male and female pitch contours suggest that these pitch contour responses arise from similar subcortical sources (White-Schwoch et al., 2019). Furthermore, a 10 ms-lag was used to analyze pitch-tracking consistency to align the analyses with the temporal lag between the sound reaching the ear and reaching the brainstem, a much faster lag than that seen for the cortex, which arises ∼40 ms after the stimulus is first heard (Langner and Schreiner, 1988; Liegeois-Chauvel et al., 1994; Coffey et al., 2016). Although the clearest way to classify the activity as distinctly cortical and subcortical would be through either source localization or direct simultaneous recordings from the regions of interest in an animal model, these methods used to analyze these responses and the differences found between high-gamma and pitch-tracking activity support the hypothesis that these responses arise from distinct cortical and subcortical sources, respectively. We acknowledge, however, that there still is some debate about the origins of the FFR, when evoked at frequencies around those of the male speaker, as some MEG studies suggest that there is a cortical contribution to the FFR at that frequency range in addition to a larger subcortical contribution, which may influence findings of attention effects on FFR (Bidelman, 2018; Coffey et al., 2019; Hartmann and Weisz, 2019).

## Conclusion

In conclusion, results from this study are consistent with the hypothesis that bilinguals utilize their cognitive and sensory enhancements for active listening. We found that monolingual and bilingual adolescents and young adults differed in the neural and cognitive processes engaged to perform a selective attention task, yet performed similarly on the task. Specifically, although both groups showed an increase in cortical phase consistency during active listening, coupled with a decrease in subcortical phase consistency, relative to one another, monolinguals had greater cortical consistency, especially during active listening, while bilinguals had greater subcortical consistency. Also, bilinguals showed a relationship between performance on the inhibitory control and selective attention tests, while monolinguals did not. The neural findings highlight an interesting distinction between online and lifelong modulation of midbrain auditory processing. The bilingual enhancement coupled with the active-listening suppression suggest that different mechanisms underlie short-term and long-term changes in subcortical auditory processing.

## Data Availability Statement

The raw data supporting the conclusions of this article will be made available by the authors, without undue reservation.

## Ethics Statement

The studies involving human participants were reviewed and approved by Northwestern University Institutional Review Board. Written informed consent to participate in this study was provided by participants 18 and older while informed written assent was given by adolescents younger than 18 and consent provided by the participants’ legal guardian/next of kin.

## Author Contributions

JK, AT, TN, and NK: conceptualization, methodology, writing – review and editing. JK and AT: investigation. JK: project administration and writing – original draft. JK, AT, and TN: visualization. JK and NK: funding acquisition. NK: supervision. All authors contributed to the article and approved the submitted version.

## Conflict of Interest

The authors declare that the research was conducted in the absence of any commercial or financial relationships that could be construed as a potential conflict of interest.

## Publisher’s Note

All claims expressed in this article are solely those of the authors and do not necessarily represent those of their affiliated organizations, or those of the publisher, the editors and the reviewers. Any product that may be evaluated in this article, or claim that may be made by its manufacturer, is not guaranteed or endorsed by the publisher.

## Footnotes

1
www.braintrain.com

2
http://audacity.sourceforge.net

3
http://www.fon.hum.uva.nl/praat/

## References

[B1] Abutalebi J. Della Rosa P. A. Green D. W. Hernandez M. Scifo P. Keim R. (2011). Bilingualism tunes the anterior cingulate cortex for conflict monitoring. *Cereb. Cortex* 22 2076–2086. 10.1093/cercor/bhr287 22038906

[B2] Abutalebi J. Green D. W. (2007). Bilingual language production: the neurocognition of language representation and control. *J. Neurolinguistics* 20 242–275. 10.1016/j.jneuroling.2006.10.003

[B3] Aiken S. J. Picton T. W. (2008). Envelope and spectral frequency-following responses to vowel sounds. *Hear. Res.* 245 35–47. 10.1016/j.heares.2008.08.004 18765275

[B4] Akhoun I. Gallego S. Moulin A. Menard M. Veuillet E. Berger-Vachon C. (2008). The temporal relationship between speech auditory brainstem responses and the acoustic pattern of the phoneme /ba/ in normal-hearing adults. *Clin. Neurophysiol.* 119 922–933. 18291717 10.1016/j.clinph.2007.12.010

[B5] Alain C. Woods D. L. (1999). Age-related changes in processing auditory stimuli during visual attention: evidence for deficits in inhibitory control and sensory memory. *Psychol. Aging* 14 507–19. 10509703 10.1037//0882-7974.14.3.507

[B6] Anderson S. Skoe E. Chandrasekaran B. Zecker S. Kraus N. (2010). Brainstem correlates of speech-in-noise perception in children. *Hear. Res.* 270 151–157. 10.1016/j.heares.2010.08.001 20708671 PMC2997182

[B7] Astheimer L. B. Berkes M. Bialystok E. (2016). Differential allocation of attention during speech perception in monolingual and bilingual listeners. *Lang. Cogn. Neurosci.* 31 196–205. 27110579 10.1080/23273798.2015.1083114 PMC4838403

[B8] Baddeley A. (2003). Working memory and language: an overview. *J. Commun. Disord.* 36 189–208. 12742667 10.1016/s0021-9924(03)00019-4

[B9] Bialystok E. (2009). Bilingualism: the good, the bad, and the indifferent. *Biling. Lang. Cogn.* 12 3–11.

[B10] Bialystok E. (2011). Reshaping the mind: the benefits of bilingualism. *Can. J. Exp. Psychol.* 65 229–235. 10.1037/a0025406 21910523 PMC4341987

[B11] Bialystok E. (2015). Bilingualism and the development of executive function: the role of attention. *Child Dev. Pers.* 9 117–121. 10.1111/cdep.12116 26019718 PMC4442091

[B12] Bialystok E. Kroll J. F. Green D. W. MacWhinney B. Craik F. I. (2015). Publication bias and the validity of evidence what’s the connection? *Psychol. Sci.* 26 944–6. 10.1177/0956797615573759 25944774

[B13] Bialystok E. Martin M. M. (2004). Attention and inhibition in bilingual children: evidence from the dimensional change card sort task. *Dev. Sci.* 7 325–339. 10.1111/j.1467-7687.2004.00351.x 15595373

[B14] Bialystok E. Martin M. M. Viswanathan M. (2005). Bilingualism across the lifespan: the rise and fall of inhibitory control. *Int. J. Biling.* 9 103–119.

[B15] Bialystok E. Viswanathan M. (2009). Components of executive control with advantages for bilingual children in two cultures. *Cognition* 112 494–500. 19615674 10.1016/j.cognition.2009.06.014 PMC2755257

[B16] Bidelman G. M. (2015). Multichannel recordings of the human brainstem frequency-following response: scalp topography, source generators, and distinctions from the transient ABR. *Hear. Res.* 323 68–80. 10.1016/j.heares.2015.01.011 25660195

[B17] Bidelman G. M. (2018). Subcortical sources dominate the neuroelectric auditory frequency-following response to speech. *Neuroimage* 175 56–69. 10.1016/j.neuroimage.2018.03.060 29604459

[B18] Bird J. Darwin C. (1998). “Effects of a difference in fundamental frequency in separating two sentences,” in *Psychophysical and Physiological Advances in Hearing*, eds Palmer A. R. Rees A. Summerfield A. Q. Meddis R., (London: Whurr), 263–269.

[B19] Blumenfeld H. K. Marian V. (2011). Bilingualism influences inhibitory control in auditory comprehension. *Cognition* 118 245–257. 10.1016/j.cognition.2010.10.012 21159332 PMC3582323

[B20] Bolia R. S. Nelson W. T. Ericson M. A. Simpson B. D. (2000). A speech corpus for multitalker communications research. *J. Acoust. Soc. Am.* 107 1065–6. 10.1121/1.428288 10687719

[B21] Bradlow A. R. Alexander J. A. (2007). Semantic and phonetic enhancements for speech-in-noise recognition by native and non-native listeners. *J. Acoust. Soc. Am.* 121 2339–49. 10.1121/1.2642103 17471746

[B22] Bradlow A. R. Bent T. (2002). The clear speech effect for non-native listeners. *J. Acoust. Soc. Am.* 112 272–84. 10.1121/1.1487837 12141353

[B23] Bregman A. S. Liao C. Levitan R. (1990). Auditory grouping based on fundamental frequency and formant peak frequency. *Can. J. Psychol.* 44 400–13. 10.1037/h0084255 2224643

[B24] Carlson S. M. Meltzoff A. N. (2008). Bilingual experience and executive functioning in young children. *Dev. Sci.* 11 282–298. 18333982 10.1111/j.1467-7687.2008.00675.x PMC3647884

[B25] Casseday J. H. Fremouw T. Covey E. (2002). “The inferior colliculus: a hub for the central auditory system,” in *Integrative Functions in the Mammalian Auditory Pathway*, eds Oertel D. Fay R. R. Popper A. N., (New York: Springer), 238–318. 10.1007/978-1-4757-3654-0_7

[B26] Cervenka M. C. Nagle S. Boatman-Reich D. (2011). Cortical high-gamma responses in auditory processing. *Am. J. Audiol.* 20 171–180. 10.1044/1059-0889(2011/10-0036) 22158634 PMC3848128

[B27] Chandrasekaran B. Kraus N. (2010). The scalp-recorded brainstem response to speech: neural origins and plasticity. *Psychophysiology* 47 236–246. 10.1111/j.1469-8986.2009.00928.x 19824950 PMC3088516

[B28] Coffey E. B. Herholz S. C. Chepesiuk A. M. Baillet S. Zatorre R. J. (2016). Cortical contributions to the auditory frequency-following response revealed by MEG. *Nat. Commun.* 7:11070. 10.1038/ncomms11070 PMC4820836 27009409

[B29] Coffey E. B. Nicol T. White-Schwoch T. Chandrasekaran B. Krizman J. Skoe E. (2019). Evolving perspectives on the sources of the frequency-following response. *Nat. Commun.* 10:5036. 10.1038/s41467-019-13003-w 31695046 PMC6834633

[B30] Collet L. Duclaux R. (1986). Auditory brainstem evoked responses and attention: contribution to a controversial subject. *Acta Otolaryngol. (Stockh.)* 101 439–441. 3727978 10.3109/00016488609108629

[B31] Conway C. M. Pisoni D. B. Kronenberger W. G. (2009). The importance of sound for cognitive sequencing abilities. *Curr. Direct. Psychol. Sci.* 18 275–279. 10.1111/j.1467-8721.2009.01651.x 20725604 PMC2923391

[B32] Cooke M. Lecumberri M. L. G. Barker J. (2008). The foreign language cocktail party problem: energetic and informational masking effects in non-native speech perception. *J. Acoust. Soc. Am.* 123 414–27. 10.1121/1.2804952 18177170

[B33] Costa A. Sebastián-Gallés N. (2014). How does the bilingual experience sculpt the brain? *Nat. Rev. Neurosci.* 15 336–345. 10.1038/nrn3709 24739788 PMC4295724

[B34] Crittenden B. M. Duncan J. (2014). Task difficulty manipulation reveals multiple demand activity but no frontal lobe hierarchy. *Cereb. Cortex* 24 532–540. 10.1093/cercor/bhs333 23131804 PMC3888372

[B35] D’Angiulli A. Herdman A. Stapells D. Hertzman C. (2008). Children’s event-related potentials of auditory selective attention vary with their socioeconomic status. *Neuropsychology* 22 293–300. 10.1037/0894-4105.22.3.293 18444707

[B36] Darwin C. J. (1997). Auditory grouping. *Trends Cogn. Sci.* 1 327–333. 21223942 10.1016/S1364-6613(97)01097-8

[B37] Darwin C. J. Brungart D. S. Simpson B. D. (2003). Effects of fundamental frequency and vocal-tract length changes on attention to one of two simultaneous talkers. *J. Acoust. Soc. Am.* 114 2913–2922. 10.1121/1.1616924 14650025

[B38] de Abreu P. M. E. Cruz-Santos A. Tourinho C. J. Martin R. Bialystok E. (2012). Bilingualism enriches the poor enhanced cognitive control in low-income minority children. *Psychol. Sci.* 23 1364–1371. 10.1177/0956797612443836 23044796 PMC4070309

[B39] de Bruin A. Treccani B. Della Sala S. (2015). Cognitive advantage in bilingualism an example of publication bias? *Psychol. Sci.* 26 99–107. 10.1177/0956797614557866 25475825

[B40] Dick A. S. Garcia N. L. Pruden S. M. Thompson W. K. Hawes S. W. Sutherland M. T. (2019). No evidence for a bilingual executive function advantage in the ABCD study. *Nat. Hum. Behav.* 3 692–701. 10.1038/s41562-019-0609-3 31110341 PMC7156280

[B41] Ding N. Simon J. Z. (2014). Cortical entrainment to continuous speech: functional roles and interpretations. *Front. Hum. Neurosci.* 8:311. 10.3389/fnhum.2014.00311 24904354 PMC4036061

[B42] Edwards E. Soltani M. Deouell L. Y. Berger M. S. Knight R. T. (2005). High gamma activity in response to deviant auditory stimuli recorded directly from human cortex. *J. Neurophysiol.* 94 4269–4280. 16093343 10.1152/jn.00324.2005

[B43] Figueras B. Edwards L. Langdon D. (2008). Executive function and language in deaf children. *J. Deaf Stud. Deaf Educ.* 13 362–377. 10.1093/deafed/enm067 18252699

[B44] Forte A. E. Etard O. Reichenbach T. (2017). The human auditory brainstem response to running speech reveals a subcortical mechanism for selective attention. *Elife* 6:e27203. 10.7554/eLife.27203 28992445 PMC5634786

[B45] Foy J. G. Mann V. A. (2014). Bilingual children show advantages in nonverbal auditory executive function task. *Int. J. Biling.* 18 717–729.

[B46] Fritz J. B. Elhilali M. David S. V. Shamma S. A. (2007). Auditory attention - focusing the searchlight on sound. *Curr. Opin. Neurobiol.* 17 437–455. 10.1016/j.conb.2007.07.011 17714933

[B47] Galbraith G. C. Bhuta S. M. Choate A. K. Kitahara J. M. Mullen T. A. Jr. (1998). Brain stem frequency-following response to dichotic vowels during attention. *Neuroreport* 9 1889–1893. 10.1097/00001756-199806010-00041 9665621

[B48] García-Pentón L. Fernández A. P. Iturria-Medina Y. Gillon-Dowens M. Carreiras M. (2014). Anatomical connectivity changes in the bilingual brain. *Neuroimage* 84 495–504. 24018306 10.1016/j.neuroimage.2013.08.064

[B49] Gathercole S. E. Baddeley A. D. (2014). *Working Memory and Language.* East Sussex: Psychology Press.

[B50] Gold B. T. Kim C. Johnson N. F. Kryscio R. J. Smith C. D. (2013). Lifelong bilingualism maintains neural efficiency for cognitive control in aging. *J. Neurosci.* 33 387–396. 10.1523/JNEUROSCI.3837-12.2013 23303919 PMC3710134

[B51] Golumbic E. M. Z. Ding N. Bickel S. Lakatos P. Schevon C. A. McKhann G. M. (2013). Mechanisms underlying selective neuronal tracking of attended speech at a “cocktail party”. *Neuron* 77 980–991. 10.1016/j.neuron.2012.12.037 23473326 PMC3891478

[B52] Hagyard J. Brimmell J. Edwards E. J. Vaughan R. S. (2021). Inhibitory control across athletic expertise and its relationship with sport performance. *J. Sport Exerc. Psychol.* 43 14–27. 10.1123/jsep.2020-0043 33383568

[B53] Hart B. Risley T. R. (1995). *Meaningful Differences in the Everyday Experience of Young American Children.* Baltimore: Paul H Brookes Publishing.

[B54] Hartmann T. Weisz N. (2019). Auditory cortical generators of the Frequency Following Response are modulated by intermodal attention. *Neuroimage* 203:116185. 10.1016/j.neuroimage.2019.116185 31520743

[B55] Hoff E. (2003). The specificity of environmental influence: socioeconomic status affects early vocabulary development via maternal speech. *Child Dev.* 74 1368–1378. 10.1111/1467-8624.00612 14552403

[B56] Hollingshead A. (1975). *Four Factor Index of Social Status.* New Haven: Yale University.

[B57] Hopfinger J. Buonocore M. Mangun G. (2000). The neural mechanisms of top-down attentional control. *Nat. Neurosci.* 3 284–291. 10700262 10.1038/72999

[B58] Ito T. Malmierca M. S. (2018). “Neurons, connections, and microcircuits of the inferior colliculus,” in *The Mammalian Auditory Pathways*, eds Oliver D. Cant N. Fay R. Popper A. (Cham: Springer), 127–167.

[B59] Jensen O. Gelfand J. Kounios J. Lisman J. E. (2002). Oscillations in the alpha band (9–12 Hz) increase with memory load during retention in a short-term memory task. *Cereb. Cortex* 12 877–882. 10.1093/cercor/12.8.877 12122036

[B60] Jensen O. Hanslmyar S. (2020). “The role of alpha oscillations for attention and working memory,” in *The Cognitive Neurosciences*, eds Poeppel D. Mangun G. R. Gazzaniga M. S. (Cambridge, MA: MIT Press), 323.

[B61] Jürgens U. (1983). Afferent fibers to the cingular vocalization region in the squirrel monkey. *Exp. Neurol.* 80 395–409. 10.1016/0014-4886(83)90291-1 6840246

[B62] Kaushanskaya M. Blumenfeld H. K. Marian V. (2019). The Language Experience and Proficiency Questionnaire (LEAP-Q): Ten years later. *Biling. (Camb Engl)* 23 945–950. 33628083 10.1017/s1366728919000038 PMC7899192

[B63] Khan S. Edwards L. Langdon D. (2005). The cognition and behaviour of children with cochlear implants, children with hearing aids and their hearing peers: a comparison. *Audiol. Neurootol.* 10 117–126. 15650303 10.1159/000083367

[B64] Kral A. Kronenberger W. G. Pisoni D. B. O’Donoghue G. M. (2016). Neurocognitive factors in sensory restoration of early deafness: a connectome model. *Lancet Neurol.* 15 610–621. 10.1016/s1474-4422(16)00034-x 26976647 PMC6260790

[B65] Krishnan A. Gandour J. T. Bidelman G. M. Swaminathan J. (2009). Experience-dependent neural representation of dynamic pitch in the brainstem. *Neuroreport* 20 408–413. 10.1097/wnr.0b013e3283263000 19223793 PMC2692950

[B66] Krishnan A. Xu Y. Gandour J. Cariani P. (2005). Encoding of pitch in the human brainstem is sensitive to language experience. *Cogn. Brain Res.* 25 161–168. 10.1016/j.cogbrainres.2005.05.004 15935624

[B67] Krizman J. Bradlow A. R. Lam S. S.-Y. Kraus N. (2017). How bilinguals listen in noise: linguistic and non-linguistic factors. *Biling. Lang. Cogn.* 20 834–843. 10.1017/s1366728916000444

[B68] Krizman J. Marian V. Shook A. Skoe E. Kraus N. (2012). Subcortical encoding of sound is enhanced in bilinguals and relates to executive function advantages. *Proc. Natl. Acad. Sci.* 109 7877–7881. 10.1073/pnas.1201575109 22547804 PMC3356657

[B69] Krizman J. Skoe E. Marian V. Kraus N. (2014). Bilingualism increases neural response consistency and attentional control: evidence for sensory and cognitive coupling. *Brain Lang.* 128 34–40. 10.1016/j.bandl.2013.11.006 24413593 PMC3923605

[B70] Kroll J. F. Bobb S. C. Misra M. Guo T. (2008). Language selection in bilingual speech: evidence for inhibitory processes. *Acta Psychol. (Amst.)* 128 416–430. 10.1016/j.actpsy.2008.02.001 18358449 PMC2585366

[B71] Kronenberger W. G. Xu H. Pisoni D. B. (2020). Longitudinal development of executive functioning and spoken language skills in preschool-aged children with cochlear implants. *J. Speech Lang. Hear. Res.* 63 1128–1147. 10.1044/2019_JSLHR-19-00247 32204645 PMC7242982

[B72] Langner G. Schreiner C. E. (1988). Periodicity coding in the inferior colliculus of the cat. I. Neuronal mechanisms. *J. Neurophysiol.* 60 1799–1822. 10.1152/jn.1988.60.6.1799 3236052

[B73] Lecumberri M. L. G. Cooke M. Cutler A. (2011). Non-native speech perception in adverse conditions: a review. *Speech Commun.* 52 864–886. 10.1016/j.specom.2010.08.014

[B74] Liang-Fa L. Alan R. P. Mark N. W. (2006). Phase-locked responses to pure tones in the inferior colliculus. *J. Neurophysiol.* 95 1926–35. 10.1152/jn.00497.2005 16339005

[B75] Liegeois-Chauvel C. Musolino A. Badier J. Marquis P. Chauvel P. (1994). Evoked potentials recorded from the auditory cortex in man: evaluation and topography of the middle latency components. *Electroencephalogr. Clin. Neurophysiol.* 92 204–214. 10.1016/0168-5597(94)90064-7 7514990

[B76] Lind R. R. Beck M. M. Wikman J. Malarski K. Krustrup P. Lundbye-Jensen J. (2019). Acute high-intensity football games can improve children’s inhibitory control and neurophysiological measures of attention. *Scand. J. Med. Sci. Sports* 29 1546–1562. 10.1111/sms.13485 31125468 PMC6852517

[B77] Lucks Mendel L. Widner H. (2016). Speech perception in noise for bilingual listeners with normal hearing. *Int. J. Audiol.* 55 126–134. 10.3109/14992027.2015.1061710 26189557

[B78] Malmierca M. S. (2015). “Anatomy and physiology of the mammalian auditory system,” in *Encyclopedia of Computational Neuroscience*, eds Jaeger D. Jung R., (New York: Springer), 155–186. 10.1007/978-1-4614-6675-8_286

[B79] Malmierca M. S. Cristaudo S. érez-González D. P. Covey E. (2009). Stimulus-specific adaptation in the inferior colliculus of the anesthetized rat. *J. Neurosci.* 29 5483–5493. 10.1523/jneurosci.4153-08.2009 19403816 PMC2715893

[B80] Malmierca M. S. Nino-Aguillon B. E. Nieto-Diego J. Porteros Á Pérez-González D. Escera C. (2019). Pattern-sensitive neurons reveal encoding of complex auditory regularities in the rat inferior colliculus. *Neuroimage* 184 889–900. 10.1016/j.neuroimage.2018.10.012 30296562

[B81] Malmierca M. S. Ryugo D. K. (2011). “Descending connections of auditory cortex to the midbrain and brain stem,” in *The Auditory Cortex*, eds Winer J. Schreiner C., (New York: Springer), 189–208. 10.1007/978-1-4419-0074-6_9

[B82] Marian V. Blumenfeld H. K. Kaushanskaya M. (2007). The language experience and proficiency questionnaire (LEAP-Q): assessing language profiles in bilinguals and multilinguals. *J. Speech Lang. Hear. Res.* 50 940–67. 10.1044/1092-4388(2007/067) 17675598

[B83] Marian V. Lam T. Q. Hayakawa S. Dhar S. (2018). Top-down cognitive and linguistic influences on the suppression of spontaneous otoacoustic emissions. *Front. Neurosci.* 12:378. 10.3389/fnins.2018.00378 29937708 PMC6002685

[B84] Marian V. Spivey M. (2003). Bilingual and monolingual processing of competing lexical items. *Appl. Psycholing.* 24 173–193. 10.1017/s0142716403000092

[B85] Mattys S. L. Davis M. H. Bradlow A. R. Scott S. K. (2012). Speech recognition in adverse conditions: a review. *Lang. Cognit. Process.* 27 953–978. 10.1080/01690965.2012.705006

[B86] Mayo L. H. Florentine M. Buus S. (1997). Age of second-language acquisition and perception of speech in noise. *J. Speech Lang. Hear. Res.* 40 686–93. 10.1044/jslhr.4003.686 9210123

[B87] Mechelli A. Crinion J. T. Noppeney U. O’Doherty J. Ashburner J. Frackowiak R. S. (2004). Neurolinguistics: structural plasticity in the bilingual brain. *Nature* 431 757–757. 15483594 10.1038/431757a

[B88] Mesgarani N. Chang E. F. (2012). Selective cortical representation of attended speaker in multi-talker speech perception. *Nature* 485 233–236. 22522927 10.1038/nature11020 PMC3870007

[B89] Mitchell T. V. Quittner A. L. (1996). Multimethod study of attention and behavior problems in hearing-impaired children. *J. Clin. Child Psychol.* 25 83–96.

[B90] Moreno S. Wodniecka Z. Tays W. Alain C. Bialystok E. (2014). Inhibitory control in bilinguals and musicians: event related potential (ERP) evidence for experience-specific effects. *PLoS One* 9:e94169. 10.1371/journal.pone.0094169 24743321 PMC3990547

[B91] Morini G. (2020). Examining bilingual speech perception and the role of background noise. *J. Acoust. Soc. Am.* 148:2623.

[B92] Neill W. Valdes L. Terry K. (1995). “Selective attention and the inhibitory control of cognition,” in *Interference and Inhibition in Cognition.* eds Dempster F. N. Brainerd C. J., (San Diego: Academic Press), 207–261.

[B93] Olguin A. Cekic M. Bekinschtein T. A. Katsos N. Bozic M. (2019). Bilingualism and language similarity modify the neural mechanisms of selective attention. *Sci. Rep.* 9:8204. 10.1038/s41598-019-44782-3 PMC6547874 31160645

[B94] Osterhammel P. Shallop J. Terkildsen K. (1985). The effect of sleep on the auditory brainstem response (ABR) and the middle latency response (MLR). *Scand. Audiol.* 14 47–50. 10.3109/01050398509045921 4059844

[B95] Paap K. R. Johnson H. A. Sawi O. (2015). Bilingual advantages in executive functioning either do not exist or are restricted to very specific and undetermined circumstances. *Cortex* 69 265–278. 10.1016/j.cortex.2015.04.014 26048659

[B96] Parras G. G. Nieto-Diego J. Carbajal G. V. Valdés-Baizabal C. Escera C. Malmierca M. S. (2017). Neurons along the auditory pathway exhibit a hierarchical organization of prediction error. *Nat. Commun.* 8:2148. 10.1038/s41467-017-02038-6 29247159 PMC5732270

[B97] Pichora-Fuller M. K. Kramer S. E. Eckert M. A. Edwards B. Hornsby B. W. Humes L. E. (2016). Hearing impairment and cognitive energy: the framework for understanding effortful listening (FUEL). *Ear Hear.* 37 5S–27S. 27355771 10.1097/AUD.0000000000000312

[B98] Price C. N. Bidelman G. M. (2021). Attention reinforces human corticofugal system to aid speech perception in noise. *Neuroimage* 235:118014. 10.1016/j.neuroimage.2021.118014 33794356 PMC8274701

[B99] Raizada R. D. Poldrack R. A. (2007). Challenge-driven attention: interacting frontal and brainstem systems. *Front. Hum. Neurosci.* 1:3. 10.3389/neuro.09.003.2007 18958217 PMC2525983

[B100] Ressel V. Pallier C. Ventura-Campos N. Díaz B. Roessler A. Ávila C. (2012). An effect of bilingualism on the auditory cortex. *J. Neurosci.* 32 16597–16601. 10.1523/jneurosci.1996-12.2012 23175815 PMC6621793

[B101] Salamy A. McKean C. M. (1977). Habituation and dishabituation of cortical and brainstem evoked potentials. *Int. J. Neurosci.* 7 175–182. 10.3109/00207457709147665

[B102] Shi L.-F. (2010). Perception of acoustically degraded sentences in bilingual listeners who differ in age of English acquisition. *J. Speech Lang. Hear. Res.* 53 821–35. 10.1044/1092-4388(2010/09-0081) 20220026

[B103] Shi L.-F. (2012). Contribution of linguistic variables to bilingual listeners’ perception of degraded english sentences. *J. Speech Lang. Hear. Res.* 55 219–34. 10.1044/1092-4388(2011/10-0240) 22199200

[B104] Skoe E. (2019). Turn up the volume: speech perception in noise for bilingual listeners. *J. Acoust. Soc. Am.* 145 1820–1820.

[B105] Skoe E. Burakiewicz E. Figueiredo M. Hardin M. (2017). Basic neural processing of sound in adults is influenced by bilingual experience. *Neuroscience* 349 278–290. 10.1016/j.neuroscience.2017.02.049 28259798

[B106] Skoe E. Karayanidi K. (2019). Bilingualism and speech understanding in noise: auditory and linguistic factors. *J. Am. Acad. Audiol.* 30 115–130. 30461397 10.3766/jaaa.17082

[B107] Slater J. Ashley R. Tierney A. Kraus N. (2018). Got rhythm? Better inhibitory control is linked with more consistent drumming and enhanced neural tracking of the musical beat in adult percussionists and nonpercussionists. *J. Cogn. Neurosci.* 30 14–24. 10.1162/jocn_a_01189 28949825

[B108] Slee S. J. David S. V. (2015). Rapid task-related plasticity of spectrotemporal receptive fields in the auditory midbrain. *J. Neurosci.* 35 13090–13102. 10.1523/JNEUROSCI.1671-15.2015 26400939 PMC4579375

[B109] Song J. H. Skoe E. Banai K. Kraus N. (2010). Perception of speech in noise: neural correlates. *J. Cogn. Neurosci.* 23 2268–2279. 20681749 10.1162/jocn.2010.21556 PMC3253852

[B110] Song J. H. Skoe E. Wong P. C. M. Kraus N. (2008). Plasticity in the adult human auditory brainstem following short-term linguistic training. *J. Cogn. Neurosci.* 20 1892–1902. 18370594 10.1162/jocn.2008.20131 PMC2829864

[B111] Spivey M. Marian V. (1999). Cross talk between native and second languages: partial activation of an irrelevant lexicon. *Psychol. Sci.* 10 281–284. 10.1111/1467-9280.00151

[B112] Strori D. Bradlow A. R. Souza P. E. (2020). Recognition of foreign-accented speech in noise: the interplay between talker intelligibility and linguistic structure. *J. Acoust. Soc. Am.* 147 3765–3782. 10.1121/10.0001194 32611135 PMC7275869

[B113] Stuart A. Zhang J. Swink S. (2010). Reception thresholds for sentences in quiet and noise for monolingual english and bilingual Mandarin-English listeners. *J. Am. Acad. Audiol.* 21 239–248. 10.3766/jaaa.21.4.3 20388450

[B114] Thierry G. Wu Y. J. (2007). Brain potentials reveal unconscious translation during foreign-language comprehension. *Proc. Natl. Acad. Sci. U. S. A.* 104 12530–12535. 10.1073/pnas.0609927104 17630288 PMC1941503

[B115] Tun P. A. O’Kane G. Wingfield A. (2002). Distraction by competing speech in young and older adult listeners. *Psychol. Aging* 17 453–67. 12243387 10.1037//0882-7974.17.3.453

[B116] Van Heuven W. J. Schriefers H. Dijkstra T. Hagoort P. (2008). Language conflict in the bilingual brain. *Cereb. Cortex* 18 2706–2716. 18424776 10.1093/cercor/bhn030 PMC2567421

[B117] Varghese L. Bharadwaj H. M. Shinn-Cunningham B. G. (2015). Evidence against attentional state modulating scalp-recorded auditory brainstem steady-state responses. *Brain Res.* 1626 146–164. 10.1016/j.brainres.2015.06.038 26187756 PMC5645016

[B118] Wechsler D. (1999). *Wechsler Abbreviated Scale of Intelligence (WASI).* San Antonio: Harcourt Assessment.

[B119] Weissman D. H. Roberts K. C. Visscher K. M. Woldorff M. G. (2006). The neural bases of momentary lapses in attention. *Nat. Neurosci.* 9 971–978. 10.1038/nn1727 16767087

[B120] White-Schwoch T. Anderson S. Krizman J. Nicol T. Kraus N. (2019). Case studies in neuroscience: subcortical origins of the frequency-following response. *J. Neurophysiol.* 122 844–848. 10.1152/jn.00112.2019 31268800

[B121] Wolfe C. D. Bell M. A. (2004). Working memory and inhibitory control in early childhood: contributions from physiology, temperament, and language. *Dev. Psychobiol.* 44 68–83. 10.1002/dev.10152 14704991

[B122] Wu C. Weissman D. Roberts K. Woldorff M. (2007). The neural circuitry underlying the executive control of auditory spatial attention. *Brain Res.* 1134 187–98. 10.1016/j.brainres.2006.11.088 17204249 PMC3130498

