# Supplementary material for: Listening in the Moment: How Bilingualism Interacts With Task Demands to Shape Active Listening
Source: Front Neurosci. 2021 Dec 10;15:717572. doi: 10.3389/fnins.2021.717572 (PMC8702653; doi:10.3389/fnins.2021.717572)
Supplement: Supplementary file 1 [file Data_Sheet_1.PDF]

frontiers

# *Supplementary Material*

## 1 Supplementary Figures and Tables

Below are plots and statistics for the remaining significant interactions from the cortical phase consistency 2 (language group: Monolingual, Bilingual) x 2 (condition: Active, Passive) x 3 (electrode: Fz, Cz, Pz) x 2 (pitch contour: Male, Female) x 4 (word: 'Ready', 'Go', 'To', 'Now') RMANOVA. For 2-way interactions, post hoc statistics are provided in table form. For 3-way interactions, a summary of the post-hoc statistics are provided in the text. Due to the volume of post-hocs generated by the 4-way interactions and that these tests echo what is reported in the simpler analyses, we do not report these post hocs. All post hocs are Bonferroni-corrected. Mean ± 1 standard deviation for all factors are provided in tables.

### 1.1 Cortical

#### 1.1.1 Listening Condition by Word Interaction

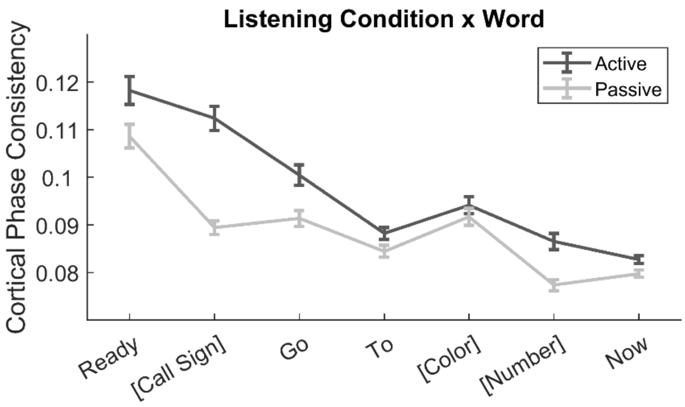


**Supplementary Figure 1.** Listening condition by word interaction plot. Cortical phase consistency is on the y axis and word is on the x axis. Active is in dark gray and passive is in light gray. Collapsing across all frequency bands, electrodes, and language groups, there was an increase in active-condition cortical phase consistency at the call sign and number.

| | Active | Passive | R | T | p | d |
|---|---|---|---|---|---|---|
| **Ready** | 0.118 ± 0.07 | 0.109 ± 0.06 | 0.443 | 2.145 | .038 | 0.137 |
| **Call Sign** | **0.112 ± 0.06** | **0.089 ± 0.04** | **0.464** | **8.375** | **<.0005** | **0.412** |
| **Go** | 0.101 ± 0.05 | 0.091 ± 0.04 | 0.318 | 2.331 | .025 | 0.165 |
| **To** | 0.088 ± 0.03 | 0.085 ± 0.03 | 0.006 | 1.184 | .243 | 0.086 |
| **Color** | 0.094 ± 0.04 | 0.917 ± 0.04 | 0.574 | 0.953 | .347 | 0.060 |
| **Number** | **0.087 ± 0.04** | **0.077 ± 0.03** | **0.325** | **3.338** | **.002** | **0.215** |
| **Now** | 0.083 ± 0.02 | 0.080 ± 0.02 | 0.505 | 2.383 | .022 | 0.149 |

**Supplementary Table 1.** Listening condition by word mean + 1 standard deviation and post hoc tests. Bonferroni-corrected significant effects are indicated in bold numbers. Degrees of freedom for all tests equals 39.

### 1.1.2 Electrode by Word Interaction

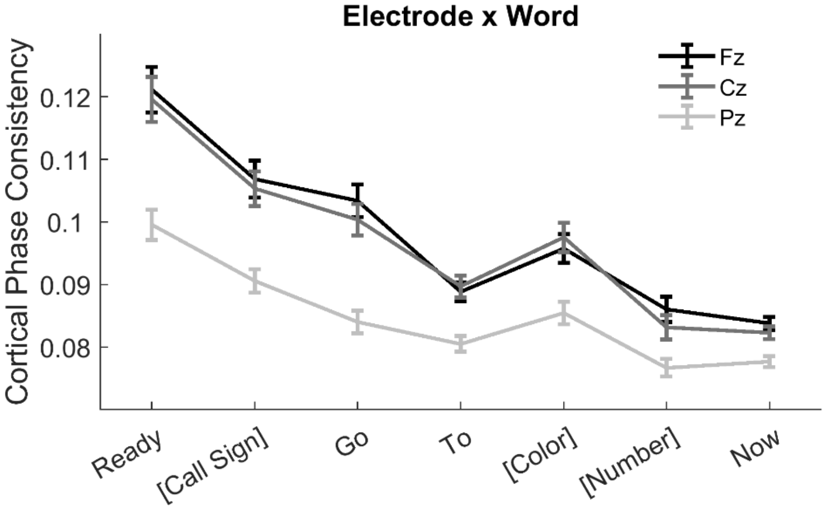


**Supplementary Figure 2.** Electrode by word interaction plot. In this figure, cortical phase consistency is on the y axis, while word is on the x axis. Fz is plotted in black, Cz is plotted in dark gray, and Pz is plotted in light gray. Collapsing across active and passive listening conditions, language group, and electrode, there was a difference in phase consistency across electrodes. This difference was driven by Pz being significantly smaller than Fz and Cz, with the magnitude of this effect being largest at the call sign and color (see Supplementary Table 2).

| | **Mean ± 1 Standard Deviation** | | | **Fz - Cz** | | | | **Fz - Pz** | | | | **Cz - Pz** | | | |
|---|---|---|---|---|---|---|---|---|---|---|---|---|---|---|---|
| | **Fz** | **Cz** | **Pz** | **r** | **t** | **p** | **d** | **r** | **t** | **p** | **d** | **r** | **t** | **p** | **d** |
| **Ready** | 0.121 ± 0.03 | 0.120 ± 0.03 | 0.010 ± 0.02 | .857 | 0.589 | .559 | 0.093 | **.436** | **4.708** | **<.0005** | **0.744** | **.561** | **5.282** | **<.0005** | **0.835** |
| **Call Sign** | 0.107 ± 0.02 | 0.105 ± 0.02 | 0.091 ± 0.01 | .811 | 0.829 | .412 | 0.131 | **.418** | **5.522** | **<.0005** | **0.873** | **.594** | **7.129** | **<.0005** | **1.127** |
| **Go** | 0.103 ± 0.02 | 0.100 ± 0.02 | 0.084 ± 0.01 | .789 | 1.356 | .183 | 0.214 | **.509** | **6.207** | **<.0005** | **0.981** | **.708** | **7.077** | **<.0005** | **1.119** |
| **To** | 0.089 ± 0.01 | 0.090 ± 0.01 | 0.081 ± 0.01 | .741 | 0.557 | .581 | 0.088 | **.453** | **4.710** | **<.0005** | **0.745** | **.639** | **5.413** | **<.0005** | **0.856** |
| **Color** | 0.096 ± 0.02 | 0.098 ± 0.02 | 0.086 ± 0.01 | .767 | 0.905 | .371 | 0.143 | **.501** | **3.815** | **<.0005** | **0.603** | **.843** | **8.101** | **<.0005** | **1.281** |
| **Number** | 0.086 ± 0.02 | 0.083 ± 0.02 | 0.077 ± 0.01 | .669 | 1.426 | .162 | 0.225 | **.383** | **3.766** | **<.0005** | **0.595** | **.690** | **3.699** | **<.0005** | **0.585** |
| **Now** | 0.084 ± 0.01 | 0.082 ± 0.01 | 0.078 ± 0.01 | .673 | 1.326 | .192 | 0.210 | **.528** | **4.866** | **<.0005** | **0.769** | **.634** | **4.378** | **<.0005** | **0.692** |

**Supplementary Table 2.** Electrode by word mean + 1 standard deviation and post hoc tests. Bonferroni-corrected significant effects are indicated in bold numbers. Degrees of freedom for all tests equals 39.

### 1.1.3 Electrode by Frequency Band Interaction

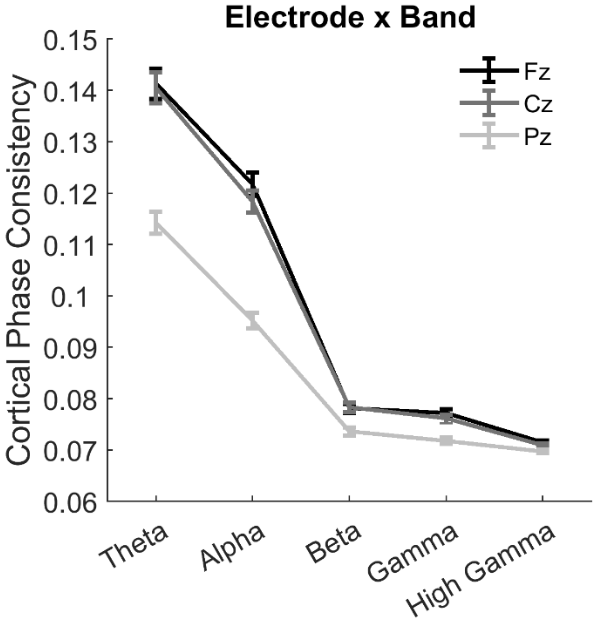


**Supplementary Figure 3.** Electrode by frequency band interaction plot. In this figure, cortical phase consistency is on the y axis, while frequency band is on the x axis. Fz is plotted in black, Cz is plotted in dark gray, and Pz is plotted in light gray. Collapsing across listening condition, language group, and word, there was a phase consistency difference across electrodes that varied by frequency band. This was driven by Fz and Cz having greater phase consistency than Pz, especially over the lower frequency bands (see Supplementary Table 3).

| | Mean ± 1 Standard Deviation | | | Fz - Cz | | | | Fz - Pz | | | | Cz - Pz | | | |
|---|---|---|---|---|---|---|---|---|---|---|---|---|---|---|---|
| | **Fz** | **Cz** | **Pz** | **r** | **t** | **p** | **d** | **r** | **t** | **p** | **d** | **r** | **t** | **p** | **d** |
| **Theta** | 0.141 ± 0.04 | 0.140 ± 0.03 | 0.114 ± 0.02 | .843 | 0.271 | .788 | 0.042 | **.580** | **5.804** | **<.0005** | **0.918** | **.782** | **8.045** | **<.0005** | **1.272** |
| **Alpha** | 0.122 ± 0.03 | 0.118 ± 0.02 | 0.095 ± 0.01 | .845 | 1.471 | .149 | 0.233 | **.592** | **7.648** | **<.0005** | **1.209** | **.778** | **10.289** | **<.0005** | **1.627** |
| **Beta** | 0.078 ± 0.01 | 0.078 ± 0.01 | 0.074 ± 0.01 | .727 | 0.223 | .825 | 0.035 | **.422** | **3.565** | **.001** | **0.564** | **.663** | **4.282** | **<.0005** | **0.677** |
| **Gamma** | 0.077 ± 0.01 | 0.076 ± 0.01 | 0.072 ± 0.01 | .654 | 1.237 | .224 | 0.196 | **.524** | **5.593** | **<.0005** | **0.884** | **.732** | **5.826** | **<.0005** | **0.921** |
| **High Gamma** | 0.071 ± 0.01 | 0.076 ± 0.01 | 0.070 ± 0.01 | .626 | 1.000 | .323 | 0.158 | **.538** | **3.151** | **.003** | **0.498** | .770 | 2.503 | .017 | 0.396 |

**Supplementary Table 3.** Electrode by frequency band mean ± 1 standard deviation and post hoc tests. Bonferroni-corrected significant effects are indicated in bold numbers. Degrees of freedom for all tests equals 39.

### 1.1.4 Word by Frequency Band Interaction

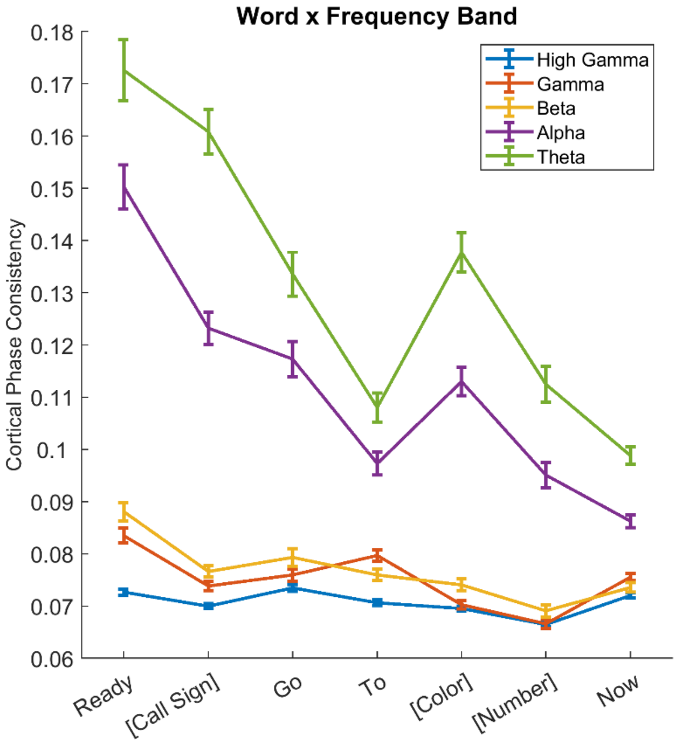


**Supplementary Figure 4.** Electrode by frequency band interaction plot. In this figure, cortical phase consistency is on the y axis, while word is on the x axis. Frequency bands are plotted in different colors as indicated by the legend. Collapsing across listening condition, electrode, and language group, there was a phase consistency difference across words and frequency bands. This was driven by greater phase consistency over theta and alpha bands, especially over the early words and the color. ‘To’ also showed phase consistency differences between the higher-frequency bands (see Supplementary Tables 4 and 5).

| | **Theta** | **Alpha** | **Beta** | **Gamma** | **High Gamma** |
|---|---|---|---|---|---|
| **Ready** | 0.173 ± 0.06 | 0.150 ± 0.04 | 0.088 ± 0.02 | 0.084 + 0.01 | 0.073 ± 0.005 |
| **Call Sign** | 0.161 ± 0.04 | 0.123 ± 0.03 | 0.077 ± 0.01 | 0.074 + 0.008 | 0.070 ± 0.004 |
| **Go** | 0.134 ± 0.04 | 0.117 ± 0.03 | 0.079 ± 0.02 | 0.076 + 0.01 | 0.073 ± 0.007 |
| **To** | 0.108 ± 0.03 | 0.097 ± 0.02 | 0.076 ± 0.01 | 0.080 + 0.01 | 0.071 ± 0.005 |
| **Color** | 0.138 ± 0.04 | 0.113 ± 0.03 | 0.074 ± 0.01 | 0.070 + 0.007 | 0.070 ± 0.004 |
| **Number** | 0.112 ± 0.03 | 0.095 ± 0.02 | 0.069 ± 0.01 | 0.067 + 0.006 | 0.066 ± 0.004 |
| **Now** | 0.099 ± 0.02 | 0.086 ± 0.01 | 0.074 ± 0.01 | 0.076 + 0.007 | 0.072 ± 0.006 |

**Supplementary Table 4.** Mean ± 1 standard deviation for each word in each frequency band.

| | Theta v. Alpha | | | | Theta v. Beta | | | | Theta V. Gamma | | | |
|---|---|---|---|---|---|---|---|---|---|---|---|---|
| | r | t | p | d | r | t | p | d | r | t | p | d |
| Ready | **0.903** | **4.470** | **<.0005** | **0.707** | **0.102** | **8.197** | **<.0005** | **1.296** | **0.132** | **8.763** | **<.0005** | **1.386** |
| Call Sign | **0.885** | **12.136** | **<.0005** | **1.919** | **0.322** | **14.339** | **<.0005** | **2.267** | **0.027** | **13.866** | **<.0005** | **2.192** |
| Go | **0.844** | **4.758** | **<.0005** | **0.752** | **0.178** | **8.369** | **<.0005** | **1.323** | **0.243** | **9.293** | **<.0005** | **1.469** |
| To | **0.881** | **5.042** | **<.0005** | **0.797** | **0.181** | **7.603** | **<.0005** | **1.202** | **0.075** | **6.396** | **<.0005** | **1.011** |
| Color | **0.902** | **7.380** | **<.0005** | **1.167** | **0.248** | **9.538** | **<.0005** | **1.508** | **0.127** | **9.868** | **<.0005** | **1.560** |
| Number | **0.922** | **7.720** | **<.0005** | **1.221** | **0.114** | **8.101** | **<.0005** | **1.281** | **0.065** | **8.635** | **<.0005** | **1.365** |
| Now | **0.833** | **7.836** | **<.0005** | **1.239** | **0.057** | **8.098** | **<.0005** | **1.280** | **0.092** | **7.974** | **<.0005** | **1.261** |

| | Theta v. High Gamma | | | | Alpha v. Beta | | | | Alpha v. Gamma | | | |
|---|---|---|---|---|---|---|---|---|---|---|---|---|
| | r | t | p | d | r | t | p | d | r | t | p | d |
| Ready | **0.003** | **9.755** | **<.0005** | **1.542** | **0.158** | **8.929** | **<.0005** | **1.412** | **0.225** | **9.964** | **<.0005** | **1.575** |
| Call Sign | **-0.004** | **14.581** | **<.0005** | **2.305** | **0.342** | **11.421** | **<.0005** | **1.806** | **0.096** | **11.255** | **<.0005** | **1.780** |
| Go | **0.208** | **9.620** | **<.0005** | **1.521** | **0.220** | **6.934** | **<.0005** | **1.096** | **0.392** | **8.338** | **<.0005** | **1.318** |
| To | **0.025** | **8.697** | **<.0005** | **1.375** | **0.181** | **6.935** | **<.0005** | **1.096** | **0.185** | **5.587** | **<.0005** | **0.883** |
| Color | **0.010** | **9.869** | **<.0005** | **1.561** | **0.221** | **8.454** | **<.0005** | **1.337** | **0.104** | **9.124** | **<.0005** | **1.443** |
| Number | **-0.076** | **8.576** | **<.0005** | **1.356** | **0.172** | **6.509** | **<.0005** | **1.029** | **0.101** | **7.202** | **<.0005** | **1.139** |
| Now | **-0.032** | **9.035** | **<.0005** | **1.429** | **-0.031** | **5.092** | **<.0005** | **0.805** | **0.054** | **4.955** | **<.0005** | **0.783** |

| | Alpha v. High Gamma | | | | Beta v. Gamma | | | |
|---|---|---|---|---|---|---|---|---|
| | r | t | p | d | r | t | p | d |
| Ready | **0.058** | **11.351** | **<.0005** | **1.795** | 0.295 | 1.490 | .144 | 0.236 |
| Call Sign | **-0.046** | **12.043** | **<.0005** | **1.904** | 0.044 | 1.407 | .167 | 0.222 |
| Go | **0.326** | **8.542** | **<.0005** | **1.351** | 0.614 | 1.582 | .122 | 0.250 |
| To | **-0.084** | **8.374** | **<.0005** | **1.324** | 0.356 | -2.172 | .036 | 0.343 |
| Color | **-0.039** | **9.183** | **<.0005** | **1.452** | 0.221 | 2.107 | .042 | 0.333 |
| Number | **-0.012** | **7.185** | **<.0005** | **1.136** | -0.169 | 1.147 | .258 | 0.181 |
| Now | **-0.055** | **6.619** | **<.0005** | **1.046** | 0.505 | -1.436 | .159 | 0.164 |

| | Beta v. High Gamma | | | | Gamma v. High Gamma | | | |
|---|---|---|---|---|---|---|---|---|
| | r | t | p | d | r | t | p | d |
| Ready | **0.025** | **5.294** | **<.0005** | **0.837** | **0.414** | **5.191** | **<.0005** | **0.821** |
| Call Sign | **0.116** | **3.901** | **<.0005** | **0.617** | 0.233 | 3.126 | .003 | 0.494 |
| Go | 0.366 | 2.320 | .026 | 0.367 | 0.381 | 1.374 | .177 | 0.217 |
| To | **0.171** | **3.679** | **<.0005** | **0.582** | **0.052** | **5.096** | **<.0005** | **0.806** |
| Color | 0.118 | 2.617 | .013 | 0.414 | 0.216 | 0.661 | .512 | 0.105 |
| Number | 0.196 | 1.523 | .136 | 0.241 | 0.279 | 0.149 | .883 | 0.024 |
| Now | 0.592 | 1.230 | .226 | 0.194 | 0.425 | 3.205 | .003 | 0.507 |

**Supplementary Table 5.** Word by frequency band post hoc tests. Bonferroni-corrected significant effects are indicated in bold numbers. Degrees of freedom for all tests equals 39. For ease of viewing, the table has been broken into 4 sections.

### 1.1.5 Frequency Band by Language Group Interaction

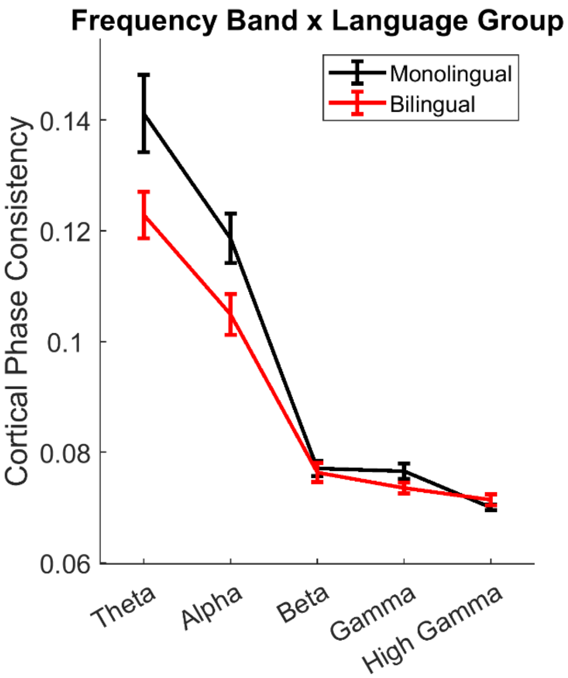


**Supplementary Figure 5.** Frequency band by language group interaction plot. In this figure, cortical phase consistency is on the y axis, and frequency band is on the x axis. Monolinguals are plotted in black and bilinguals in red. Collapsing across listening condition, electrode, and word, phase consistency for monolinguals and bilinguals differed in some frequency bands. Consistent with the language group main effects for specific bands reported in the main text, monolinguals had greater cortical phase consistency that was specific to theta and alpha frequency bands. There was a trending difference at gamma, with monolinguals having higher phase consistency over this band. (see Supplementary Table 6).

| | **Monolingual** | **Bilingual** | **F** | **p** | **$\eta_p^2$** |
|---|---|---|---|---|---|
| **Theta** | **0.141 ± 0.031** | **0.123 ± 0.019** | **5.113** | **.030** | **.119** |
| **Alpha** | **0.119 ± 0.020** | **0.105 ± 0.017** | **5.582** | **.023** | **.128** |
| **Beta** | 0.077 ± 0.006 | 0.076 ± 0.008 | 0.119 | .732 | .003 |
| **Gamma** | 0.077 ± 0.006 | 0.074 ± 0.005 | 3.170 | .083 | .077 |
| **High Gamma** | 0.070 ± 0.002 | 0.071 ± 0.004 | 1.595 | .214 | .040 |

**Supplementary Table 6.** Frequency band by language group mean + 1 standard deviation and ANOVAs within each frequency band. Degrees of freedom are 1, 38. Significant effects are bolded.

### 1.1.6 Listening Condition by Electrode by Frequency Band Interaction

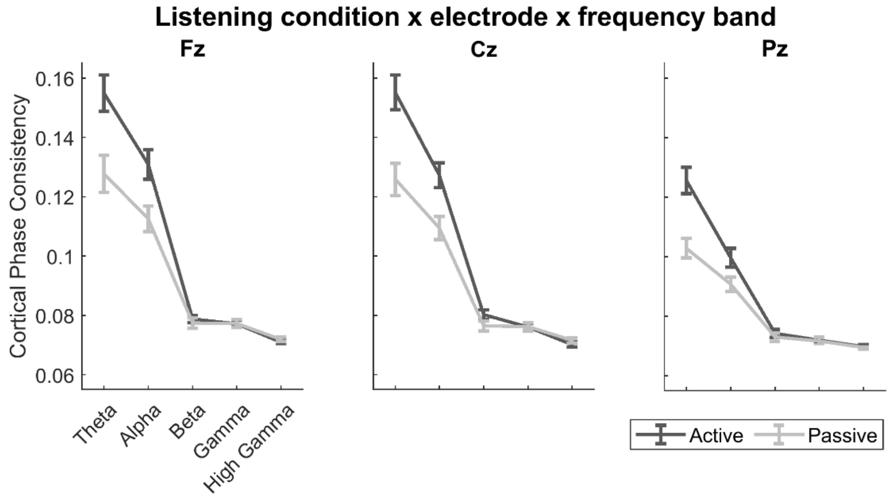


**Supplementary Figure 6.** Listening condition by electrode by frequency band. In this figure, cortical phase consistency is on the y axis, and frequency band is on the x axis. Active is plotted in dark gray and passive in light gray. From left to right, the plots show cortical consistency at Fz, Cz, and Pz. The y scale is consistent across the three plots. The effects were similar to the main effect and 2-way interactions described previously. Specifically, the greatest consistency was found for theta during active listening over Fz and Cz, as these channels did not differ from one another in their consistency ($t(39) = 0.049$, $p = .961$) but were higher than Fz and Cz consistency during passive listening and Pz consistency during both active and passive listening (all t(39)'s $\geq$ 4.622, p's < .0001). The difference between active and passive listening was significant for theta at Fz ($t(39) = 5.735$, $p < .0001$, $d = .906$), Cz ($t(39) = 5.809$, $p < .0001$, $d = .918$), and Pz ($t(39) = 5.046$, $p < .0001$, $d = .796$) and for alpha at Fz ($t(39) = 5.025$, $p < .0001$, $d = .794$), Cz ($t(39) = 4.359$, $p < .0001$, $d = .693$), while the other active/passive listening differences were not significant (all t(39)'s $\leq$ 2.633, p's $\geq$ .012). For means and standard deviations of each measure, see Supplementary Table 7.

| | **Active** | | | **Passive** | | |
|---|---|---|---|---|---|---|
| | **Fz** | **Cz** | **Pz** | **Fz** | **Cz** | **Pz** |
| **Theta** | 0.155 ± 0.04 | 0.155 ± 0.04 | 0.126 ± 0.03 | 0.128 ± 0.04 | 0.126 ± 0.03 | 0.103 ± 0.02 |
| **Alpha** | 0.131 ± 0.03 | 0.127 ± 0.03 | 0.100 ± 0.02 | 0.113 ± 0.03 | 0.109 ± 0.02 | 0.091 ± 0.02 |
| **Beta** | 0.079 ± 0.01 | 0.080 ± 0.01 | 0.074 ± 0.01 | 0.077 ± 0.01 | 0.076 ± 0.01 | 0.073 ± 0.01 |
| **Gamma** | 0.077 ± 0.01 | 0.076 ± 0.01 | 0.072 ± 0.01 | 0.077 ± 0.01 | 0.076 ± 0.01 | 0.072 ± 0.01 |
| **High Gamma** | 0.071 ± 0.005 | 0.070 ± 0.006 | 0.070 + 0.004 | 0.072 + 0.005 | 0.072 + 0.006 | 0.070 + 0.004 |

**Supplementary Table 7.** Listening condition by electrode by frequency band mean ± 1 standard deviation for each measure.

## 1.1.7 Listening Condition by Frequency Band by Word Interaction

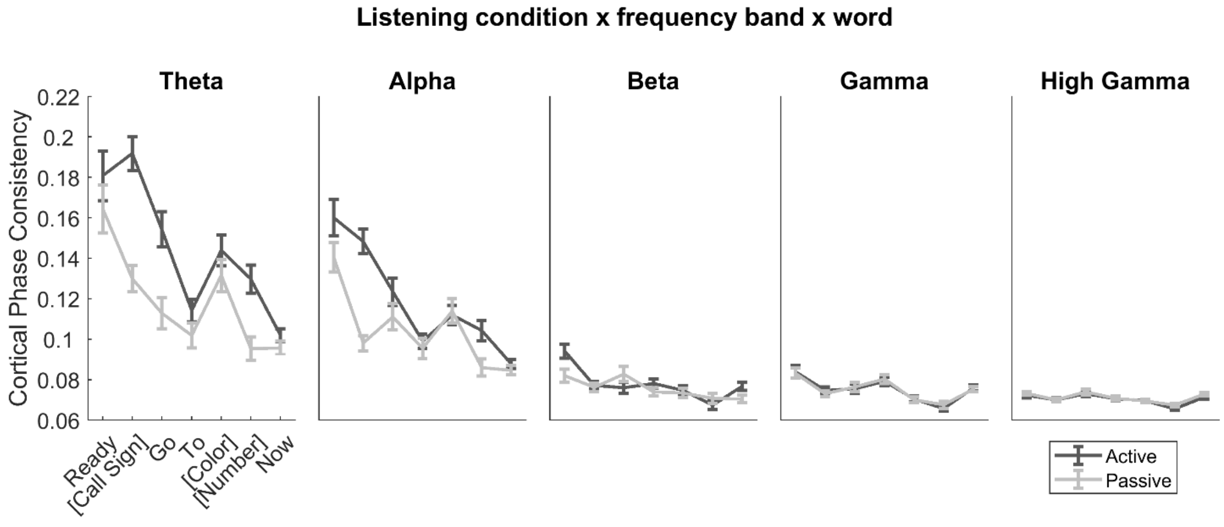


**Supplementary Figure 7.** Listening condition by frequency band by word interaction. Cortical phase consistency is on the y axis and word on the x axis, with active plotted in dark gray and passive in light gray. From left to right, theta, alpha, beta, gamma, and high gamma are plotted. The y scale is consistent across the three plots. Effects were similar to those described above. Theta differed between active and passive over the call sign ($t(39) = 7.52$, $p < .0014$, $d = 1.189$), go, ($t(39) = 4.036$, $p < .0014$, $d = .639$), and number ($t(39) = 4.736$, $p < .0014$, $d = .750$), alpha differed between active and passive over the call sign ($t(39) = 9.277$, $p < .0014$, $d = 1.465$) and number ($t(39) = 3.756$, $p < .0014$, $d = .594$), and beta differed between active and passive at ready ($t(39) = 3.457$, $p < .0014$, $d = .546$). No other differences were significant (all t(39)'s $\leq$ 2.167, p's $\geq$ .036).

| | | Theta | Alpha | Beta | Gamma | High Gamma |
|---|---|---|---|---|---|---|
| **Ready** | **Active** | 0.181 ± 0.08 | 0.160 ± 0.06 | 0.094 ± 0.02 | 0.084 ± 0.02 | 0.072 ± 0.01 |
| | **Passive** | 0.164 ± 0.08 | 0.140 ± 0.05 | 0.082 ± 0.02 | 0.083 ± 0.02 | 0.073 ± 0.01 |
| **CallSign** | **Active** | 0.192 ± 0.05 | 0.148 ± 0.04 | 0.077 ± 0.01 | 0.075 ± 0.01 | 0.070 ± 0.01 |
| | **Passive** | 0.130 ± 0.04 | 0.098 ± 0.02 | 0.076 ± 0.01 | 0.073 ± 0.01 | 0.070 ± 0.01 |
| **Go** | **Active** | 0.154 ± 0.05 | 0.124 ± 0.04 | 0.076 ± 0.02 | 0.075 ± 0.01 | 0.073 ± 0.01 |
| | **Passive** | 0.113 ± 0.05 | 0.111 ± 0.04 | 0.083 ± 0.02 | 0.076 ± 0.01 | 0.074 ± 0.01 |
| **To** | **Active** | 0.114 ± 0.04 | 0.099 ± 0.02 | 0.078 ± 0.01 | 0.079 ± 0.01 | 0.071 ± 0.01 |
| | **Passive** | 0.102 ± 0.04 | 0.096 ± 0.03 | 0.074 ± 0.01 | 0.080 ± 0.01 | 0.071 ± 0.01 |
| **Color** | **Active** | 0.144 ± 0.05 | 0.112 ± 0.03 | 0.075 ± 0.01 | 0.070 ± 0.01 | 0.070 ± 0.01 |
| | **Passive** | 0.132 ± 0.05 | 0.114 ± 0.04 | 0.073 ± 0.01 | 0.070 ± 0.01 | 0.069 ± 0.01 |
| **Number** | **Active** | 0.130 ± 0.04 | 0.104 ± 0.03 | 0.067 ± 0.01 | 0.066 ± 0.01 | 0.066 ± 0.01 |
| | **Passive** | 0.095 ± 0.04 | 0.086 ± 0.03 | 0.071 ± 0.01 | 0.068 ± 0.01 | 0.067 ± 0.01 |
| **Now** | **Active** | 0.102 ± 0.02 | 0.088 ± 0.01 | 0.077 ± 0.01 | 0.076 ± 0.01 | 0.071 ± 0.01 |
| | **Passive** | 0.096 ± 0.02 | 0.085 ± 0.01 | 0.070 ± 0.01 | 0.075 ± 0.01 | 0.073 ± 0.01 |

**Supplementary Table 8.** Listening condition by frequency band by word mean ± 1 standard deviation for each measure.

### 1.1.8 Electrode by Frequency Band by Language Group

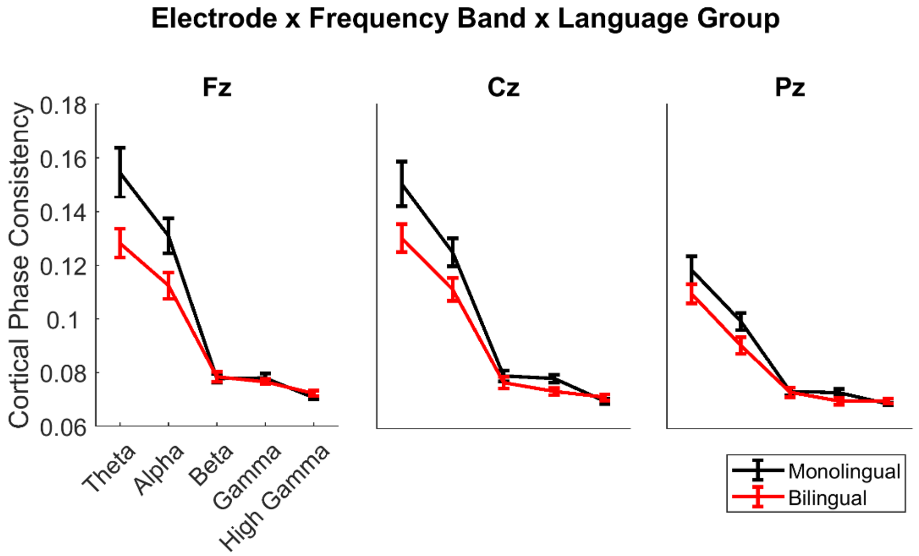


**Supplementary Figure 8.** Electrode by frequency band by language group interaction. Cortical phase consistency is on the y axis, and frequency band is on the x axis, with monolinguals plotted in black and bilinguals in red. From left to right, Fz, Cz, and Pz electrodes are plotted. The y scale is consistent across the three plots. The effects were similar to those described previously. Specifically, relative to bilinguals, monolinguals had greater theta consistency over Fz ($F(1, 38) = 5.896$, $p = .02$, $\eta_p^2 = .134$) and Cz ($F(1, 38) = 4.235$, $p = .047$, $\eta_p^2 = .100$), as well as greater alpha consistency over Fz ($F(1, 38) = 5.199$, $p = .028$, $\eta_p^2 = .120$), Cz, ($F(1, 38) = 4.174$, $p = .048$, $\eta_p^2 = .099$), and Pz ($F(1, 38) = 4.147$, $p = .049$, $\eta_p^2 = .098$), and greater gamma consistency over Cz ($F(1, 38) = 5.657$, $p = .023$, $\eta_p^2 = .130$). None of the other differences were significant (all F's $\leq$ 2.719, all p's $\geq$ .107).

| | | Monolingual | Bilingual |
|---|---|---|---|
| **Theta** | **Fz** | 0.154 ± 0.042 | 0.128 ± 0.024 |
| | **Cz** | 0.151 ± 0.037 | 0.130 ± 0.023 |
| | **Pz** | 0.119 ± 0.023 | 0.110 ± 0.016 |
| **Alpha** | **Fz** | 0.131 ± 0.029 | 0.112 ± 0.022 |
| | **Cz** | 0.125 ± 0.023 | 0.111 ± 0.019 |
| | **Pz** | 0.100 ± 0.014 | 0.091 ± 0.014 |
| **Beta** | **Fz** | 0.078 ± 0.007 | 0.078 ± 0.008 |
| | **Cz** | 0.080 ± 0.009 | 0.077 ± 0.010 |
| | **Pz** | 0.074 ± 0.006 | 0.073 ± 0.008 |
| **Gamma** | **Fz** | 0.078 ± 0.008 | 0.077 ± 0.005 |
| | **Cz** | 0.079 ± 0.007 | 0.074 ± 0.006 |
| | **Pz** | 0.073 ± 0.006 | 0.070 ± 0.006 |
| **High Gamma** | **Fz** | 0.071 ± 0.003 | 0.072 ± 0.005 |
| | **Cz** | 0.070 ± 0.004 | 0.072 ± 0.005 |
| | **Pz** | 0.069 ± 0.002 | 0.070 ± 0.004 |

**Supplementary Table 9.** Electrode by frequency band by language group means ± 1 standard deviation.

### 1.1.9 Electrode by Word by Frequency Band

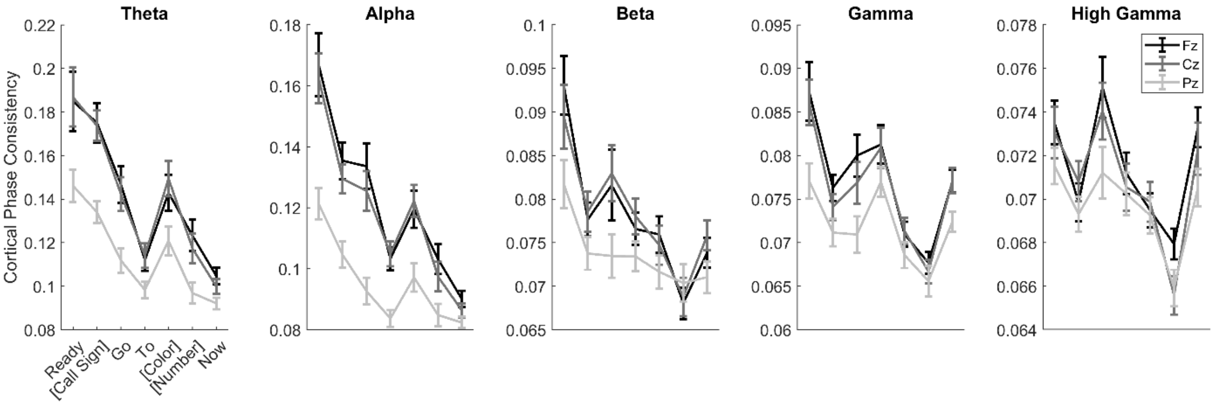


**Supplementary Figure 9.** Electrode by word by frequency band interaction. Cortical phase consistency is on the y axis, and word is on the x axis, with Fz plotted in black, Cz plotted in dark gray, and Pz plotted in light gray. From left to right, theta, alpha, beta, gamma, and high gamma frequency bands are plotted. The effects aligned with those previously described. To visualize the electrode and word differences within each frequency band, the plots for each band are individually scaled.

The greatest consistency was in response to ‘ready’ over the theta range at Fz and Cz, which did not differ from one another ($t(39) = 0.233$, $p = .817$, $d = .037$), and the difference from Pz theta over this word did not survive multiple corrections (Fz v. Pz: $t(39) = 3.145$, $p = .003$ $d = 0.497$; Cz v. Pz: $t(39) = 3.77$, $p = .0005$, $d = 0.596$). Theta consistency at Fz in response to ‘ready’ was greater than beta, gamma, and high gamma consistency to every word in the sentence at all three electrodes, as well as to every word except ‘ready’ for alpha activity at Pz, ‘to’, [color], [number], and ‘now’ for alpha activity over Cz and Fz, in response to ‘go’, ‘to’, [number], and ‘now’ for theta activity at Pz, ‘now’ for theta activity at Fz and Cz, as well as theta activity for ‘to’ over Fz (all $t(39)$’s $\geq 5.221$, all $p$’s $\leq .000006$, all $d$’s $\geq 0.826$). Theta consistency at Cz in response to ‘ready’ was greater than beta, gamma, and high gamma consistency to every word in the sentence at all three electrodes and for alpha consistency over Pz, for every word except ‘ready’ and [call sign] for alpha activity at Cz, for ‘to’, [color], [number], and ‘now’ for alpha activity at Fz, ‘go’, ‘to’, [number], and ‘now’ for theta activity at Pz, and ‘to’, [number], and ‘now’ for theta activity at Cz (all $t(39)$’s $\geq 5.175$, all $p$’s $\leq .000007$, all $d$’s $\geq 0.818$). All other comparisons between theta activity over ‘ready’ at Fz and Cz and the remaining words, bands, and electrodes were not significant (all $t(39)$’s $\leq 5.068$, all $p$’s $\geq .00001$, all $d$’s $\leq 0.801$).

Within the alpha band, consistency was greatest at Fz and Cz, which did not differ from one another ($t(39) = 0.754$, $p = .455$, $d = .120$). While Fz activity in the alpha band over ‘ready’ also did not differ from Pz over this word and band after correcting for multiple comparisons ($t(39) = 4.745$, $p = .00002$, $d = .750$), the difference between Cz and Pz was significant ($t(39) = 5.736$, $p = .000001$, $d = .907$). Fz and Cz alpha band consistency at each word in the sentence did not differ (all $t(39)$’s $\leq 1.813$, all $p$’s $\geq .078$, all $d$’s $\leq 0.287$). Differences in consistency between Fz and Pz over the alpha band were seen at [call sign], ‘to’, and ‘go’ (all $t(39)$’s $\geq 5.193$, all $p$’s $\leq .000007$, all $d$’s $\geq 0.821$). In addition to the difference between Cz and Pz at ‘ready’ over this band (reported above), differences

were seen between these electrodes over the response to [call sign], 'go', 'to' and 'color' (all t(39)'s ≥ 5.889,  all p's ≤ . 0000007, all d's ≥ 0.931).

Again, within the beta band, consistency was greatest in response to 'ready'. In this band, the three electrodes had equivalent consistency over this word (Fz v. Cz (t(39) = 1.443, p = .157, d = 0.228), Fz v. Pz (t(39) = 3.507, p = .001, d = 0.555), Cz v. Pz (t(39) = 2.446, p = .019, d = 0.387). Similar to the alpha activity, consistency did not differ between Fz and Cz over the remaining words in the sentence (all t(39)'s ≤ 1.364,  all p's ≥  . 180, all d's ≤ 0.216). However, neither Fz (all t(39)'s ≤ 1.971, all p's ≥ .056, all d's ≤ 0.312). or Cz (all t(39)'s ≤ 4.169, all p's ≥ . 0002, all d's ≤ 0.659) differed from Pz over the remaining words in the alpha band. The only differences in the beta band were seen between 'ready' at Fz and [number] and 'now' at Fz, [number] at Cz, and the [call sign], 'go', 'to', [color], [number], 'now' at Pz (all t(39)'s ≥ 5.197,  all p's ≤ .000007, all d's ≥ 0.822); between 'ready' at Cz and the [number] at Fz (t(39) = 5.3571, p = .000004, d = 0.847), Cz (t(39) = 5.981, p = .0000006, d = 0.946) and Pz (t(39) = 5.121, p = .000009, d = 0.810).

Within the gamma band, consistency was again greatest at 'ready', with the three electrodes not differing over this word (all t(39)'s ≤ 4.245, all p's ≥ .0001, all d's ≤ 0.671).  Differences in this band were seen between 'ready' at Fz and [number] at Fz (t(39) = 6.415, p = .0000001, d = 1.014), Cz (t(39) = 5.854, p =.0000008, d = 0.926), and Pz (t(39) = 6.066, p = .0000004, d = 0.959); 'ready' at Cz and the [color] and [number] at Cz, and every word except 'ready' and 'to' at Pz (all t(39)'s ≥ 5.225, all p's ≤ .000006, all d's ≥ 0.826); the [call sign] and [number] at Fz (t(39) = 5.183, p =.000007, d = 0.820); [number] at Pz and 'to', and 'now' at Cz as well as 'go', 'to' and 'now' at Fz (all t(39)'s ≥ 5.514, all p's ≤ .000002, all d's ≥ 0.872); [number] at Cz and 'to' at Fz (t(39) = 5.655, p =.000002, d = 0.894) and Cz (t(39) = 5.325, p = .000004, d = 0.842), and [number] at Fz and 'ready' and 'to' at Cz and 'now' at Fz (all t(39)'s ≥ 5.156,  all p's ≤ .000008, all d's ≥ 0.815).

Within the high gamma band, the only differences were seen in the consistency of [number] over Cz and 'go' at Cz, 'ready', 'go', and 'now' at Fz (all t(39)'s ≥ 6.328,  all p's ≤ . 0000002, all d's ≥ 1.001); [number] over Pz and 'go' at Fz and Cz as well as 'ready' and 'now' at Fz (all t(39)'s ≥ 5.245,  all p's ≤ . 000006, all d's ≥ .829); and between [number] and 'now' at Cz (t(39) = 5.273, p = .000005, d = 0.834) and Fz (t(39) = 5.308, p = .000005, d = 0.839).

| | | Theta | Alpha | Beta | Gamma | High Gamma |
|---|---|---|---|---|---|---|
| | **Ready** | 0.185 ± 0.087 | 0.167 ± 0.065 | 0.093 ± 0.021 | 0.087 ± 0.021 | 0.074 ± 0.006 |
| | **Call Sign** | 0.175 ± 0.058 | 0.135 ± 0.038 | 0.078 ± 0.012 | 0.076 ± 0.010 | 0.070 ± 0.006 |
| **Fz** | **Go** | 0.147 ± 0.054 | 0.134 ± 0.047 | 0.082 ± 0.026 | 0.080 ± 0.015 | 0.075 ± 0.009 |
| | **To** | 0.112 ± 0.031 | 0.103 ± 0.024 | 0.077 ± 0.012 | 0.081 ± 0.014 | 0.071 ± 0.006 |
| | **Color** | 0.143 ± 0.053 | 0.120 ± 0.039 | 0.076 ± 0.013 | 0.071 ± 0.009 | 0.069 ± 0.005 |
| | **Number** | 0.123 ± 0.046 | 0.103 ± 0.031 | 0.068 ± 0.011 | 0.068 ± 0.008 | 0.068 ± 0.004 |
| | **Now** | 0.105 ± 0.024 | 0.090 ± 0.017 | 0.074 ± 0.011 | 0.077 ± 0.008 | 0.073 ± 0.006 |

| | | Theta | Alpha | Beta | Gamma | High Gamma |
|---|---|---|---|---|---|---|
| | **Ready** | 0.187 ± 0.085 | 0.163 ± 0.052 | 0.089 ± 0.023 | 0.086 ± 0.017 | 0.073 ± 0.008 |
| | **Call Sign** | 0.174 ± 0.044 | 0.129 ± 0.030 | 0.079 ± 0.015 | 0.074 ± 0.009 | 0.071 ± 0.006 |
| **Cz** | **Go** | 0.142 ± 0.049 | 0.126 ± 0.041 | 0.083 ± 0.020 | 0.077 ± 0.015 | 0.074 ± 0.008 |
| | **To** | 0.114 ± 0.036 | 0.105 ± 0.026 | 0.078 ± 0.013 | 0.081 ± 0.014 | 0.071 ± 0.007 |
| | **Color** | 0.149 ± 0.052 | 0.122 ± 0.033 | 0.075 ± 0.014 | 0.071 ± 0.010 | 0.070 ± 0.006 |
| | **Number** | 0.117 ± 0.043 | 0.097 ± 0.031 | 0.069 ± 0.014 | 0.067 ± 0.009 | 0.066 ± 0.006 |
| | **Now** | 0.100 ± 0.022 | 0.086 ± 0.015 | 0.076 ± 0.011 | 0.077 ± 0.009 | 0.072 ± 0.008 |

| | | Theta | Alpha | Beta | Gamma | High Gamma |
|---|---|---|---|---|---|---|
| | **Ready** | 0.146 ± 0.047 | 0.121 ± 0.033 | 0.082 ± 0.018 | 0.077 ± 0.013 | 0.072 ± 0.005 |
| | **Call Sign** | 0.134 ± 0.031 | 0.105 ± 0.027 | 0.074 ± 0.012 | 0.071 ± 0.010 | 0.069 ± 0.005 |
| **Pz** | **Go** | 0.112 ± 0.035 | 0.093 ± 0.028 | 0.073 ± 0.016 | 0.071 ± 0.013 | 0.071 ± 0.007 |
| | **To** | 0.098 ± 0.023 | 0.084 ± 0.017 | 0.073 ± 0.011 | 0.077 ± 0.011 | 0.070 ± 0.006 |
| | **Color** | 0.121 ± 0.041 | 0.097 ± 0.030 | 0.072 ± 0.012 | 0.069 ± 0.010 | 0.069 ± 0.005 |
| | **Number** | 0.097 ± 0.030 | 0.085 ± 0.023 | 0.070 ± 0.013 | 0.066 ± 0.011 | 0.066 ± 0.005 |
| | **Now** | 0.092 ± 0.016 | 0.082 ± 0.012 | 0.071 ± 0.012 | 0.072 ± 0.007 | 0.071 ± 0.005 |

**Supplementary Table 10.** Electrode by word by frequency band means ± 1 standard deviation.

### 1.1.10 Listening Condition by Electrode by Word by Language Group

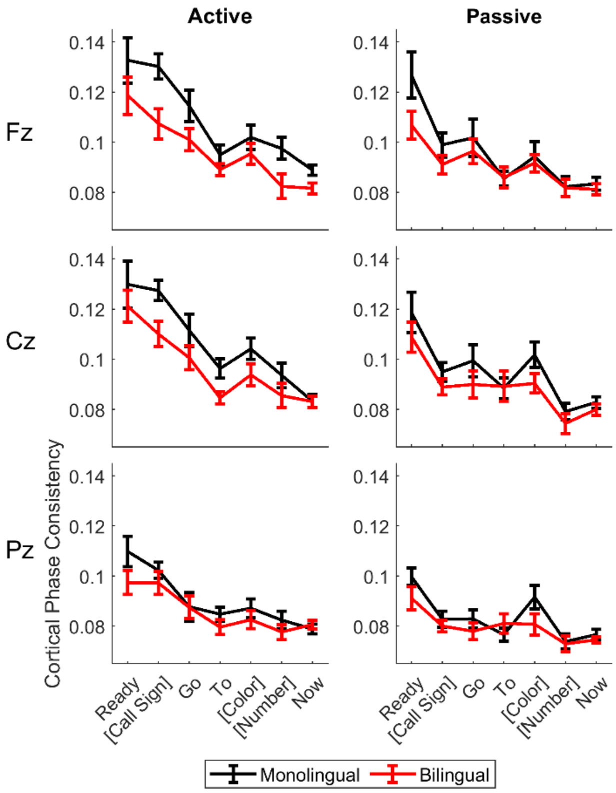


**Supplementary Figure 10.** Listening condition by electrode by word by language group interaction. Cortical phase consistency is on the y axis, and word is on the x axis, with monolinguals plotted in black and bilinguals in red. From left to right, active and passive conditions are plotted and from top to bottom, Fz, Cz, and Pz are plotted. The effects aligned with the main effects and 2- and 3-way interactions described above and in the main text. Due to the significant number of comparisons and the fact that they echoed what has been reported, no post hocs are provided for this 4-way interaction. See supplemental table 11 for means and standard deviations of the different measures.

| | | Active | | Passive | |
|---|---|---|---|---|---|
| | | **Monolingual** | **Bilingual** | **Monolingual** | **Bilingual** |
| **Ready** | **Fz** | 0.133 ± 0.041 | 0.119 ± 0.033 | 0.127 ± 0.041 | 0.107 ± 0.025 |
| | **Cz** | 0.130 ± 0.022 | 0.107 ± 0.027 | 0.099 ± 0.022 | 0.091 ± 0.017 |
| | **Pz** | 0.114 ± 0.028 | 0.101 ± 0.020 | 0.102 ± 0.033 | 0.096 ± 0.022 |
| **Call Sign** | **Fz** | 0.095 ± 0.018 | 0.089 ± 0.010 | 0.085 ± 0.013 | 0.086 ± 0.018 |
| | **Cz** | 0.102 ± 0.022 | 0.095 ± 0.019 | 0.094 ± 0.027 | 0.092 ± 0.016 |
| | **Pz** | 0.098 ± 0.020 | 0.082 ± 0.022 | 0.082 ± 0.018 | 0.082 ± 0.016 |
| **Go** | **Fz** | 0.089 ± 0.009 | 0.082 ± 0.010 | 0.083 ± 0.012 | 0.081 ± 0.010 |
| | **Cz** | 0.130 ± 0.042 | 0.121 ± 0.028 | 0.119 ± 0.036 | 0.109 ± 0.027 |
| | **Pz** | 0.127 ± 0.018 | 0.110 ± 0.023 | 0.095 ± 0.016 | 0.089 ± 0.014 |
| **To** | **Fz** | 0.112 ± 0.029 | 0.101 ± 0.022 | 0.099 ± 0.028 | 0.090 ± 0.024 |
| | **Cz** | 0.096 ± 0.017 | 0.085 ± 0.011 | 0.088 ± 0.019 | 0.089 ± 0.027 |
| | **Pz** | 0.104 ± 0.019 | 0.094 ± 0.020 | 0.102 ± 0.023 | 0.090 ± 0.017 |
| **Color** | **Fz** | 0.094 ± 0.022 | 0.086 ± 0.021 | 0.079 ± 0.015 | 0.074 ± 0.018 |
| | **Cz** | 0.083 ± 0.012 | 0.083 ± 0.010 | 0.083 ± 0.010 | 0.080 ± 0.010 |
| | **Pz** | 0.110 ± 0.027 | 0.097 ± 0.022 | 0.100 ± 0.015 | 0.091 ± 0.020 |
| **Number** | **Fz** | 0.102 ± 0.014 | 0.097 ± 0.020 | 0.083 ± 0.014 | 0.080 ± 0.011 |
| | **Cz** | 0.088 ± 0.026 | 0.087 ± 0.020 | 0.083 ± 0.017 | 0.078 ± 0.015 |
| | **Pz** | 0.085 ± 0.013 | 0.080 ± 0.013 | 0.077 ± 0.012 | 0.081 ± 0.017 |
| **Now** | **Fz** | 0.087 ± 0.017 | 0.083 ± 0.016 | 0.092 ± 0.021 | 0.081 ± 0.019 |
| | **Cz** | 0.082 ± 0.016 | 0.078 ± 0.014 | 0.074 ± 0.014 | 0.073 ± 0.014 |
| | **Pz** | 0.079 ± 0.009 | 0.081 ± 0.008 | 0.077 ± 0.009 | 0.074 ± 0.005 |

**Supplementary Table 11.** Listening condition by electrode by word by language group means ± 1 standard deviation.

### 1.1.11 Listening Condition by Electrode by Word by Frequency Band

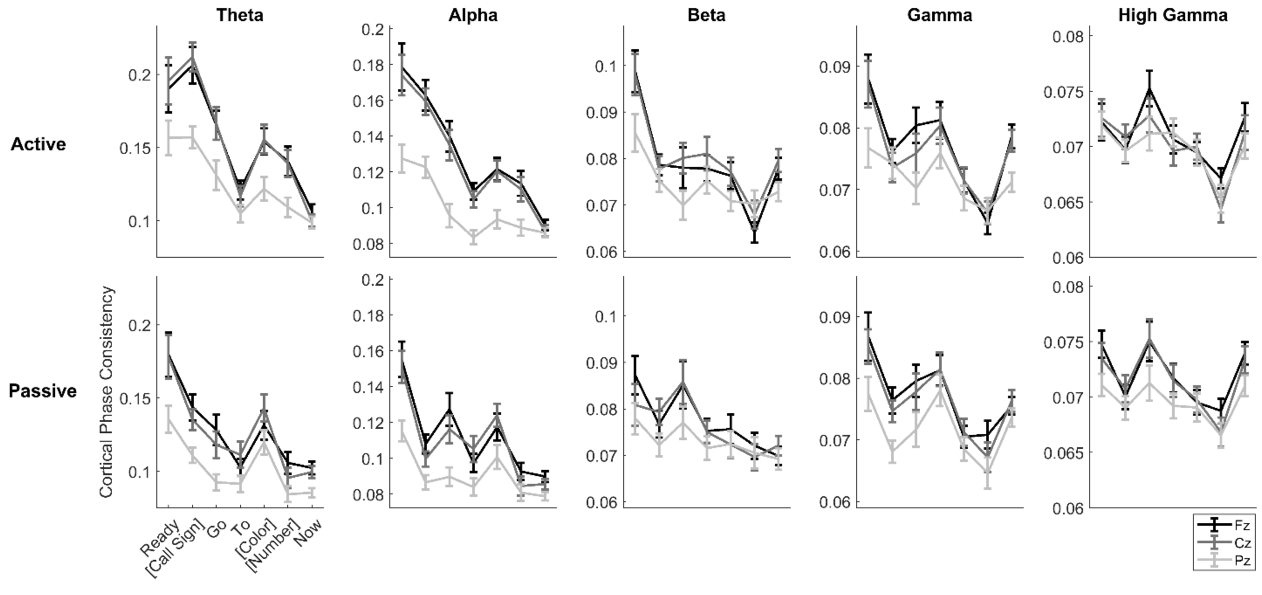


**Supplementary Figure 11.** Listening condition by electrode by word by frequency band interaction. Cortical phase consistency is on the y axis, and word is on the x axis, with Fz plotted in black, Cz plotted in dark gray, and Pz plotted in light gray. From left to right, theta, alpha, beta, gamma, and high gamma frequency bands are plotted. Active is plotted on the top row, and passive on the bottom row. To visualize the electrode and word differences within each frequency band and listening condition, the plots for each band are individually scaled. Within each frequency band, the active and passive plots are set to the same scale. Because this 4-way interaction yielded over 20,000 post-hoc comparisons with ~6800 significant after correcting for multiple comparisons, together with the fact that these effects aligned with those previously described (e.g., greatest consistency over theta and alpha for the earlier words at Fz and Cz), post hocs are not reported.

| | | Fz | | | | |
|---|---|---|---|---|---|---|
| | | **Theta** | **Alpha** | **Beta** | **Gamma** | **High Gamma** |
| **Ready** | **Active** | 0.190 ± 0.103 | 0.179 ± 0.084 | 0.099 ± 0.028 | 0.088 ± 0.025 | 0.072 ± 0.010 |
| | **Passive** | 0.180 ± 0.095 | 0.155 ± 0.063 | 0.087 ± 0.026 | 0.087 ± 0.025 | 0.075 ± 0.008 |
| **CallSign** | **Active** | 0.206 ± 0.078 | 0.163 ± 0.055 | 0.079 ± 0.014 | 0.076 ± 0.012 | 0.070 ± 0.008 |
| | **Passive** | 0.144 ± 0.056 | 0.108 ± 0.033 | 0.077 ± 0.018 | 0.076 ± 0.013 | 0.070 ± 0.007 |
| **Go** | **Active** | 0.165 ± 0.063 | 0.140 ± 0.054 | 0.078 ± 0.028 | 0.080 ± 0.018 | 0.075 ± 0.010 |
| | **Passive** | 0.128 ± 0.066 | 0.127 ± 0.057 | 0.085 ± 0.032 | 0.080 ± 0.016 | 0.075 ± 0.011 |
| **To** | **Active** | 0.121 ± 0.040 | 0.109 ± 0.030 | 0.078 ± 0.018 | 0.081 ± 0.019 | 0.071 ± 0.008 |
| | **Passive** | 0.103 ± 0.037 | 0.097 ± 0.032 | 0.075 ± 0.017 | 0.081 ± 0.016 | 0.072 ± 0.008 |
| **Color** | **Active** | 0.154 ± 0.056 | 0.122 ± 0.041 | 0.076 ± 0.018 | 0.071 ± 0.012 | 0.069 ± 0.006 |
| | **Passive** | 0.132 ± 0.062 | 0.117 ± 0.048 | 0.076 ± 0.020 | 0.071 ± 0.011 | 0.070 ± 0.007 |
| **Number** | **Active** | 0.141 ± 0.064 | 0.114 ± 0.045 | 0.064 ± 0.014 | 0.064 ± 0.011 | 0.067 ± 0.006 |
| | **Passive** | 0.106 ± 0.047 | 0.093 ± 0.030 | 0.072 ± 0.018 | 0.071 ± 0.014 | 0.069 ± 0.007 |
| **Now** | **Active** | 0.107 ± 0.026 | 0.090 ± 0.019 | 0.078 ± 0.015 | 0.079 ± 0.012 | 0.073 ± 0.008 |
| | **Passive** | 0.102 ± 0.028 | 0.090 ± 0.019 | 0.070 ± 0.013 | 0.076 ± 0.009 | 0.074 ± 0.006 |

**Cz**

| | | Theta | Alpha | Beta | Gamma | High Gamma |
|---|---|---|---|---|---|---|
| **Ready** | **Active** | 0.196 ± 0.101 | 0.174 ± 0.071 | 0.098 ± 0.028 | 0.087 ± 0.024 | 0.073 ± 0.011 |
| | **Passive** | 0.178 ± 0.095 | 0.151 ± 0.056 | 0.081 ± 0.028 | 0.085 ± 0.018 | 0.073 ± 0.009 |
| **CallSign** | **Active** | 0.212 ± 0.062 | 0.159 ± 0.047 | 0.078 ± 0.017 | 0.074 ± 0.015 | 0.071 ± 0.007 |
| | **Passive** | 0.135 ± 0.045 | 0.100 ± 0.027 | 0.079 ± 0.017 | 0.075 ± 0.011 | 0.071 ± 0.008 |
| **Go** | **Active** | 0.167 ± 0.071 | 0.135 ± 0.054 | 0.080 ± 0.021 | 0.076 ± 0.020 | 0.073 ± 0.010 |
| | **Passive** | 0.118 ± 0.058 | 0.116 ± 0.050 | 0.086 ± 0.030 | 0.078 ± 0.019 | 0.075 ± 0.011 |
| **To** | **Active** | 0.117 ± 0.043 | 0.105 ± 0.029 | 0.081 ± 0.023 | 0.080 ± 0.019 | 0.070 ± 0.008 |
| | **Passive** | 0.111 ± 0.057 | 0.105 ± 0.046 | 0.075 ± 0.016 | 0.081 ± 0.017 | 0.071 ± 0.009 |
| **Color** | **Active** | 0.156 ± 0.062 | 0.120 ± 0.038 | 0.077 ± 0.020 | 0.071 ± 0.014 | 0.070 ± 0.007 |
| | **Passive** | 0.143 ± 0.058 | 0.124 ± 0.041 | 0.072 ± 0.018 | 0.071 ± 0.014 | 0.070 ± 0.008 |
| **Number** | **Active** | 0.139 ± 0.059 | 0.110 ± 0.043 | 0.068 ± 0.019 | 0.066 ± 0.012 | 0.064 ± 0.007 |
| | **Passive** | 0.096 ± 0.045 | 0.084 ± 0.033 | 0.070 ± 0.018 | 0.067 ± 0.015 | 0.067 ± 0.008 |
| **Now** | **Active** | 0.100 ± 0.027 | 0.087 ± 0.020 | 0.080 ± 0.016 | 0.078 ± 0.011 | 0.071 ± 0.010 |
| | **Passive** | 0.099 ± 0.026 | 0.086 ± 0.019 | 0.072 ± 0.013 | 0.076 ± 0.011 | 0.073 ± 0.008 |

**Pz**

| | | Theta | Alpha | Beta | Gamma | High Gamma |
|---|---|---|---|---|---|---|
| **Ready** | **Active** | 0.157 ± 0.075 | 0.127 ± 0.049 | 0.086 ± 0.025 | 0.077 ± 0.020 | 0.072 ± 0.008 |
| | **Passive** | 0.136 ± 0.058 | 0.115 ± 0.036 | 0.078 ± 0.021 | 0.077 ± 0.018 | 0.071 ± 0.006 |
| **CallSign** | **Active** | 0.157 ± 0.048 | 0.123 ± 0.037 | 0.075 ± 0.016 | 0.074 ± 0.016 | 0.070 ± 0.006 |
| | **Passive** | 0.111 ± 0.032 | 0.087 ± 0.025 | 0.072 ± 0.016 | 0.068 ± 0.011 | 0.069 ± 0.007 |
| **Go** | **Active** | 0.131 ± 0.064 | 0.096 ± 0.041 | 0.070 ± 0.020 | 0.070 ± 0.016 | 0.071 ± 0.010 |
| | **Passive** | 0.092 ± 0.034 | 0.090 ± 0.033 | 0.077 ± 0.022 | 0.072 ± 0.018 | 0.071 ± 0.010 |
| **To** | **Active** | 0.105 ± 0.037 | 0.083 ± 0.025 | 0.075 ± 0.018 | 0.076 ± 0.016 | 0.071 ± 0.008 |
| | **Passive** | 0.091 ± 0.036 | 0.084 ± 0.030 | 0.072 ± 0.016 | 0.078 ± 0.016 | 0.069 ± 0.007 |
| **Color** | **Active** | 0.122 ± 0.050 | 0.094 ± 0.033 | 0.071 ± 0.013 | 0.069 ± 0.013 | 0.069 ± 0.007 |
| | **Passive** | 0.120 ± 0.053 | 0.101 ± 0.042 | 0.072 ± 0.018 | 0.069 ± 0.012 | 0.069 ± 0.008 |
| **Number** | **Active** | 0.109 ± 0.042 | 0.089 ± 0.029 | 0.070 ± 0.018 | 0.067 ± 0.013 | 0.065 ± 0.008 |
| | **Passive** | 0.084 ± 0.034 | 0.081 ± 0.029 | 0.071 ± 0.021 | 0.065 ± 0.016 | 0.067 ± 0.007 |
| **Now** | **Active** | 0.099 ± 0.025 | 0.086 ± 0.017 | 0.073 ± 0.013 | 0.071 ± 0.010 | 0.070 ± 0.007 |
| | **Passive** | 0.085 ± 0.020 | 0.079 ± 0.015 | 0.069 ± 0.015 | 0.074 ± 0.010 | 0.071 ± 0.006 |

**Supplementary Table 12.** Listening condition by electrode by word by frequency band means ± 1 standard deviation. Top table is Fz, middle table is Cz, bottom table is Pz.

### 1.1.12 Listening Condition by Electrode by Word by Frequency Band x Language Group

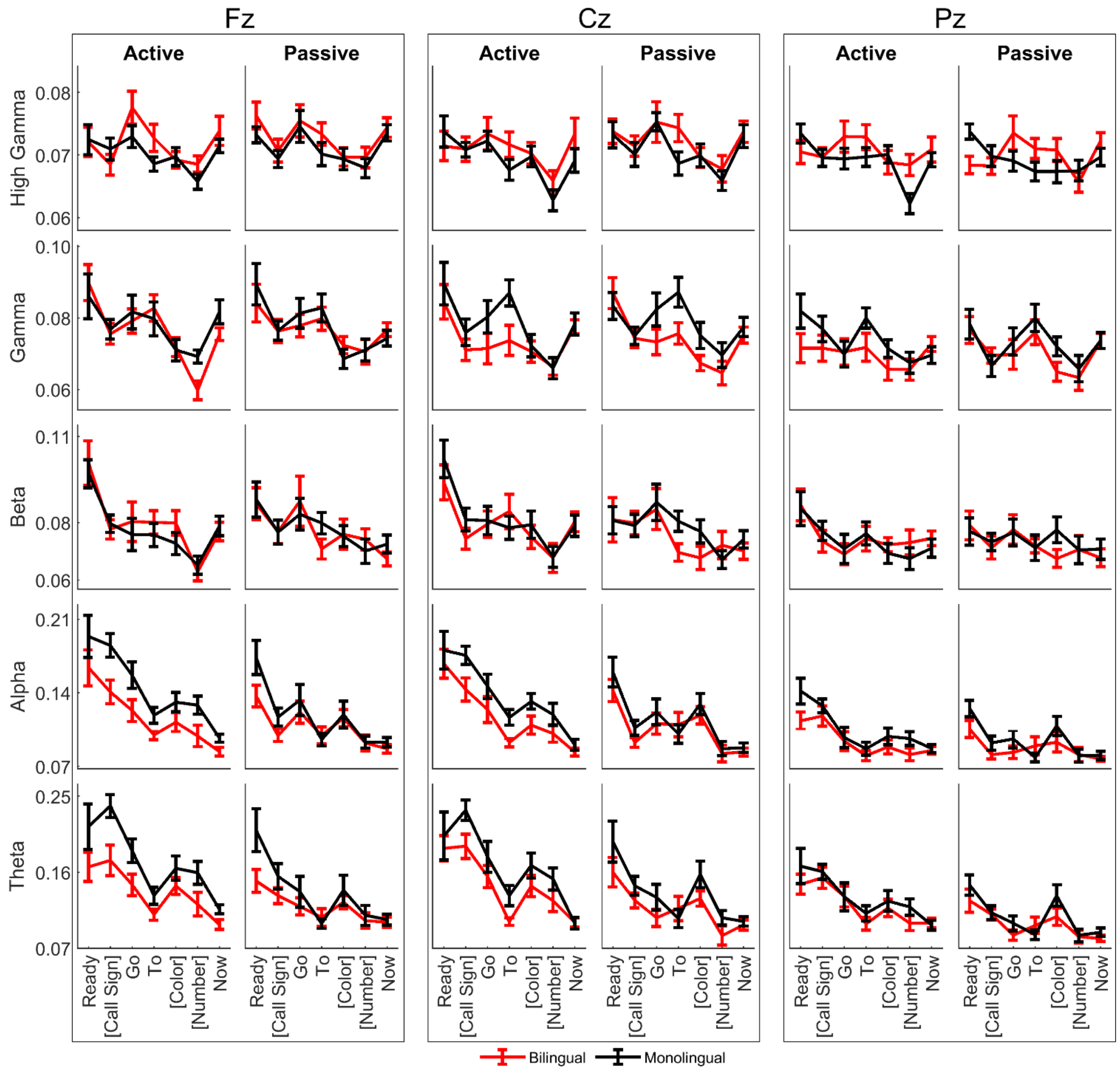


**Supplementary Figure 12.** Although the 5-way interaction was not significant, it is plotted here for visualization purposes. No statistics were run on these values. Cortical phase consistency is on the y axis, and word is on the x axis, with monolinguals plotted in black and bilinguals plotted in red. From left to right are plotted Fz Active, Fz passive, Cz active, Cz passive, Pz active an Pz passive. From bottom to top are plotted theta, alpha, beta, gamma, and high gamma frequency bands. Each band is plotted on its own y scale, as indicated by the y axis values on the leftmost plots.

## 1.2 Subcortical

All subcortical analyses were run only on words that were consistent across sentences (i.e., ‘ready’, ‘go’, ‘to’, and ‘now’). Stimulus refers to the pitch contour (male or female).

### 1.2.1 Word by Language Group Interaction

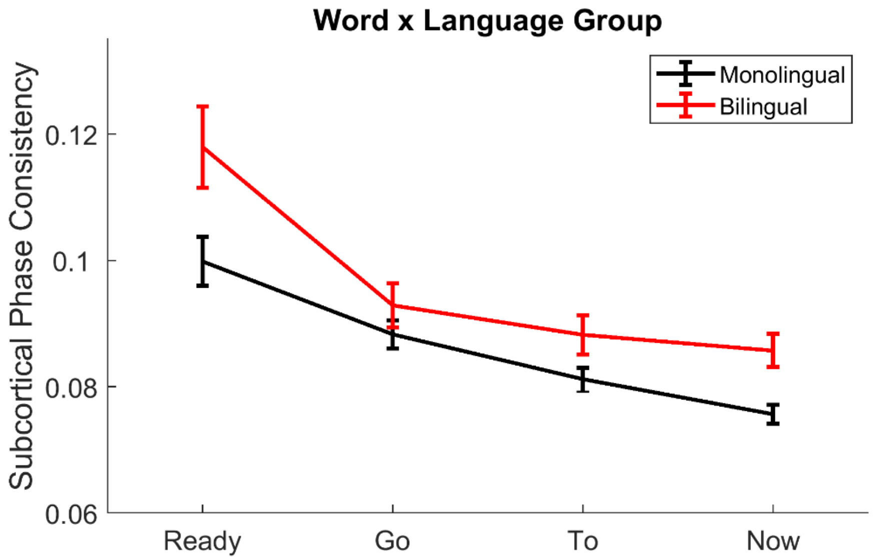


**Supplementary Figure 13.** Word by language group interaction. Subcortical phase consistency is on the y axis, and word is on the x axis, with monolinguals plotted in black and bilinguals plotted in red. These analyses show greater consistency for the bilingual response, particularly over ‘ready’ and ‘now’.

| | Monolingual | Bilingual | F(1, 38) | p | $\eta_p^2$ |
|---|---|---|---|---|---|
| **Ready** | **0.100 ± 0.017** | **0.118 ± 0.029** | **5.793** | **.021** | **0.132** |
| **Go** | 0.088 ± 0.010 | 0.093 ± 0.016 | 1.226 | .275 | 0.031 |
| **To** | 0.081 ± 0.008 | 0.088 ± 0.014 | 3.784 | .059 | 0.091 |
| **Now** | **0.076 ± 0.007** | **0.086 ± 0.012** | **10.980** | **.002** | **0.224** |

**Supplementary Table 13.** Word by language group means ± 1 standard deviation and ANOVAs for each word. Degrees of freedom are 1, 38.

### 1.2.2 Listening Condition by Stimulus (pitch contour) Interaction

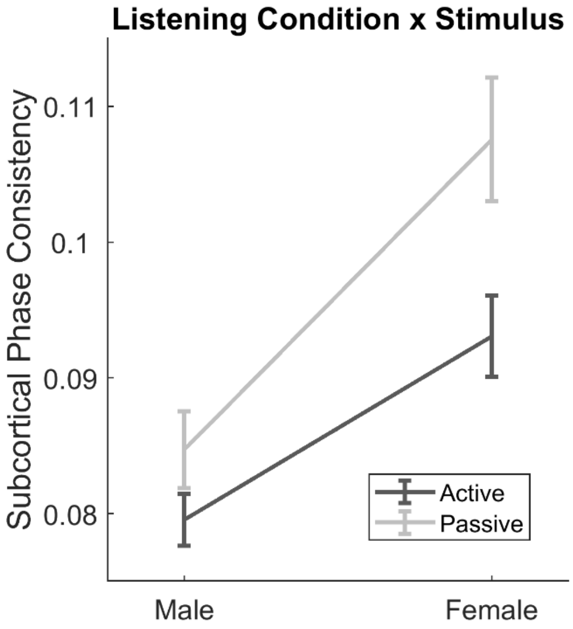


**Supplementary Figure 14.** Listening condition by stimulus (pitch contour) interaction. Subcortical phase consistency is plotted on the y-axis and stimulus (male pitch contour, left; female pitch contour, right) is plotted on the x-axis. Active is plotted in dark gray and passive is plotted in light gray. The passive condition shows greater response consistency than the active condition, with the consistency in response to the female during passive listening being higher than both the male and female pitch contours during active listening.

| | **Active** | **Passive** |
|---|---|---|
| **Male** | 0.080 ± 0.012 | 0.085 ± 0.018 |
| **Female** | 0.093 ± 0.019 | 0.108 ± 0.029 |

**Supplementary Table 14.** Listening condition by word means ± 1 standard deviation.

| **Comparison** | **r** | **t** | **p** | **d** |
|---|---|---|---|---|
| Male Active v. Male Passive | .051 | 1.548 | .13 | 0.245 |
| **Male Active v. Female Active** | **.734** | **6.609** | **<.0008** | **1.045** |
| **Male Active v. Female Passive** | **.034** | **5.752** | **<.0008** | **0.909** |
| Male Passive v. Female Active | -.069 | 1.964 | .057 | 0.31 |
| **Male Passive v. Female Passive** | **.799** | **8.028** | **<.0008** | **1.269** |
| **Female Active v. Female Passive** | **.100** | **2.796** | **<.0008** | **0.442** |

**Supplementary Table 15.** Listening condition by word post hoc tests. Degrees of freedom are 39.

### 1.2.3 Listening Condition by Word Interaction

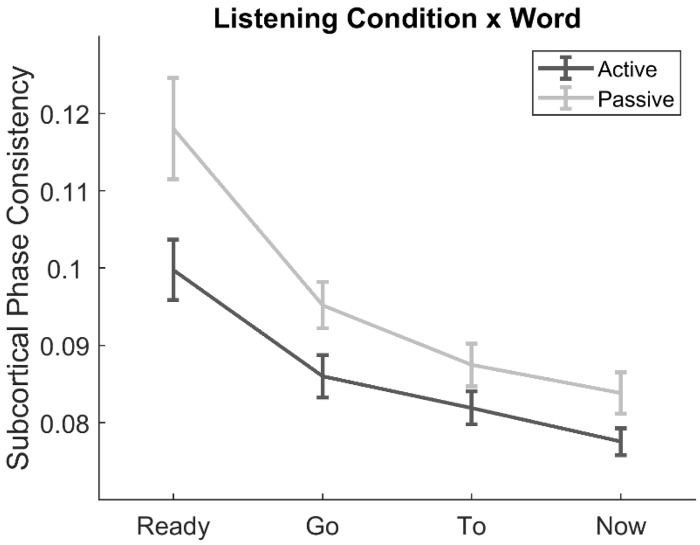


**Supplementary Figure 15.** Listening condition by word interaction. Subcortical phase consistency is plotted on the y-axis, and word is plotted on the left axis. Active is plotted in dark gray and passive is plotted in light gray. Although passive tended to be more consistent than active, comparisons at each of the words did not survive correction for multiple comparisons.

| | **Active** | **Passive** | **r** | **t** | **p** | **d** |
|---|---|---|---|---|---|---|
| **Ready** | 0.100 ± 0.025 | 0.118 ± 0.041 | .101 | 2.509 | .016 | 0.397 |
| **Go** | 0.086 ± 0.017 | 0.095 ± 0.019 | .044 | 2.315 | .026 | 0.366 |
| **To** | 0.082 ± 0.014 | 0.087 ± 0.018 | .154 | 1.714 | .094 | 0.271 |
| **Now** | 0.078 ± 0.011 | 0.084 ± 0.017 | .162 | 2.148 | .038 | 0.34 |

**Supplementary Table 16.** Listening condition by word post hoc tests. Degrees of freedom are 39.

### 1.2.4 Stimulus (pitch contour) by Electrode Interaction

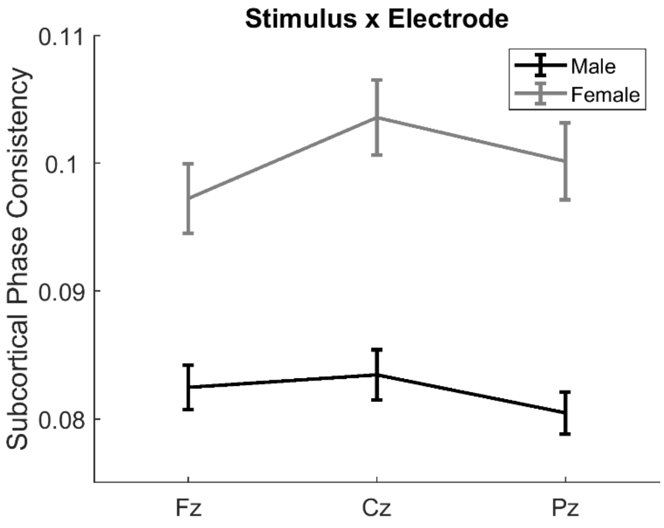


**Supplementary Figure 16.** Stimulus (pitch contour) by electrode interaction. Subcortical phase consistency is plotted on the y-axis and electrode is plotted on the x-axis. The male pitch contour is plotted in black and the female is plotted in gray. Consistent with the findings reported above, for the female stimulus, the consistency was highest at Cz (Cz v. Fz $t(39) = 8.100$, $p < .0005$, $d = 1.281$; Cz v. Pz $t(39) = 4.125$, $p < .0005$, $d = 0.652$), and for the male stimulus, Cz was higher than Pz ($t(39) = 5.189$, $p < .0005$, $d = 0.820$), but did not differ from Fz ($t(39) = 1.400$, $p = .170$, $d = 0.221$).

| | Male | Female | r | t | p | d |
|---|---|---|---|---|---|---|
| **Fz** | **0.082 ± 0.011** | **0.097 ± 0.017** | **.6900** | **7.507** | **<.0005** | **1.187** |
| **Cz** | **0.083 ± 0.012** | **0.104 ± 0.019** | **.7069** | **9.720** | **<.0005** | **1.537** |
| **Pz** | **0.080 ± 0.011** | **0.100 ± 0.019** | **.6809** | **8.794** | **<.0005** | **1.390** |

**Supplementary Table 17.** Stimulus (pitch contour) by electrode means ± 1 standard deviation and post hoc tests. Degrees of freedom are 39.

### 1.2.5 Stimulus (pitch contour) by Word Interaction

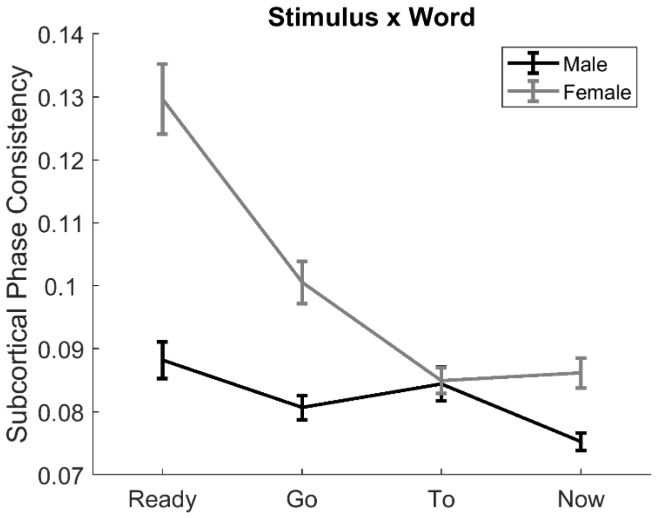


**Supplementary Figure 17.** Stimulus (pitch contour) by word interaction. Subcortical phase consistency is plotted on the y-axis and word is plotted on the x-axis. The male pitch contour is plotted in black and the female is plotted in gray. Comparing the male and female pitch contours for each word, the response to the female had higher consistency at ‘ready’, ‘go’ and ‘now’, while responses to the male and female were matched at ‘to’.

| | Male | Female | r | t | p | d |
|---|---|---|---|---|---|---|
| **Ready** | **0.088 ± 0.018** | **0.130 ± 0.035** | **.745** | **10.558** | **<.0005** | **1.669** |
| **Go** | **0.081 ± 0.012** | **0.101 ± 0.021** | **.164** | **5.523** | **<.0005** | **0.873** |
| **To** | 0.084 ± 0.017 | 0.085 ± 0.013 | .261 | 0.191 | .849 | 0.030 |
| **Now** | **0.075 ± 0.009** | **0.086 ± 0.015** | **.595** | **5.677** | **<.0005** | **0.898** |

**Supplementary Table 17.** Stimulus (pitch contour) by word means ± 1 standard deviation and post hoc tests. Degrees of freedom are 39.

### 1.2.6 Word by Electrode Interaction

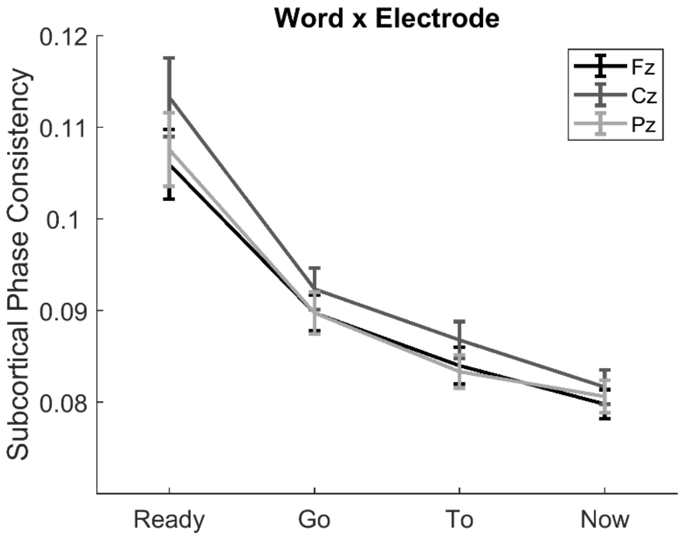


**Supplementary Figure 18.** Word by electrode interaction. Subcortical phase consistency is plotted on the y-axis and word is plotted on the x-axis. Fz is plotted in black, Cz is plotted in dark gray, and Pz is plotted in light gray. Comparing the three electrodes at each word, Cz had more consistency at 'ready' than Fz or Pz, as well as higher consistency over 'to' relative to Pz.

| | | | | Fz - Cz | | | | Fz - Pz | | | | Cz - Pz | | | |
|---|---|---|---|---|---|---|---|---|---|---|---|---|---|---|---|
| | **Fz** | **Cz** | **Pz** | **r** | **t** | **p** | **d** | **r** | **t** | **p** | **d** | **r** | **t** | **p** | **d** |
| **Ready** | 0.106 ± 0.024 | 0.113 ± 0.027 | 0.108 ± 0.025 | **.969** | **6.565** | **<.0005** | **1.038** | .947 | 1.247 | .220 | 0.197 | **.965** | **5.100** | **<.0005** | **0.806** |
| **Go** | 0.090 ± 0.012 | 0.092 ± 0.014 | 0.090 ± 0.014 | .854 | 2.243 | .031 | 0.355 | .853 | 0.018 | .985 | 0.003 | .913 | 2.764 | .009 | 0.437 |
| **To** | 0.084 ± 0.013 | 0.087 ± 0.013 | 0.083 ± 0.011 | .884 | 2.867 | .007 | 0.453 | .876 | 0.685 | .497 | 0.108 | **.930** | **4.677** | **<.0005** | **0.739** |
| **Now** | 0.080 ± 0.010 | 0.082 ± 0.012 | 0.081 ± 0.011 | .917 | 2.493 | .017 | 0.394 | .901 | 1.080 | .287 | 0.171 | .962 | 2.041 | .048 | 0.323 |

**Supplementary Table 18.** Word by electrode means ± 1 standard deviation and post hoc tests. Degrees of freedom are 39.

### 1.2.7 Listening Condition by Stimulus (pitch contour) by Word Interaction

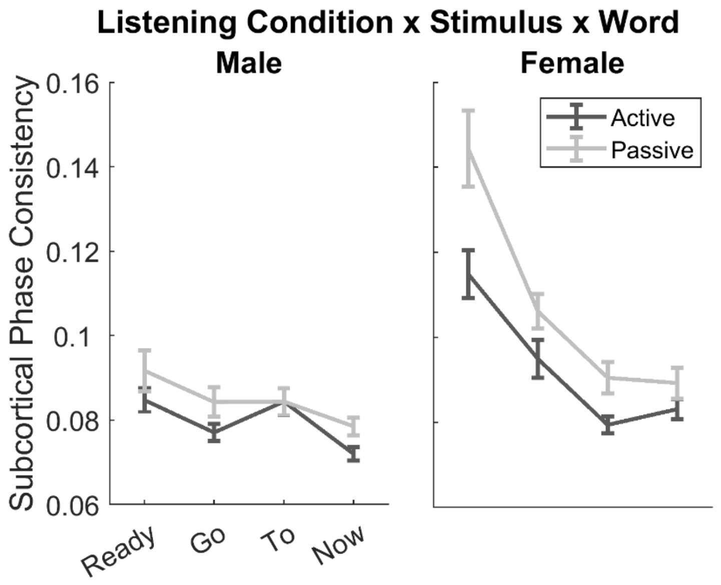


**Supplementary Figure 19.** Listening condition by stimulus (pitch contour) by word interaction. Subcortical phase consistency is plotted on the y-axis and word is plotted on the x-axis. The male pitch contour is plotted on the left and the female is plotted on the right. Active is plotted in dark gray and passive is plotted in light gray. Both graphs are plotted on the same scale. The effects were consistent with the main effects and 2-way interactions. That is, the greatest consistency was seen over the 'ready' in response to the female F0. While the comparison to the passive 'ready' of the female did not survive multiple corrections ($t(39) = 2.980$, $p = .005$, $d = 0.471$), the remaining comparisons did (all t(39)'s $\geq$ 4.963, all p's < .00002, all d's $\geq$ 0.785).

| | | **Ready** | **Go** | **To** | **Now** |
|---|---|---|---|---|---|
| **Male** | **Active** | 0.085 ± 0.018 | 0.077 ± 0.013 | 0.084 ± 0.020 | 0.072 ± 0.010 |
| | **Passive** | 0.092 ± 0.031 | 0.084 ± 0.022 | 0.084 ± 0.020 | 0.078 ± 0.013 |
| **Female** | **Active** | 0.115 ± 0.035 | 0.095 ± 0.028 | 0.079 ± 0.013 | 0.083 ± 0.015 |
| | **Passive** | 0.144 ± 0.057 | 0.106 ± 0.025 | 0.090 ± 0.023 | 0.089 ± 0.023 |

**Supplementary Table 19.** Listening condition by stimulus (pitch contour) by word means $\pm$ 1 standard deviation.

### 1.2.8 Stimulus (pitch contour) by Word by Electrode Interaction

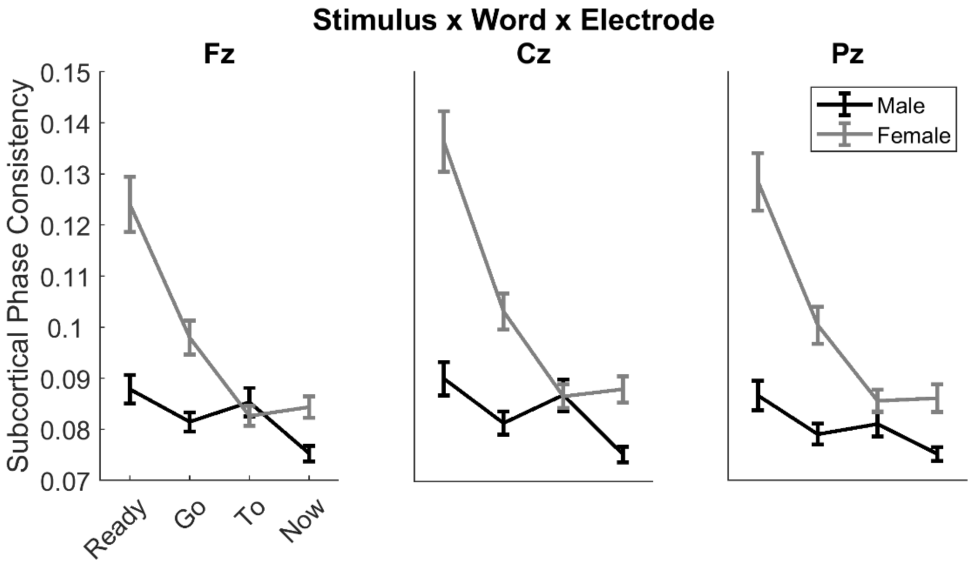


**Supplementary Figure 20.** Stimulus (pitch contour) by word by electrode interaction. Subcortical phase consistency is plotted on the y-axis and word is plotted on the x-axis. From left to right are plotted Fz, Cz, and Pz. The male pitch contour is plotted in black and the female is plotted in gray. All graphs are plotted on the same scale. The effects were consistent with the main effects and 2-way interactions. The greatest consistency was in response to 'ready' of the female, showing higher consistency than any other subcortical response, including the response to female 'ready' at Fz ($t(39) = 7.596$, $p < .0002$, $d = 1.201$) and Pz ($t(39) = 4.480$, $p < .0002$, $d = 0.710$) all other tests ($t(39)$'s $\geq$ 8.744, all p's < .0002, all d's $\geq$ 1.201.

| | | Ready | Go | To | Now |
|---|---|---|---|---|---|
| | **Fz** | 0.088 ± 0.018 | 0.081 ± 0.012 | 0.085 ± 0.018 | 0.075 ± 0.009 |
| **Male** | **Cz** | 0.090 ± 0.021 | 0.081 ± 0.014 | 0.087 ± 0.020 | 0.075 ± 0.010 |
| | **Pz** | 0.087 ± 0.018 | 0.079 ± 0.013 | 0.081 ± 0.016 | 0.075 ± 0.009 |
| | **Fz** | 0.124 ± 0.034 | 0.098 ± 0.021 | 0.083 ± 0.013 | 0.084 ± 0.013 |
| **Female** | **Cz** | 0.136 ± 0.037 | 0.103 ± 0.022 | 0.087 ± 0.015 | 0.088 ± 0.016 |
| | **Pz** | 0.128 ± 0.036 | 0.100 ± 0.023 | 0.086 ± 0.014 | 0.086 ± 0.017 |

**Supplementary Table 20.** Stimulus (pitch contour) by word by electrode means ± 1 standard deviation.

### 1.2.9 Condition by Stimulus (pitch contour) by Word by Electrode Interaction

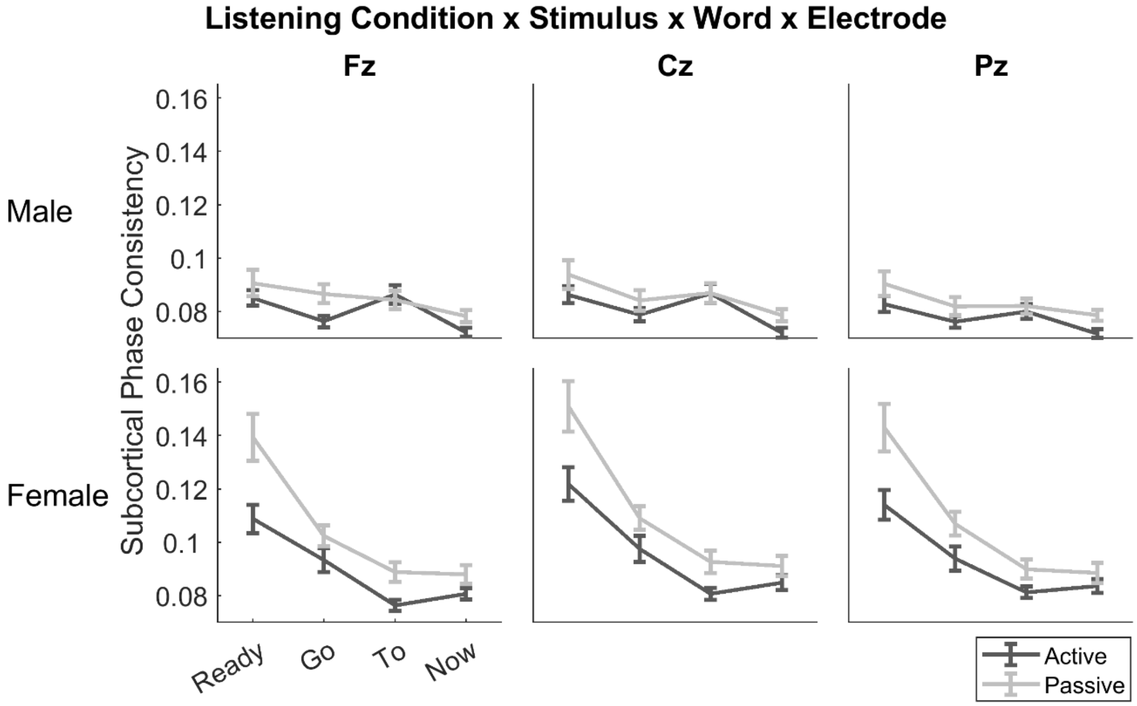


**Supplementary Figure 21.** Listening condition by stimulus (pitch contour) by word by electrode interaction. Subcortical phase consistency is plotted on the y-axis and word is plotted on the x-axis. From left to right are plotted Fz, Cz, and Pz. The male pitch contour is plotted on the top row and the female is plotted on the bottom. Active is plotted in dark gray and passive is plotted in light gray. All graphs are plotted on the same scale. The effects were consistent with the main effects and 2- and 3-way interactions. Due to the number of significant comparisons that remained after controlling for multiple comparisons (~300 of the ~1100) and that the results replicate the simpler comparisons described above and in the main text, the post hocs are not reported here.

**Fz**

| | | **Ready** | **Go** | **To** | **Now** |
|---|---|---|---|---|---|
| **Male** | **Active** | 0.085 ± 0.018 | 0.076 ± 0.014 | 0.086 ± 0.022 | 0.072 ± 0.010 |
| | **Passive** | 0.091 ± 0.031 | 0.087 ± 0.023 | 0.084 ± 0.022 | 0.078 ± 0.014 |
| **Female** | **Active** | 0.109 ± 0.033 | 0.093 ± 0.029 | 0.076 ± 0.013 | 0.081 ± 0.013 |
| | **Passive** | 0.139 ± 0.056 | 0.102 ± 0.024 | 0.089 ± 0.023 | 0.088 ± 0.022 |

**Cz**

| | | Ready | Go | To | Now |
|---|---|---|---|---|---|
| **Male** | **Active** | 0.086 ± 0.021 | 0.079 ± 0.015 | 0.087 ± 0.023 | 0.072 ± 0.011 |
| | **Passive** | 0.094 ± 0.034 | 0.084 ± 0.024 | 0.087 ± 0.023 | 0.079 ± 0.014 |
| **Female** | **Active** | 0.122 ± 0.039 | 0.098 ± 0.030 | 0.081 ± 0.014 | 0.085 ± 0.017 |
| | **Passive** | 0.151 ± 0.060 | 0.109 ± 0.028 | 0.093 ± 0.026 | 0.091 ± 0.024 |

| | | | Pz | | |
|---|---|---|---|---|---|
| | | **Ready** | **Go** | **To** | **Now** |
| **Male** | **Active** | 0.083 ± 0.019 | 0.076 ± 0.014 | 0.080 ± 0.018 | 0.072 ± 0.011 |
| | **Passive** | 0.090 ± 0.029 | 0.082 ± 0.021 | 0.082 ± 0.018 | 0.079 ± 0.014 |
| **Female** | **Active** | 0.114 ± 0.035 | 0.094 ± 0.029 | 0.081 ± 0.014 | 0.084 ± 0.017 |
| | **Passive** | 0.143 ± 0.057 | 0.107 ± 0.028 | 0.090 ± 0.023 | 0.089 ± 0.024 |

**Supplementary Table 21.** Listening condition by stimulus (pitch contour) by word by electrode means ± 1 standard deviation.

### 1.2.10 Condition by Stimulus (pitch contour) by Word by Electrode by Language Group Interaction

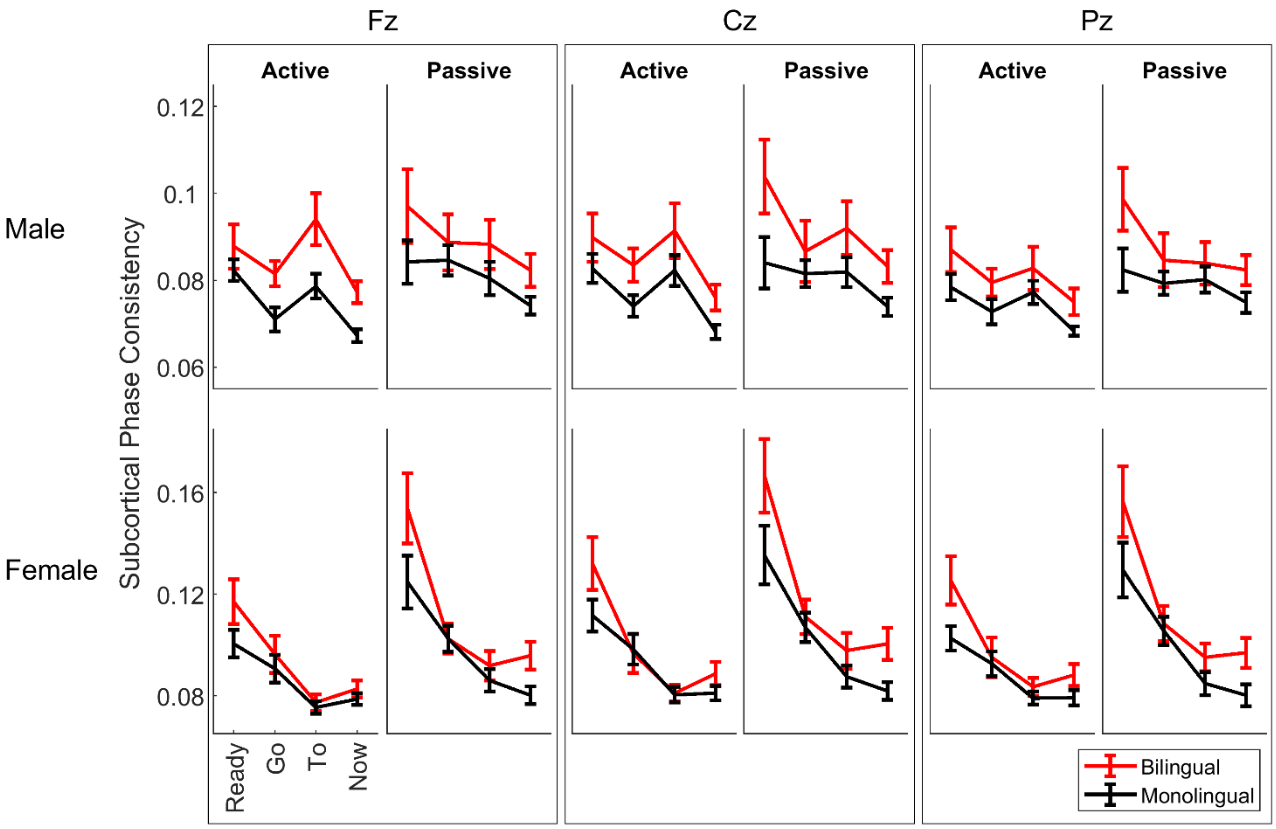


**Supplementary Figure 22.** Although the 5-way interaction was not significant, it is plotted here for visualization purposes. No statistics were run on these values. Subcortical phase consistency is on the y axis, and word is on the x axis, with monolinguals plotted in black and bilinguals plotted in red. From left to right are plotted Fz Active, Fz passive, Cz active, Cz passive, Pz active and Pz passive, with the male pitch contour plotted in the top row and the female pitch contour in the bottom row. All graphs for male pitch contour are on the same scale and all graphs for the female pitch contour are on the same scale. Values are indicated by the y axis values on the leftmost plots.
